# A scoping review of stem cell models of leukodystrophies: advances in understanding pathophysiological mechanisms

**DOI:** 10.1038/s41525-025-00533-0

**Published:** 2025-11-28

**Authors:** Alexandra Chapleau, Stefanie Perrier, Thomas M. Durcan, Geneviève Bernard

**Affiliations:** 1 Department of Neurology and Neurosurgery, McGill University, Montréal, QC Canada; 2 Child Health and Human Development Program, Research Institute of the McGill University Health Centre, Montréal, QC Canada; 3 The Neuro’s Early Drug Discovery Unit (EDDU), McGill University, Montréal, QC Canada; 4 Departments of Pediatrics and Human Genetics, McGill University, Montréal, QC Canada; 5 Department of Specialized Medicine, Division of Medical Genetics, Montreal Children’s Hospital and McGill University Health Centre, Montréal, QC Canada

**Keywords:** Experimental models of disease, Disease genetics

## Abstract

Leukodystrophies are a diverse group of genetic disorders affecting the central nervous system white matter. Since their initial identification over a century ago, significant advancements have been made in understanding their genetic and clinical profiles. Yet, disease modifying therapies are limited, despite significant clinical impact characterized by progressive neurological decline leading to severe disability and early mortality. This underscores the need for advanced disease models to facilitate the understanding of disease mechanisms and the development of early therapeutic interventions. Stem cells have emerged as a transformative tool in leukodystrophy research, enabling the generation of patient-specific cells otherwise inaccessible for study. We have conducted the first scoping review of stem cell-based disease modeling in leukodystrophies, highlighting recent developments, challenges, and future directions in leveraging these models to enhance our understanding and aid in the development of therapies for these debilitating disorders.

## Introduction

Leukodystrophies are a group of rare inherited brain white matter disorders with heterogeneous genotypic and phenotypic profiles. There are over 100 types of distinct leukodystrophies, with a combined incidence estimated to be approximately 1 in 4700 live births^1,2^. While most leukodystrophies have a disease onset during childhood, a wide spectrum of severity exists between subtypes of leukodystrophies. The most debilitating clinical features typically result from neurodegeneration, leading to progressive disability, including loss of motor function, cognitive decline, and early death. Limited disease-modifying therapies are available, and therefore, treatment usually revolves around managing symptoms to improve quality of life. Therefore, it is imperative to develop models to both study the disease and facilitate the development of therapies.

The definition and classification of leukodystrophies have long been subjects of debate, evolving alongside advancements in our understanding of their genetic origins and pathophysiology. Traditionally, leukodystrophies were defined as genetic disorders resulting primarily from myelin development or maintenance, whereby genetic disorders in which white matter abnormalities arose secondary to another underlying pathology (e.g., primary neuronal, vascular, etc.) were typically referred to as genetically determined leukoencephalopathies^1^. This original distinction has blurred in recent years with the discovery of new leukodystrophy-associated genes and mechanisms. The most recent framework now includes all inherited disorders that primarily affect all cell types from the central nervous system white matter, thereby encompassing both classical leukodystrophies and leukoencephalopathies^3^.

Historically, leukodystrophies were classified based on myelin-related pathology, relating to whether the diseases were associated with lack of initial myelin deposition (i.e., hypomyelinating leukodystrophies), deposition of abnormal myelin (i.e., dysmyelinating leukodystrophies), loss of deposited myelin (e.g., demyelinating leukodystrophies), or myelin vacuolization (i.e., myelinolytic diseases)^4^. Using a more recent and faithful classification based on disease pathophysiology, leukodystrophies can be categorized based on the primary white matter component or cell type involved, such as myelin disorders, leuko-axonopathies, astrocytopathies, microgliopathies, or leuko-vasculopathies. As leukodystrophies can be complex in their molecular and cellular disease mechanisms, it is possible that some diseases fall within more than one category^5^.

Due to the complexity of their underlying molecular disease mechanisms, variability of cell types associated with pathophysiology, and accessibility of human brain tissue, leukodystrophies are challenging diseases to study. Access to pathological tissue for research is also typically rare and restricted to end-stage disease, making investigation of longitudinal pathology throughout disease evolution impossible. Therefore, alternative approaches such as disease-specific experimental models are necessary. Animal models, primarily involving murine models, have significantly contributed to our knowledge of neurological diseases and the development of potential therapeutic strategies. However, these models often fail to fully recapitulate the phenotypic complexities, including white matter pathology, and disease progression observed in human neurodegenerative disorders. Moreover, they may exhibit more severe manifestations (e.g., embryonic lethality), only replicate some aspects of the disorder, or lack a clinically relevant phenotype altogether^6–17^. To recapitulate features of the human disease, animal models often rely on more severe genetic alterations than those observed in human disorders, including but not limited to complete gene knock-outs, partial deletions, or additional genetic modifications (e.g., conditional expression under specific promoters, modifier genes)^18–22^. Although such genetic manipulations can force animals to display a phenotype more closely matching the human disease, the underlying pathophysiological mechanisms may be impacted in a less relevant manner. For white matter disorders specifically, additional limitations associated with the use of rodents to model human diseases include disparities in brain anatomy, development, cell function, and biochemistry. Specifically, humans and rodents differ in the degree of tissue complexity, level of myelination, and glial cell gene expression profiles^23–25^.

**Fig. 1:**
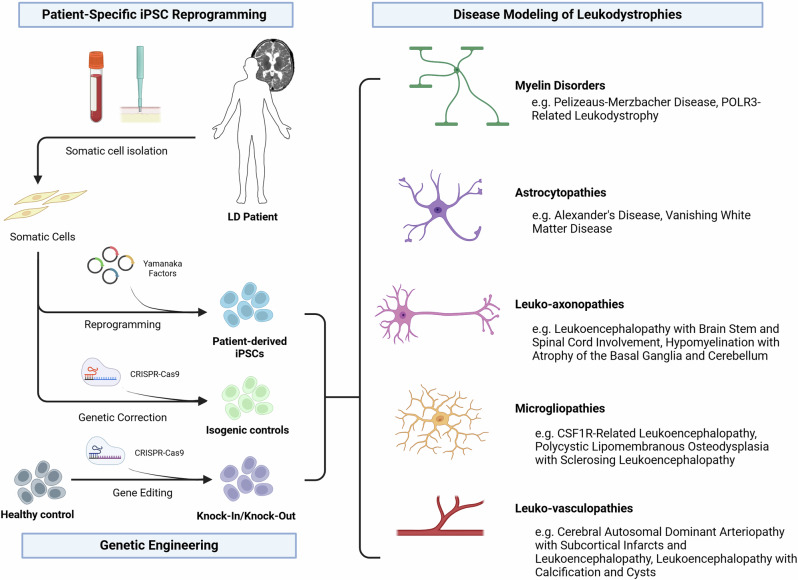
Overview of patient-specific stem cell generation and their applications in disease modeling for leukodystrophies. Created in BioRender. https://BioRender.com/589zmk2.

In vitro cellular models circumvent many of the limitations posed by murine models by providing a physiologically relevant and cell-specific platform to study disease phenotypes. Stem cells, including both embryonic stem cells (ESC) and induced pluripotent stem cells (iPSCs), have been increasingly utilized in modeling neurological disorders, as they allow the in vitro study of otherwise inaccessible cell types pathologically associated with the disease. The differentiation protocols can be designed to mimic the developmental processes occurring in vivo, allowing for the exploration of disease pathology at different stages in development, making them particularly useful for the study of pediatric neurodevelopmental disorders. These disease-in-a-dish models have been invaluable for characterizing disease pathophysiology and investigating potential therapeutic avenues (Fig. 1).

Although both ESCs and iPSCs are powerful tools for disease modeling, ethical concerns and accessibility challenges can limit the use of ESCs in research, as they are derived from early-stage embryos. iPSCs provide an alternative by reprogramming somatic cells into a pluripotent state. This not only circumvents ethical issue but allows for patient-specific disease modeling, making iPSCs a more versatile and widely applicable tool in discovery research. iPSCs are generated by reprogramming somatic cells using four main factors, most notably the Yamanaka factors: Oct3/4, Sox2, Klf4 and c-Myc^26,27^. These factors can be delivered to somatic cells in various modes, including retroviruses, inducible lentiviruses, mRNAs, DNA episomes, peptides, and non-integrating viruses (i.e., Sendai virus). After being reprogrammed to a stem-cell state, iPSCs have the capability to be differentiated into target cells of interest, facilitating the study of tissue-specific physiology. This technology has been revolutionary for the rare disease field in particular, where many orphan diseases remain under-characterized and poorly understood due to limitations in tissue and cell availability. iPSCs can be reprogrammed from a variety of somatic cells, such as fibroblasts and peripheral blood mononuclear cells (PBMCs), which are non-invasive and easy to obtain in the clinical setting. As iPSCs are inherently pluripotent, they can act as an inexhaustible resource of relevant biological material, with the ability to be frozen, thawed, and expanded indefinitely to generate scalable disease model banks for future therapy testing, once appropriate quality control methods are followed^28^. As iPSCs are derived directly from patient cells, personalized disease models can be generated containing the same pathogenic genetic variants and specific genomic signatures of the patient, as potential modifier genes may contribute to overall pathogenesis.

Given the rapid expansion of research using stem cell models for leukodystrophies, we elected to conduct a scoping review to critically examine the scope and focus of existing leukodystrophy stem cell research. In this review, we aimed to summarize and critically assess current research utilizing stem cell-based models of leukodystrophies, highlighting the strategies that have been used to generate ESC and iPSC-derived cell models to phenocopy the human disease and discuss the benefits and limitations of using these cells to explore leukodystrophy disease mechanisms and investigate the development of novel therapeutic avenues.

## Results

### Search results

In accordance with the PRISMA-ScR framework, the search yielded a total of 4858 records (Supplementary Fig. 1). Following the removal of 2214 duplicates, 2644 studies underwent title and abstract screening, resulting in the exclusion of 2290 irrelevant studies. The remaining 354 studies underwent full-text review, leading to the exclusion of 162 articles that did not meet eligibility criteria. Ultimately, 192 studies were included in the final analysis. Inter-rater agreement for the title and abstract screening was 0.807, and 0.923 for the full-text review, reflecting high levels of agreement between reviewers. Additionally, 2 articles, identified through manual reference searches, were included in the review as they met all inclusion criteria, bringing the total number of studies included to 194 (Supplementary Fig. 1).

### Description of included studies

**Fig. 2:**
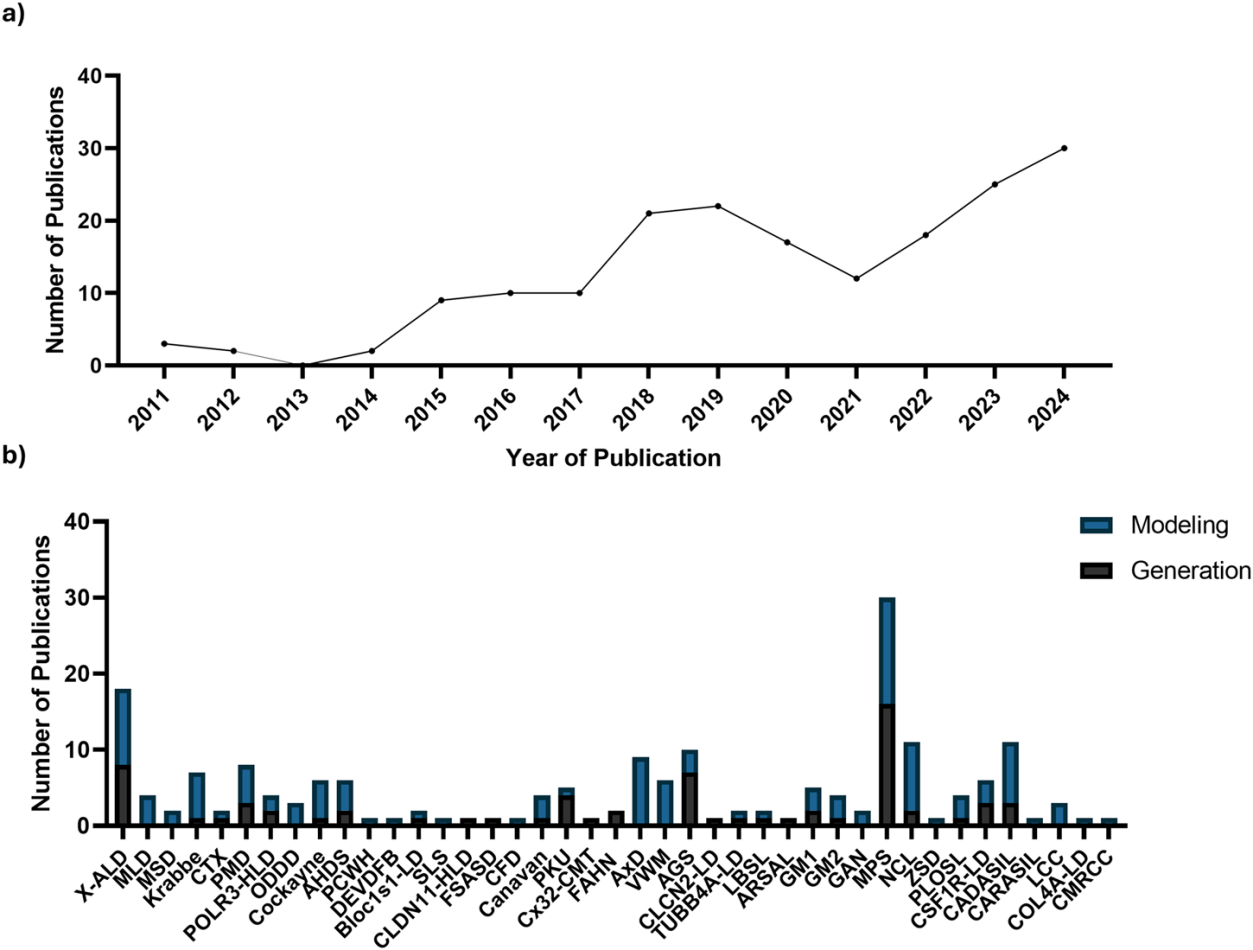
Overview of leukodystrophy stem cell publications. **a** Number of leukodystrophy stem cell publications per year, illustrating the growing interest in stem cell-based modeling. **b** Number of publications per leukodystrophy included in the review. Bars are categorized by studies focused on disease modeling (blue) versus those reporting iPSC generation only (black). All disorders shown were included in the review, though only a subset are discussed in detail in the main text. Figure created using GraphPad Prism Version 10.5.0. X-ALD adrenoleukodystrophy, MLD metachromatic leukodystrophy, MSD Multiple Sulfatase Deficiency, CTX Cerebrotendinous xanthomatosis, SLS Sjögren–Larsson syndrome, PMD Pelizaeus-Merzbacher disease, POLR3-HLD POLR3-related leukodystrophy, ODDD Oculodentodigital dysplasia, AHDS Allan-Herndon-Dudley syndrome, PCWH peripheral demyelinating neuropathy, central dysmyelination, Waardenburg syndrome, and Hirschsprung disease, DEVDFB developmental delay, dysmorphic facies, and brain anomalies, Bloc1s1-LD Bloc1s1-related leukodystrophy, CLDN11-HLD CLDN11-related leukodystrophy, FSASD Free Sialic Acid Storage Disorders, CFD cerebral folate deficiency, PKU Phenylketonuria, Cx32-CMT Cx32-related (X-linked) Charcot-Marie-Tooth disease, FAHN fatty acid hydroxylase-associated neurodegeneration, AxD Alexander disease, VWM Vanishing White Matter, AGS Aicardi-Goutières syndrome, CLCN2-LD CLCN2-related leukoencephalopathy, TUBB4A-LD TUBB4A-related leukodystrophy, LBSL leukoencephalopathy with brainstem and spinal cord involvement and lactate elevation, ARSAL autosomal recessive spastic ataxia with leukoencephalopathy, GM1 GM1 gangliosidosis, GM2 GM2 gangliosidosis, GAN giant axonal neuropathy, MPS mucopolysaccharidoses, NCL neuronal ceroid lipofuscinoses, ZSD Zellweger Spectrum Disorder, PLOSL polycystic lipomembranous osteodysplasia with sclerosing leukoencephalopathy, CSF1R-LD CSF1R-related leukoencephalopathy, CADASIL cerebral autosomal dominant arteriopathy with subcortical infarcts and leukoencephalopathy, CARASIL cerebral autosomal recessive arteriopathy with subcortical infracts and leukoencephalopathy, LCC leukoencephalopathy with brain calcifications and cysts, COL4A-LD COL4A-related disorders, CMRCC cerebroretinal microangiopathy with calcifications and cysts.

The number of published reports using stem cells to model leukodystrophies has steadily increased since the discovery of iPSCs in 2006, reflecting growing interest in the application of stem cell technologies to study these disorders (Fig. 2). Notably, there was a decline in publications in 2020, coinciding with the onset of the COVID-19 pandemic and the widespread disruption to research activities. Despite this, most included studies were published between 2018 and 2024.

**Fig. 3:**
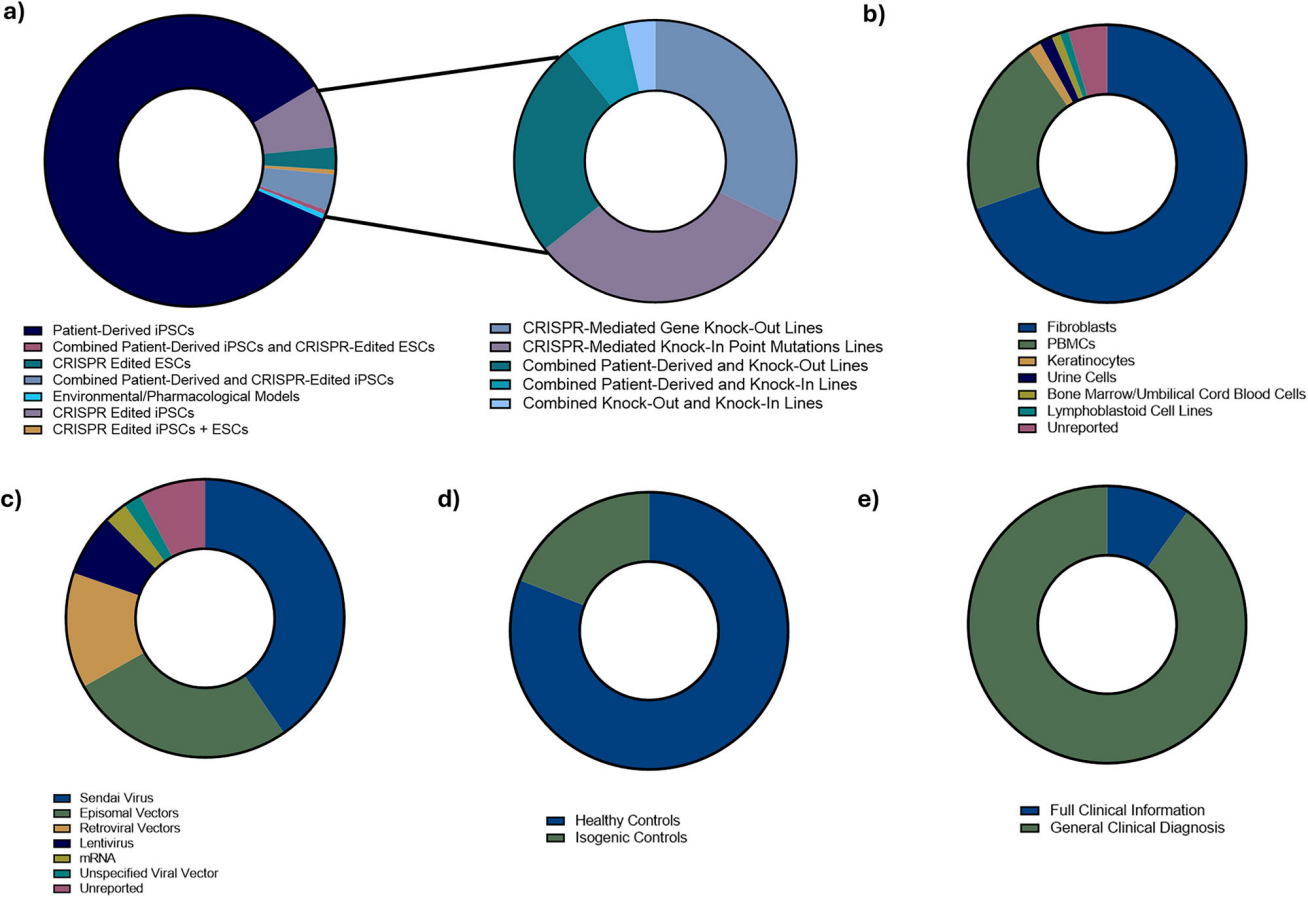
Overview of the characteristics of leukodystrophy stem cell models used in published studies. **a** Distribution of studies by stem cell source and type of genetic modification utilized. **b** Somatic cell types used for reprogramming. **c** Methods employed to reprogram cells into iPSCs. **d** Types of control samples included. **e** Availability of patient clinical information. All data is summarized using circular bar plots, and values represent the percentage of publications (out of total included studies) that report each feature. Figure created using GraphPad Prism Version 10.5.0. iPSCs Induced pluripotent stem cells, ESC embryonic stem cells, CRISPR clustered regularly interspaced short palindromic repeats, PBMCs peripheral blood mononuclear cells.

Among the studies, 64.94% (n = 126) focused on disease modeling (Table 1), while 35.05% (n = 68) were reports on the generation and characterization of iPSCs, without further downstream differentiation or phenotypic examination (Supplementary Table 2). iPSCs were the most common stem cell utilized (97.42%, n = 189), with only 5 (2.577%) studies reporting the use of ESCs and one study that used a combination of both. Specifically, patient-derived iPSCs were the predominant model (86.08%, n = 167), with 82.54% (n = 104) used in disease modeling publications. In contrast, genome-editing approaches were less common (14.43%, n = 28), encompassing both knock-in (4.639%, n = 9) and knock-out (4.124%, n = 8) mutations. A small number of studies (4.639%, n = 9) combined patient-derived iPSCs with CRISPR-edited knock-out (3.308%, n = 7) or knock-in (1.031%, n = 2) lines. Additionally, one study used a combination of both knock-out and knock-in iPSC lines, while another used control iPSCs exposed to phenylalanine to model PKU. iPSCs were primarily derived from fibroblasts (70.62%, n = 137) or PBMCs (21.13%, n = 41), with the remainder generated from keratinocytes (1.546%, n = 3), urine cells (1.546%, n = 3), bone marrow or umbilical cord blood (1.030%, n = 2), or lymphoblastoid cell lines (LCLs) (1.030%, n = 2). Nine studies (4.639%) did not report the cell type used for derivation. Reprogramming methods varied, with most studies using Sendai virus (40.21%, n = 78), followed by episomal vectors (26.29%, n = 51), retroviral vectors (12.89%, n = 25), lentivirus (7.216%, n = 14), and mRNA reprogramming (2.577%, n = 5). Four studies utilized viral reprogramming without specifying the specific method, and one study used overexpression without further details. Fifteen studies (7.732%) did not specify the reprogramming method used. For disease modeling studies involving patient-derived iPSCs, most studies (80.95%, n = 102) included healthy donor-derived iPSCs as controls, while 19.05% (n = 24) generated isogenic controls by correcting the pathogenic variant(s) in patient-derived lines (Fig. 3).

**Table 1:** Summary of iPSC models of leukodystrophies generated to date

Disease	Gene(s)	Phenotype/Genotype	Reprogramming Method(s)	Differentiation protocols	Control Types	References
**Myelin Disorders**						
*Demyelinating Disorders*						
ALD	*ABCD1*	1. Pd iPSC, ccALD: *ABCD1* (HEMI), exon 8-10 deletion^a^ 2. Pd iPSC, AMN: *ABCD1* (HEMI), exon 7-10 deletion^b^	Retrovirus (Fibroblasts)	Neurons: Kim et al.^459^; Cho et al.^460^ OLs: Kang et al.^461^	Healthy controls	Jang et al.^63^
		1. Pd iPSC, ccALD: *ABCD1* (HEMI), c.253_254insC (p.P84) 2. Pd iPSC, ccALD: *ABCD1* (HEMI), c.1847C>T (p.A616V)	Retrovirus (Fibroblasts)	iPSCs	Healthy controls	Wang et al.^62^
		1. Pd iPSC, ccALD: *ABCD1* (HEMI), UNK 2. Pd iPSC, AMN: *ABCD1* (HEMI), UNK^c^	Lentivirus (Fibroblasts)	Neurons, OLs, Astrocytes: Doetschman et al.^462^; Hopfl et al.^463^	Healthy controls	Baarine et al.^65^
		1. Pd iPSC, ccALD: *ABCD1* (HEMI), exon 8-10 deletion^a^ 2. Pd iPSC, AMN: *ABCD1* (HEMI), exon 7-10 deletion^b^	Retrovirus (Fibroblasts)	OLs: Kang et al.^461^	Healthy control	Jang et al.^64^
		1. Pd iPSC, ccALD: *ABCD1* (HEMI), UNK 2. Pd iPSC, ccALD: *ABCD1* (HEMI), UNK 3. Pd iPSC, ccALD: *ABCD1* (HEMI), UNK	Retrovirus (Fibroblasts, Keratinocytes)	cOrg: own protocol	Healthy controls	Lindborg et al. ^464^
		1. Pd iPSC, ccALD: *ABCD1* (HEMI), UNK 2. Pd iPSC, ccALD: *ABCD1* (HEMI), UNK 3. Pd iPSC, ccALD: *ABCD1* (HEMI), UNK	Retrovirus (Fibroblasts, Keratinocytes)	BMECs: Stebbins et al.^465^	Healthy controls	Lee et al.^79^
		1. Pd iPSC, ccALD: *ABCD1* (HEMI), exon 8-10 deletion^a^ 2. Pd iPSC, AMN: *ABCD1* (HEMI), exon 7–10 deletion^b^	Retrovirus (Fibroblasts)	OLs: Kang et al.^461^	Healthy control	Jang et al.^71^
		1. Pd iPSC, ccALD: *ABCD1* (HEMI), UNK^d^ 2. Pd iPSC, AMN: *ABCD1* (HEMI), UNK^c^	Sendai virus (Fibroblasts)	Astrocytes: Lundin et al.^466^	Healthy control	Parasar et al.^66^
		1. Pd iPSC, ccALD: *ABCD1* (HEMI), UNK^d^ 2. Pd iPSC, ccALD: *ABCD1* (HEMI), UNK 3. Pd iPSC, AMN: *ABCD1* (HEMI), UNK^c^ 4. Pd iPSC, AMN: *ABCD1* (HEMI), UNK	RNA (Fibroblasts)	Astrocytes: STEMCELL Technologies kit	Healthy controls	Kaur et al.^67^
		1. Pd iPSC, cerebral form: *ABCD1* (HEMI), c.1390 C > T (p.R464*) 2. Pd iPSC, cerebral form: *ABCD1* (HEMI), c.659 T > C (p.L220P) 3. Pd iPSC, cerebral form: *ABCD1* (HEMI), c.1866-10 G > A (p.P623fs*?) 4. Pd iPSC, AMN: *ABCD1* (HEMI), c.446 G > A (p.S149N)	Lentivirus (Fibroblasts)	Astrocytes: Nadadhur et al.^467^; Leferink et al.^292^ MNs: Du et al.^468^	Healthy controls	Ferrer et al.^72^
MLD	*ARSA*	1. Pd iPSC, Juvenile MLD: *ARSA* (HMZ), p.P426L	Retrovirus (Fibroblasts)	NPCs: Koch et al.^469^	Healthy control	Doerr et al.^98^
		1. Pd iPSC, UNK: *ARSA* (cHET), c.465+1 G > A (p.?); c.[1223_1231del9, 1055 A > G, *96 A > G] (p.S406_T408del)^e^ 2. Pd iPSC, Late infantile MLD: *ARSA* (HMZ), c.465+AG>A^f^	Lentivirus (Fibroblasts, PBMCs)	NPCs: Major et al.^470^ Neurons: Griffin et al.^471,472^ OPCs: Neri et al.^473^; Wang et al.^432^	Healthy controls	Meneghini et al.^95^
		1. Pd iPSC, UNK: *ARSA* (cHET), c.465+1 G > A (p.?); c.[1223_1231del9, 1055 A > G, *96 A > G] (p.S406_T408del)^e^ 2. Pd iPSC, Late infantile MLD: *ARSA* (HMZ), c.465+AG>A^f^	Lentivirus (Fibroblasts)	Neurons, Astrocytes, OPCs: Douvaras & Fossati et al.^104^	Healthy controls	Frati et al.^96^
		1. Pd iPSC, Late-infantile MLD: *ARSA* (UNK), UNK 2. Pd iPSC, Late-infantile MLD: *ARSA* (UNK), (UNK)	Episomal vectors (Fibroblasts)	MNPs, Motor Neurons: Okada et al.^103^	Healthy controls	Hossain et al.^97^
MSD	*SUMF1*	1. Pd iPSC, UNK: *SUMF1* (cHET), c.463 T > C (p.S155P); c.1034 G > A (p.R345H)	Sendai virus (PBMCs)	NPCs: Maguire et al.^473^	Healthy control	Schlotawa et al.^116^
		1. Pd iPSC, UNK: *SUMF1* (HMZ), c.836 C > T (p.A279V)	Sendai virus (PBMCs)	NPCs: Waxman et al.^474^ Neurons: Schafer et al.^475^; Ortiz et al. ^476^	Isogenic control	Pham et al.^115^
Krabbe	*GALC*	1. Pd iPSC, Infantile KD: *GALC* (HMZ), 30 kb deletion 2. Pd iPSC, Infantile KD: *GALC* (HMZ), c.1657 G > A	mRNA reprogamming (Fibroblasts)	NPCs: Chambers et al.^444^ Neurons, astrocytes, OLs: Frati et al.^96^	Healthy controls, unaffected carriers	Mangiameli et al.^132^
		1. Pd iPSC, Infantile KD: *GALC* (HMZ), 30 kb deletion 2. Pd iPSC, Infantile KD: *GALC* (HMZ), 30 kb deletion	Sendai virus (Fibroblasts)	NPCs: Li et al.^477^; Liu et al.^478^ Neurons: Li et al.^477^ Astrocytes: Roybon et al.^479^ MGL: Haenseler et al.^480^; van Wilgenburg et al.^446^; Brownjohn et al.^367^	Healthy controls	Lieberman et al.^134^
		1. Pd iPSC, UNK: *GALC* (cHET), c.461 C > A; c.1244 G>A^g^	Sendai virus (Fibroblasts)	NPCs: own protocol	Healthy control	Tian et al.^142^
		1. Pd iPSC, UNK: *GALC* (cHET), c.461 C > A; c.1244 G>A^g^	Sendai virus (Fibroblasts)	NPCs: Life Technologies Kit	Unaffected carrier	Lv et al.^133^
		1. Pd iPSC, Infantile KD: *GALC* (HMZ), 30 kb deletion 2. Pd iPSC, Infantile KD: *GALC* (HMZ), 30 kb deletion	UNK (Fibroblasts)	mOrg: James et al.^481^ MGL: Douvaras et al.^482^	Healthy controls	Evans et al.^141^
		1. Pd iPSC, UNK: *GALC* (cHET), c.461 C > A; c.1244 G>A^g^	Sendai virus (Fibroblasts)	cOrg: STEMCELL Technologies Kit	Healthy controls	Lv et al.^143^
CTX	*CYP27A1*	1. Pd iPSC, Early-onset: *CYP27A1* (cHET), c.397 T > C (p.W133R); c.183 C > T (p.R395C)	Episomal vectors (Fibroblasts)	Neurons: Boisvert et al.^483^; Li et al.^484^	Healthy controls	Mou et al.^154^
*Hypomyelinating Disorders*						
PMD	*PLP1*	1. Pd iPSC, Classic PMD: *PLP1* (HEMI), 16.2 kb duplication 2. Pd iPSC, Classic PMD: *PLP1* (HEMI), 636.9 kb partial *PLP1* duplication 3. Pd iPSC, Connatal PMD: *PLP1* (HEMI), c.636 G > C (p.W212C)	Retrovirus (Fibroblasts)	iPSCs	Healthy control	Shimojima et al.^186^
		1. Pd iPSC, Connatal PMD: *PLP1* (HEMI), c.757 T > A (p.S253T) 2. Pd iPSC, Classic PMD: *PLP1* (HEMI), c.643 C > T (p.P215S)	Retrovirus (Fibroblasts)	OLs: Kang et al.^461^; Izrael et al.^485^; Hu et al.^486^	Healthy controls	Numasawa-Kuroiwa et al.^187^
		1. Pd iPSC, Severe PMD: *PLP1* (HEMI), c.242 T > G (p.L81R) 2. Pd iPSC, Moderate PMD: *PLP1* (HEMI), c.254 T > G (p.L85R)^h^ 3. Pd iPSC, Mild PMD: *PLP1* (HEMI), c.441 A > T (p.G147 = ) 4. Pd iPSC, Moderate PMD: *PLP1* (HEMI), c.453 G > T (p.K151N) 5. Pd iPSC, Moderate PMD: *PLP1* (HEMI), c.453+750 G > A; (p.=) 6. Pd iPSC, Severe PMD: *PLP1* (HEMI), c.646 C > T (p.P216S) 7. Pd iPSC, Severe PMD: *PLP1* (HEMI), c.762+3 G > T (p.F233_L254del) 8. Pd iPSC, Severe PMD: *PLP1* (HEMI), c.762+3 G > T (p.F233_L254del) 9. Pd iPSC, Moderate PMD: *PLP1* (HEMI), c.352_499del (p.T118_V166del) 10. Pd iPSC, Moderate PMD: *PLP1* (HEMI), duplication^i^ 11. Pd iPSC, Severe PMD: *PLP1* (HEMI), triplication (3 copies) 12. Pd iPSC, Mild PMD: *PLP1* (HEMI), deletion^j^	Lentivirus and Episomal (Fibroblasts)	OLs: Douvaras & Fossati^104^	Healthy controls	Nevin et al.^188^
		1. Pd iPSC, Mild PMD: *PLP1* (HEMI), deletion^j^ 2. Pd iPSC, Moderate PMD: *PLP1* (HEMI), duplication^i^ 3. Pd iPSC, Severe PMD: *PLP1* (HEMI), c.254 T>G^h^	Lentivirus and Episomal (Fibroblasts)	mOrg: own protocol	Healthy controls	Madhavan et al.^189^
		1. Pd iPSC, Early-Severe PMD: *PLP1* (HEMI), p.G74E 2. Pd iPSC, Early-Severe PMD: *PLP1* (HEMI), p.T75P 3. Pd iPSC, UNK: *PLP1* (HEMI), p.F233L 4. Pd iPSC, Moderate PMD: *PLP1* (HEMI), PLP1 duplication^i^	Episomal (Fibroblasts)	OLs: Wang et al.^432^; Douvaras & Fossati^104^	Isogenic controls	Nobuta et al.^190^
POLR3-HLD	*POLR3A, POLR3B, POLR1C, POLR3D, POLR3K*	1. Pd iPSC, HSP: *POLR3A* (cHET), c.1909+22 G > A; p.Q31* 2. Pd iPSC, HSP: *POLR3A* (cHET) c.1909+22 G > A; p.Q31*	Sendai virus (Fibroblasts)	NPCs: own protocol	Healthy controls	Minnerop et al.^199^
		1. Pd iPSC, UNK: *POLR3A* (UNK), UNK 2. Pd iPSC, UNK: *POLR3A* (UNK), UNK 3. Pd iPSC, UNK: *POLR3A* (UNK), UNK 4. Pd iPSC, UNK: *POLR3B* (UNK), UNK 5. Pd iPSC, UNK: *POLR3B* (UNK), UNK 6. Pd iPSC, UNK: *POLR3B* (UNK), UNK	Lentivirus and sendai virus (Fibroblasts)	NPCs, Neurons: Holmes et al.^487^; Holmes et al.^488^ Nadadhur et al.^489^	Healthy controls	Dooves et al.^220^
ODDD	*GJA1*	1. Pd iPSC, UNK: GJA1 (HET), p.V216L^k^	Sendai virus (Fibroblasts)	NCC: Menendez et al.^490^; Huang et al.^491^ Osteoblast: StemPro Steogenesis kit Chondrocyte: StemPro Chondrogenesis kit	HET family member	Esseltine et al.^232^
		1. Pd iPSC, UNK: GJA1 (HET), p.V216L^k^	Sendai virus (Fibroblasts)	MSC, Adipocytes: StemDiff Mesenchymal Progenitor kit	Healthy control	Shao et al.^233^
		1. GE iPSC: *GJA1* (HET), KO	UNK (Fibroblasts)	Retinal organoids: Zhong et al.^492^	Unedited iPSCs	Cheng et al.^235^
Cockayne	*ERCC6, ERCC8, ERCC2, ERCC3*	1. Pd iPSC, UNK: *ERCC6* (HMZ), p.R735*	Retroviral (fibroblasts)	iPSCs	Healthy controls	Andrade et al.^493^
		1. Pd iPSC, CS Type I: *ERCC6* (cHET) c.1088 A > T; c.2648 C > T 2. Pd iPSC, CS Type I: *ERCC6* (HMZ) c.2282 C > T	Sendai virus (Fibroblasts)	Neurons: Marchetto et al.^494^; Griesi-Oliveira et al.^495^	Healthy controls	Vessoni et al.^496^
		1. Pd iPSC, UNK: *ERCC6* (cHET), c.643 G > T, (p.E215*); c.3776 C > A, (p.S1259*)	Episomal vectors (Fibroblasts)	NPCs: Liu et al.^478^; Duan et al.^497^ MSCs: Zhang et al.^498^; Pan et al.^499^; Wang et al.^500^	Isogenic controls	Wang et al.^501^
		1. Pd iPSC, COFS: *ERCC6* (HMZ), p.R683*^l^ 2. Pd iPSC, CS Type I: *ERCC6* (cHET), c.1131 A > T (p.K377*); c.2571 C > T (p.R857*)	Episomal plasmids (Fibroblasts)	NPCs: Martins et al.^502^; Martins et al.^503^; Sloan et al.^504^; Liu et al.^505^ cOrgs: Martins et al.^502^; Sloan et al.^504^	Healthy controls	Szepanowski et al.^506^
		1. Pd iPSC, COFS: *ERCC6* (HMZ), p.R683*^l^ 2. GE iPSC: *ERCC6* (HMZ), KO	Episomal plasmids (Fibroblasts)	NPCs: Hofrichter et al.^507^ mOrg: Chesnut et al.^508^; Hartmann et al.^509^	Isogenic and healthy controls	Kapr et al.^510^
AHDS	*SLC16A2*	1. Pd iPSC, Severe AHDS: *SLC16A2* (HEMI), p.A404fs416*^m^ 2. Pd iPSC, Severe AHDS: *SLC16A2* (HEMI), p.P321L^n^ 3. GE iPSC: *SLC16A2* (HEMI), KO^o^	Episomal plasmids (Fibroblasts)	Neurons: Ebert et al.^511^; Shelley et al.^512^ BMECs: Lippmann et al.^513^; Lippmann et al.^514^	Isogenic and healthy controls	Vatine et al.^515^
		1. Pd iPSC, Severe AHDS: *SLC16A2* (HEMI), p.A404fs416*^m^ 2. Pd iPSC, Severe AHDS: *SLC16A2* (HEMI), p.P321L^n^ 3. GE iPSC: *SLC16A2* (HEMI), KO^m^	Episomal plasmids (Fibroblasts)	OLs: Hu et al.^486^; Wang et al.^432^	Isogenic and healthy control	Vatine et al.^516^
		1. Pd iPSC, Severe AHDS: *SLC16A2* (HEMI), p.P321L^n^ 2. GE iPSC: *SLC16A2* (HEMI), KO^o^	Episomal vectors (Fibroblasts)	BMECs: Lippmann et al.^513^; Lippmann et al.^514^	Healthy controls	Braun et al.^517^
		1. Pd iPSC, UNK, *SLC16A2* (HEMI), p.A404fs416* 2. Pd iPSC, UNK, *SLC16A2* (HEMI), p.P321L	UNK (Fibroblasts)	cOrg: Lancaster et al.^447^	Unaffected carrier and isogenic control	Salas-Lucia et al.^518^
PCWH	*SOX10*	1. GE iPSC, *SOX10* (HMZ), KO 2. GE ESC, *SOX10* (HMZ), KO	UNK (Fibroblasts)	NCC: Hackland et al.^519^ Neurons: own protocol Schwann cell: own protocol	Unedited controls	Lai et al.^520^
DEVDFB	*U2AF2*	1. Pd iPSC, Severe: *U2AF2* (HET), p.R149W *2*. Pd iPSC, UNK: *U2AF2* (HET), p.R149W 3. Pd iPSC, Mild: *U2AF2* (HET), p.R150C 4. Pd iPSC, Mild: *U2AF2* (HET), p.K329del *5*. GE iPSC: *U2AF2* (HET), p.R149W 6. GE iPSC: *U2AF2* (HET), p.R150C	UNK (LCLs)	Neurons: Zhang et al.^448^	Healthy and unedited controls	Li et al.^521^
Bloc1s1-LD	*BLOC1S1*	1. Pd iPSC, UNK: *BLOC1S1* (cHET), c.206 A > C; c.359 G>A^p^	Sendai virus (PBMCs)	HLCs: Du et al.^522^	Isogenic control	Wu et al.^523^
SLS	*ALDH3A2*	1. Pd iPSC, Moderate severity: *ALDH3A2* (cHET), c.1339 A > G (p.K447E); c.57_132dup (p.I45Sfs*34) 2. Pd iPSC, Mild severity: *ALDH3A2* (HMZ), c.1339 A > G (p.K447E)	Episomal vectors (Fibroblasts, PBMCs)	Neurospheres: Matsumoto et al.^524^; Fujimori et al.^525^ Oligospheres: Numasawa-Kuroiwa et al.^187^	Healthy controls	Yamaguchi et al.^526^
CFD	*FOLR1*	1. Pd iPSC, UNK: *FOLR1* (UNK), UNK; + de novo variant in *CIC*, c.1057 C > T (p.R353*)	Sendai virus (Fibroblasts)	iPSCs	Healthy controls	Cao et al.^527^
*Myelin Vacuolization*						
Canavan	*ASPA*	1. Pd iPSC, UNK: *ASPA* (cHET), c.527 G > A (p.G176E); c.914 C > A (p.A305E)^q^ 2. Pd iPSC, UNK: *ASPA* (cHET), c.527 G > A (p.G176E); c.914 C > A (p.A305E) 3. Pd iPSC, UNK: *ASPA* (HMZ), c.854 A > C (p.E285A)^r^ 4. Pd iPSC, UNK: *ASPA* (HMZ), an A insertion in exon 2 5. Pd iPSC, UNK: *ASPA* (HMZ), c.731 A > G (p.H244R) 6. Pd iPSC, UNK: *ASPA* (cHET), c.382delC; c.502 C > T; c.693 C > T	Episomal (Fibroblasts)	NPCs: Liu et al.^478^ OLs: Douvaras & Fossati^104^; Li et al.^267^; Piao et al.^528^ ; Wang et al.^432^	NA	Feng et al.^257^
		1. Pd iPSC, UNK: *ASPA* (cHET), c.527 G > A (p.G176E); c.914 C > A (p.A305E)^q^	Virus (Fibroblasts)	NPCs: Liu et al.^478^	Healthy controls	Chao et al.^258^
		1. Pd iPSC, UNK: *ASPA* (cHET), c.527 G > A (p.G176E); c.914 C > A (p.A305E)^q^ 2. Pd iPSC, UNK: *ASPA* (HMZ), c.854 A > C (p.E285A)^r^	Episomal (Fibroblasts)	mOrgs: own protocol	Healthy controls	Feng et al.^259^
PKU	*PAH*	1. Control iPSCs exposed to phenyalanine	UNK (UNK)	cOrgs: Lancaster et al.^529^	Healthy controls	Kim et al.^530^
**Astrocytopathies**						
AxD	*GFAP*	1. Pd iPSC, Type I AxD: *GFAP* (HET), c.729 C > T (p.R239C) 2. Pd iPSC, Type I AxD: *GFAP* (HET), c.205 G > A (p.E63K) 3. Pd iPSC, Type II AxD: *GFAP* (HET), c.827 G > T (p.R276L)	Episomal (Fibroblasts, PBMCs)	Astrocytes: own protocol	Healthy controls	Kondo et al.^265^
		1. Pd iPSC, UNK: *GFAP* (HET), p.R239C 2. Pd iPSC, UNK: *GFAP* (HET), p.R79C 3. Pd iPSC, UNK: *GFAP* (HET), p.M73K	Episomal (Fibroblasts)	Astrocytes: own protocol, Krencik et al.^531^; Zhang et al.^532^ OLs: Douvaras & Fossati^104^	Healthy controls, isogenic control	Li et al.^267^
		1. Pd iPSC, Type II AxD: *GFAP* (HET), c.262 C > T (p.R88C)^s^	Viral (Fibroblasts)	Astrocytes: Chambers et al.^444^; Krencik et al.^531^	Isogenic control	Wang et al.^279^
		1. Pd iPSC, Type II AxD: *GFAP* (HET), c.262 C > T (p.R88C)^s^ 2. Pd iPSC, Type II AxD: *GFAP* (HET), c.1246 C > T (p.R416W)	Viral (Fibroblasts)	Astrocytes: Krencik et al.^531^	Isogenic control	Jones et al.^266^
		1. GE ESC: *GFAP* (HET), p.R239C	NR	Astrocytes: own protocol	Unedited ESCs	Canals et al.^271^
		1. Pd iPSC, Type I AxD: *GFAP* (HET), c.715 C.T (p.R239C)^t^	Sendai virus (Fibroblasts)	Astrocytes: StemCell Technologies Kit	Isogenic control	Battaglia et al.^268^
		1. GE ESC: *GFAP* (HET), p.R239C	NR	Astrocytes: Canals et al.^271^	Unedited ESCs	Gao et al.^272^
		1. Pd iPSC, Type II AxD: *GFAP* (HET), c.262 C > T (p.R88C)^s^	Viral (Fibroblasts)	Astrocytes: Krencik et al.^531^	Isogenic control	Wang et al.^278^
		1. Pd iPSC, Type I AxD: *GFAP* (HET), c.715 C.T (p.R239C)^t^	Sendai virus (Fibroblasts)	Astrocytes: Canals et al.^271^ Neurons: Zhang et al.^448^ cOrg: Ormel et al.^533^; Lancaster et al.^534^; Verkerke et al.^535^; Yoon et al.^536^	Isogenic control	Matusova et al. ^270^
		1. Pd iPSC, UNK: *GFAP* (cHET), c.791_792TG > CT	Episomal vector (PBMCs)	Astrocytes: own protocol	Healthy controls	Nonaka et al.^269^
		1. Pd iPSC, Infantile AxD: *GFAP* (HET), c.262 C > T (p.R88C) 2. Pd iPSC, Infantile AxD: *GFAP* (HET), c.715 C > T (p.R239C) 3. Pd iPSC, Infantile AxD: *GFAP* (HET), c.230 A > G (p.N77S)	mRNA (Fibroblasts)	Astrocytes: Canals et al.^271^	Healthy controls	Yi et al.^273^
VWM	*EIF2B1, EIF2B2*, *EIF2B3, EIF2B4, EIF2B5*	1. Pd iPSC, UNK: *EIF2B5* (HMZ), c.1484 A > G 2. Pd iPSC, UNK: *EIF2B5* (HMZ), c.806 G > A	Lentiviral (Fibroblasts)	Astrocytes: Nadadhur et al.^537^, own protocol	Healthy controls	Leferink et al.^292^
		1. Pd iPSC, Early childhood-onset VWM: *EIF2B5* (cHET), c.1827_1838del (p.S610_D613del); c.1157 G > A (p.G386V)^u^ 2. Pd iPSC, Early childhood-onset VWM: *EIF2B3* (cHET), c.140 G > A (p.G47E); c.1037 T > C (p.I346T)^v^	Episomal (Fibroblasts)	Neurons, astrocytes, OLs: own protocol	Healthy controls	Zhou et al.^289^
		1. Pd iPSC, Early childhood-onset VWM: *EIF2B5* (cHET), p.R113H; p.A403V^w^ 2. Pd iPSC, Early childhood-onset VWM: *EIF2B2* (cHET), p.G200V; p.E213G^x^	mRNA (Fibroblasts)	Astrocytes: Denham et al.^538^	Unaffected carriers and healthy controls	Ng et al.^305^
		1. Pd iPSC, Early childhood-onset VWM: *EIF2B5* (cHET), c.1827_1838del (p.S610_D613del); c.1157 G > A (p.G386V)^u^ 2. Pd iPSC, Early childhood-onset VWM: *EIF2B3* (cHET), c.140 G > A (p.G47E); c.1037 T > C (p.I346T)^v^	Episomal (Fibroblasts)	Astrocytes, OLs: own protocol	Healthy controls	Deng et al.^290^
		1. Pd iPSC, Early childhood-onset VWM: *EIF2B5* (cHET), c.1827_1838del (p.S610_D613del); c.1157 G > A (p.G386V)^u^ 2. Pd iPSC, Early childhood-onset VWM: *EIF2B4* (cHET), c.932 T > C (p.I311T); c.1195 A > C (p.K399Q)	Episomal (Fibroblasts)	cOrg: Lancaster et al.^447^	Healthy control	Deng et al.^293^
		1. Pd iPSC, Early childhood-onset VWM: *EIF2B5* (cHET), p.R113H; p.A403V^w^ 2. Pd iPSC, Early childhood-onset VWM: *EIF2B2* (cHET), p.G200V; p.E213G^x^	Overexpression (Fibroblasts)	Astrocytes: Canals et al.^271^, own protocol	Healthy controls	Ng et al.^291^
AGS	*TREX1, RNASEH2B, IFIH1, ADAR1, LSM11, RNU7-1*	1. GE ESC: *TREX1* (HMZ), p.V63fs 2. GE ESC: *TREX1* (HMZ), p.E83fs 3. Pd iPSC, Early-onset AGS: *TREX1* (HMZ), p.V201D	Episomal (Fibroblasts)	NPCs: Griesi-Oliveira et al.^495^ Neurons: Marchetto et al.^494^; Chailangkarn et al.^539^ Astrocytes: Chailangkarn et al.^539^ cOrg: Pasca et al.^540^	Unedited ESCs, Isogenic control	Thomas et al.^325^
		1. Pd iPSC, Type 1 AGS: *TREX1* (cHET), c.260insAG (p.S88fs*22); c.290 G > A (p.R97H) 2. Pd iPSC, Type 2 AGS: *RNASEH2B* (HMZ), c.529 G > A (p.A177T) 3. Pd iPSC, Type 7 AGS: *IFIH1* (HET), c.2471 G > A	Sendai virus (Fibroblasts)	iPSCs	Healthy control	Genova et al.^326^
		1. GE iPSC: *TREX1* (HMZ), KO 2. GE iPSC: *RNASEH2B* (HMZ), KO *3*. Pd iPSC, AGS1: *TREX1* (cHET), p.R97H; p.S88fs*22 4. Pd iPSC, AGS2: *RNASEH2B* (HMZ), p.A177T	Sendai virus (Fibroblasts)	NPCs: Chambers et al.^444^ Neurons: Frati et al.^96^ Astrocytes: Santos et al.^541^	Healthy control	Giordano et al.^327^
		1. GE ESC: *TREX1* (HMZ), p.V63fs 2. GE ESC: *TREX1* (HMZ), p.E83fs	NR	MGL: Mesci et al.^542^ cOrg: Marton et al.^543^	Unedited ESCs	Goldberg et al.^328^
**Leuko-axonopathies**						
TUBB4A-LD	*TUBB4A*	1. Pd iPSC, DYT: *TUBB4A* (HET), p.R2G 2. GE iPSC: *TUBB4A* (HET), c.444_448del 3. GE iPSC: *TUBB4A* (HET), p.R2G 4. GE iPSC: *TUBB4A* (HET), p.D249N 5. GE iPSC: *TUBB4A* (HET), p.A271T	UNK (Fibroblasts)	Neurons: Shi et al.^445^	Healthy controls	Vulinovic et al.^345^
LBSL	*DARS2*	1. Pd iPSC, Early-infantile onset LBSL: *DARS2* (cHET), c.753 G > T (p.M251I); c.228-20delTTinsC (p.R76Sfs*5) 2. Pd iPSC, Early-infantile onset LBSL: *DARS2* (cHET), c.473 A > T (p.E158V); c.829 G > A (p.E277K) 3. Pd iPSC, Childhood onset LBSL: *DARS2* (cHET), c.265 C > T (p.L89F); c.492+2 T > C (p.M134_K165del) 4. Pd iPSC, Childhood onset LBSL: *DARS2* (cHET), c.228-20delTTinsC (p.R76Sfs*5); ? 5. Pd iPSC, Early-infantile onset LBSL: *DARS2* (cHET), c.228-20delTTinsC (p.R76Sfs*5); c.492+2 T > C (p.M134_K165del) 6. Pd iPSC, Early-infantile onset LBSL: *DARS2* (cHET), c.228-17 C > G; c.492+2 T > C (p.M134_K165del) 7. Pd iPSC, Childhood onset LBSL: *DARS2* (cHET), c.228-17 C > G; c.492+2 T > C (p.M134_K165del)	Episomal (PBMCs)	cOrg: STEMCELL Technologies kit Neurons: Ngn2 lentivirus	Healthy controls	Guang et al.^354^
GM1	*GLB1*	1. Pd iPSC, UKN: *GLB1* (HMZ), p.R201C	Retroviral (Fibroblasts)	NPCs: Son et al.^544^; Son et al.^545^	Healthy controls	Son et al.^546^
		1. GE iPSC: *GLB1* (HMZ), KO	Episomal (Fibroblasts)	cOrg: Lancaster et al.^447^	Unedited controls	Latour et al.^547^
		1. Pd iPSC, Juvenile onset GM1: *GLB1* (HMZ), c.601 C > T (p.R201C) 2. Pd iPSC, Juvenile onset GM1: *GLB1* (HMZ), c.601 C > T (p.R201C) 3. Pd iPSC, Adult onset GM1: *GLB1* (HMZ), c.152 T > C (p.I51T)	Sendai virus (Fibroblasts)	NPCs: Yan et al.^548^ Neurons: Yan et al.^548^	Healthy controls	Kajihara et al.^549^
GM2	*HEXA, HEXB, GM2A*	1. Pd iPSC, Infantile onset GM2: *HEXB* (cHET), 16 kB; c.IVS10-2A > G	Episomal (Fibroblasts)	cOrg: Lancaster et al.^534^; Lancaster et al.^447^	Isogenic and healthy controls	Allende et al.^550^
		1. Pd iPSC, UNK: *HEXA* (HMZ), c.1278insTATC 2. Pd iPSC, UNK: *HEXA* (cHET), c.1278insTATC; p.Trp392*	Sendai virus (Fibroblasts)	NPCs: ThermoFisher Kit Neurons: STEMdiff Kit	Healthy controls	Vu et al.^551^
		1. Pd iPSC, UNK: *HEXA* (HMZ), c.IVS5-1G > T 2. Pd iPSC, UNK: *HEXA* (HMZ), c.IVS5-1G > T	Sendai virus (Fibroblasts)	NPCs: own protocol Neurons: own protocol	Healthy controls	Matsushita et al.^552^
GAN	*KLHL16*	1. Pd iPSC, UNK: *KLHL16* (cHET), p.S52G; p.C393* 2. Pd iPSC, UNK: *KLHL16* (HMZ), p.A94E 3. Pd iPSC, UNK: *KLHL16* (UNK), UNK	Retroviral (Fibroblasts)	MN: Boulting et al.^553^	Healthy controls	Johnson-Kerner et al.^554^
		1. Pd iPSC, UNK: *KLHL16* (cHET), p.R574C; p.G280del 2. Pd iPSC, UNK: *KLHL16* (HMZ), p.Y89S 3. Pd iPSC, UNK: *KLHL16* (HMZ), p.R138L 4. Pd iPSC, UNK: *KLHL16* (HMZ), p.IVS4 + 1 G > A 5. Pd iPSC, UNK: *KLHL16* (HMZ), deletion of exons 10 and 11 6. Pd iPSC, UNK: *KLHL16* (cHET), p.E486K; 3.2 kb deletion 7. Pd iPSC, UNK: *KLHL16* (HMZ), p.G332R	Sendai virus (Fibroblasts)	Astrocytes: Battaglia et al.^268^ cOrg: Pasca et al.^540^ NPCs: StemCell Technologies Kit	Isogenic and healthy controls	Battaglia et al.^555^
MPS	*IDUA, IDS, SGSH, NAGLU, HGSNAT, GNS, GUSB*	1. Pd iPSC, MPS Type I (Hurler): *IDUA* (cHET), p.Y167*; p.W402* 2. Pd iPSC, MPS Type I (Hurler): *IDUA* (HMZ), p.W402*	Retroviral (Keratinocytes, Bone marrow MSCs)	HC: own protocol	Isogenic and healthy controls	Tolar et al.^556^
		1. Pd iPSC, MPS Type III B (Sanfilippo): *NAGLU* (HMZ), c.531+1 G > C 2. Pd iPSC, MPS Type III B (Sanfilippo): *NAGLU* (HMZ), p.R482W	Retroviral (Fibroblasts)	NPCs/Neurons: Ebert et al.^557^	Healthy controls	Lemonnier et al.^558^
		1. Pd iPSC, MPS type III B (Sanfilippo): *NAGLU* (UNK), UNK 2. Pd iPSC, MPS type III B (Sanfilippo): *NAGLU* (UNK), UNK	Retroviral (Fibroblasts)	NPCs: Brennand et al.^559^; Kim et al.^459^	Healthy controls	Bruyere et al.^560^
		1. Pd iPSC, MPS type III C (Sanfilippo): *HGSNAT* (cHET), c.633+1 G > A; c.1334 T > C (p.L445P)^y^ 2. Pd iPSC, MPS type III C (Sanfilippo): *HGSNAT* (HMZ), c.372-2 A > G	Retroviral (Fibroblasts)	NPCs/Neurons: Cho et al.^460^	Healthy control	Canals et al.^561^
		1. Pd iPSC, MPS type VII (Sly): *GUSB* (UNK), UNK	Retroviral (Fibroblasts)	NPCs: Koch et al.^469^	Isogenic and healthy controls (check)	Griffin et al.^471^
		1. Pd iPSC, MPS type VII (Sly): *GUSB* (HMZ), p.L176F	Retroviral (Fibroblasts)	NPCs: Chambers et al.^444^ Neurons: Simão et al.^562^	Healthy controls	Bayo-Puxan et al.^563^
		1. Pd iPSC, MPS type I (Scheie): *IDUA* (UNK), UNK 2. Pd iPSC, MPS type I (Hurler Scheie): *IDUA* (UNK), UNK 3. Pd iPSC, MPS type I (Hurler): *IDUA* (cHET) c.IVS5AS-7G > A; p.W402*	Sendai virus (Fibroblasts)	NPCs: Thermo Fischer Neural Induction Kit	Healthy controls	Swaroop et al.^564^
		1. Pd iPSC, MPS type II (Hunter): *IDS* (HEMI), c.85 C > T (p.Q29*) 2. Pd iPSC, MPS type II (Hunter): *IDS* (HEMI), c. 85 C > T (p.Q29*) 3. Pd iPSC, MPS type II (Hunter): *IDS* (HEMI), c.182 C > T (p.S61F)	Lentiviral (PBMCs)	NPCs: Chambers et al.^444^ Neurons: Shi et al.^445^	Healthy control, unaffected carriers	Kobolak et al.^565^
		1. Pd iPSC, MPS type I (Hurler): *IDUA* (cHET), c.1073_1093del (p.H358_T364del); c.1205 G > A (p.W402*)^z^	Sendai virus (Fibroblasts)	NPC: Chambers et al.^444^ Neurons: Own protocol	Healthy controls	Lito et al.^566^
		1. Pd iPSC, MPS type III C (Sanfilippo): *HGSNAT* (cHET), c.633+1 G > A; c.1334 T > C (p.L445P)^t^ 2. GE iPSC: *HGSNAT* (cHET), c.195_210del (p.N66Gfs*15); c.207_208insCA (p.Y70Hfs*17)^ab^ 3. GE iPSC: *HGSNAT* (cHET), c.195_210del (p.N66Gfs*15); c.209delinsGAATG (p.Y70*)^ac^	Retrovirus (Fibroblasts)	Neurons: Zhang et al.^448^ Astrocytes: Canals et al.^271^	Healthy control	Beneto et al.^567^
		1. Pd iPSC, MPS type III B (Sanfilippo): *NAGLU* (HMZ), c.457 G > A (p.E153K)^ad^ 2. Pd iPSC, MPS type III B (Sanfilippo): *NAGLU* (HMZ), c.1876C>T (p.R626*)	Sendai virus (Fibroblasts)	NPCs: STEMdiff kit Neurons: Vu et al.^551^	Healthy controls	Huang et al.^568^
		1. Pd iPSC, MPS type III A (Sanfilippo): *SGSH* (cHET), c.892 T > C (p.S298P); c.1298 G > A (p.R433Q) 2. Pd iPSC, MPS type III A (Sanfilippo): *SGSH* (HMZ), c.672 C > A (p.Y224*)	Sendai virus (Fibroblasts)	NPCs: Shi et al.^445^; Homan et al.^569^ Neurons: Shi et al.^445^	Healthy controls	Lehmann et al.^570^
		1. Pd iPSC, MPS type II (Hunter): *IDS* (HEMI), c.208insC (p.H70Pfs*29)^ae^ 2. Pd iPSC, MPS type II (Hunter): *IDS* (HEMI), UNK 3. Pd iPSC, MPS type II (Hunter): *IDS* (HEMI), UNK	Sendai virus (Fibroblasts)	Neurons: STEMdiff kit	Healthy controls	Hong et al.^571^
		1. Pd iPSC, MPS type II (Hunter): *IDS* (HEMI), c.1402 C > T (p.R468W) 2. Pd iPSC, MPS type II (Hunter): *IDS* (HEMI), c.1214_1215delCT 3. Pd iPSC, MPS type II (Hunter): *IDS* (HEMI), c.IVS7+5 G > C 4. Pd iPSC, MPS type II (Hunter): *IDS* (HEMI), c.1122 C > T (p.G374G)	Sendai virus (PBMCs)	Neurons: Shi et al.^445^	Healthy and isogenic controls	Chen et al.^572^
NCL	*CLN1 – CLN13*	1. Pd iPSC, Late-infantile NCL: *TPP1* (cHET), c.509-1 G > C; c.622 C > T (p.R208*) 2. Pd iPSC, Late-infantile NCL: *TPP1* (cHET), c.509-1 G > C; c.622 C > T (p.R208*) 3. Pd iPSC, Juvenile NCL: *CLN3* (HMZ), 1.02 kb deletion 4. Pd iPSC, Juvenile NCL: *CLN3* (cHET), c.1056+3 A > G; c.1247 A > G (p.D416G) 5. Pd iPSC, Juvenile NCL: *CLN3* (HMZ), 1.02 kb deletion 6. Pd iPSC, Juvenile NCL: *CLN3* (HMZ), 1.02 kb deletion	Retroviral (Fibroblasts)	NPCs: own protocol Neurons: own protocol	Healthy controls	Lojewski et al.^573^
		1. Pd iPSC, Juvenile NCL: *CLN3* (HMZ), 1.02 kb deletion 2. Pd iPSC, Juvenile NCL: *CLN3* (cHET), 1.02 kb deletion; p.L101del3CTC	Sendai virus (Fibroblasts)	iPSCs	NR	Burnight et al.^574^
		1. Pd iPSC, Infantile NCL: *PPT1* (cHET), c.739 T > C (p.Y247H); c.3 G > A (p.M1I) 2. Pd, iPSC, Late-infantile NCL: *TPP1* (cHET), c.379 C > T (p.R127*); c.622 C > T (p.R208*) 3. Pd, iPSC, Late-infantile NCL: *TPP1* (cHET), c.380 G > A (p.R127Q); c.IVS5-1G > C	Sendai virus (Fibroblasts)	NPCs: Thermo Fisher Neural Induction Kit	Healthy controls	Sima et al.^575^
		1. GE iPSC: *CLN3* (HMZ), c.1054 C > T (p.Q352*)^af^	UNK (UNK)	EC: Prasain et al.^576^ cOrg: Lancaster et al.^447^	Unedited controls	Gomez-Giro et al.^577^
		1. Pd iPSC, Juvenile NCL: *CLN3* (cHET), c.988 G > T (p.V330F); 1 kb deletion	UNK (UNK)	Neurons: Yan et al.^548^; Efthymiou et al.^578^ BMECs: Lippmann et al.^513^; Patel et al.^579^	Healthy control	Kinarivala et al.^580^
		1. Pd iPSC, NCL7: *MFSD8* (HMZ), c.1393 C > T (p.R465W)	UNK (Fibroblasts)	NPCs: Fitzpatrick et al.^581^	Healthy control	Soldati et al.^582^
		1. Pd iPSC, Juvenile NCL: *CLN3* (cHET), p.E295K; 966 bp deletion	Episomal vectors (Fibroblasts)	Neurons: Talbot et al.^583^	Healthy and isogenic control	Chear et al.^584^
		1. GE ESC: *CLN3* (HMZ), Δ7–8	NR	RPE: Tang et al.^585^; Galloway et al.^586^; Manian et al.^587^; Galloway et al.^588^	Unedited control	Han et al.^589^
		1. GE iPSC: *CLN3* (HMZ), c.1054 C > T (p.Q352*)^af^	UNK (UNK)	cOrg: Lancaster et al.^447^	Unedited controls	Heins-Marroquin et al.^590^
ZSD	*PEX1, PEX2, PEX3, PEX5, PEX6, PEX10, PEX11B, PEX12, P2X13, PEX14, PEX16, PEX19, PEX26*	1. Pd iPSC, UNK: *PEX1* (cHET), c.2097_2098insT (p.I700fs); c.2916delA (p.G973fs) 2. Pd iPSC, UNK: *PEX1* (cHET), c.2097_2098insT (p.I700fs); c.2916delA (p.G973fs) 3. Pd iPSC, UNK: *PEX1* (HMZ), c.2528 G > A (p.G843D) 4. Pd iPSC, UNK: *PEX1* (HMZ), c.2528 G > A (p.G843D) 5. Pd iPSC, UNK: *PEX10* (cHET), c.337delC (p.L113fs), c.890 T > C (p.L297P) 6. Pd iPSC, UNK: *PEX12* (HMZ), c.959 C > T (p.S320F) 7. Pd iPSC, UNK: *PEX26* (HMZ), c.292 C > T (p.R98W)	Retrovirus (Fibroblasts)	MN: Hu et al.^486^;^616^NCL: Heins-Marroquin^590^ Xia et al.^591^ OL: Nistor et al.^592^; Zhang et al.^593^; Izrael et al.^485^; Hatch et al.^594^; Sharp et al.^595^ HLC: Duan et al.^596^; Duan et al.^597^	Healthy controls	Wang et al.^598^
**Microgliopathies**						
PLOSL	*TREM2, TRYOBP*	1. Pd iPSC, Late neurologic stage: *TREM2* (HMZ), p.T66M 2. Pd iPSC, Early neurologic stage: *TREM2* (HMZ), p.W50C	Sendai virus (Fibroblasts)	MGL: van Wilgenburg et al.^446^; Karlsson et al.^599^	Healthy controls	Brownjohn et al.^367^
		1. Pd iPSC, Late neurologic stage: *TREM2* (HMZ), p.T66M 2. Pd iPSC, Early neurologic stage: *TREM2* (HMZ), p.W50C	Sendai virus (Fibroblasts)	MGL: van Wilgenburg et al.^446^	Healthy controls	Garcia-Reitboeck et al.^368^
		1. Pd iPSC, Late neurologic stage: *TREM2* (HMZ), p.Q33* 2. Pd iPSC, Early neurologic stage: *TREM2* (HMZ), p.Q33* 3. Pd iPSC, UNK: *TREM2* (HMZ), p.Q33*	Sendai virus (Fibroblasts)	MGL: Abud et al.^391^; McQuade et al.^390^	Healthy controls	Filipello et al.^369^
CSF1R-LD	*CSF1R*	1. Pd iPSC, late-onset CSF1R-LD: *CSF1R* (HET), c.2350 G > A (p.V784M)	Episomal (PBMCs)	MGL: own protocol	Healthy controls	Dorion et al.^387^
		1. Pd iPSC, UNK: *CSF1R* (HET), p.L786S	Sendai virus (Fibroblasts)	MGL: Abud et al.^391^; McQuade et al.^390^	Isogenic control	Chadarevian et al.^388^
		1. GE iPSC: *CSF1R* (HET), p.I794T	Episomal (UNK)	MGL: Haenseler et al.^480^	Unedited controls	Larson et al.^389^
**Leuko-vasculopathies**						
CADASIL	*NOTCH3*	1. Pd iPSC, UNK: *NOTCH3* (HET), c.3226 C > T (p.R1076C)	Episomal (Fibroblasts)	VSMC: Patsch et al.^600^ EC: own protocol	Healthy controls	Ling et al.^406^
		1. Pd iPSC, UNK: *NOTCH3* (HET), p.R153C^ag^ 2. Pd iPSC, UNK: *NOTCH3* (HET), p.C224Y^ah^	Sendai virus (Fibroblasts)	MC: Cheung et al.^601^ EC: own protocol	Healthy controls and unaffected carriers	Kelleher et al.^408^
		1. Pd iPSC, UNK: *NOTCH3* (HET), p.R182C 2. Pd iPSC, UNK: *NOTCH3* (HET), p.R141C 3. Pd iPSC, UNK: *NOTCH3* (HET), p.C106R	Retroviral or episomal (Fibroblasts, PBMCs)	MC: Taura et al.^602^; Tatsumi et al.^603^	Healthy controls	Yamamoto et al.^407^
		1. Pd iPSC, UNK: *NOTCH3* (HET), p.R153C^ag^ 2. Pd iPSC, UNK: *NOTCH3* (HET), p.C224Y^ah^	Sendai virus (Fibroblasts)	BMEC: Lippmann et al.^513,514^ Neurons: Shi et al.^445^ Astrocytes: STEMdiff Astrocyte Kit	Healthy controls	Zhang et al.^418^
		1. Pd iPSC, UNK: *NOTCH3* (HET), c.1261 C > T (p.R421C)	Sendai virus (PBMCs)	VSMC: Patsch et al.^600^ BVO: Wimmer et al.^604^	Isogenic control	Wang et al. ^402^
		1. GE iPSC: *NOTCH3* (HET), p.R153C 2. GE iPSC: *NOTCH3* (HMZ), p.R153C 3. GE iPSC: *NOTCH3* (HET), p.R182C	UNK (UNK)	BVO: Wimmer et al.^604^	Unedited controls	Ahn et al.^403^
		1. Pd iPSC, UNK: *NOTCH3* (HET), c.421C>T (p.R141C)	Sendai virus (PBMCs)	BVO: Zhao et al.^605^	Healthy controls	Wang et al.^412^
		1. Pd iPSC, UNK: *NOTCH3* (HET), p.R90C 2. Pd iPSC, UNK: *NOTCH3* (HET), p.R182C 3. Pd iPSC, UNK: *NOTCH3* (HET), p.R1242C 4. Pd iPSC, UNK: *NOTCH3* (HET), p.C591R	Sendai virus (PBMCs)	iPSCs	Healthy controls	Bugallo-Casal et al.^404^
CARASIL	*HTRA1*	1. Pd iPSC, HTRA-CSVD (mild form): *HTRA1* (HET), c.905 G > A (p.R302Q)	Episomal vectors (PBMCs)	iPSCs	Healthy controls	Qian et al. ^606^
LCC	*SNORD118*	1. GE iPSC: *SNORD118* (HET), KO 2. GE iPSC: *SNORD118* (HET), KO 3. GE iPSC: *SNORD118* (HET), KO	UNK (UNK)	MSCs: Faal et al.^607^; Stebbins et al.^608^; Bajpai et al.^609^	Unedited controls	Jariyasakulroj et al.^426^
		1. GE iPSC: *SNORD118* (HET), KO 2. GE iPSC: *SNORD118* (HET), KO 3. GE iPSC: *SNORD118* (HET), KO 4. GE iPSC: *SNORD118* (HMZ), c.*5 C > G 5. GE iPSC: *SNORD118* (HMZ), c.*5 C > G 6. GE iPSC: *SNORD118* (HMZ), c.*5 C > G	UNK (UNK)	cOrg: Zhang et al.^610^; Zhang et al.^611^	Unedited controls	Zhang et al. ^425^
		1. GE iPSC: *SNORD118* (HMZ), c.*5 C > G 2. GE iPSC: *SNORD118* (HMZ), c.*5 C > G 3. GE iPSC: *SNORD118* (HMZ), c.*5 C > G	UNK (UNK)	NPC: UNK NCC: UNK cOrg: UNK ncOrg: UNK	Unedited controls	Zhang et al. ^425^
COL4A-LD	*COL4A1* *COL4A2*	1. Pd iPSC, COL4A1-LD: *COL4A1* (HET), c.2263 G > A (p.G755R) 2. Pd iPSC, Asymptomatic: *COL4A2* (HET), p.G702D	Sendai virus (UNK)	MC: Cheung et al.^601^; Serrano et al.^612^ BMECs: Hollmann et al.^613^	Healthy and isogenic controls	Al-Thani et al.^614^
CMRCC	*CTC1*	1. Pd iPSC, CMRCC: *CTC1* (cHET), c.833 G > T (p.G278V); c.841 T > C (p.Y281H)	Sendai virus (PBMCs)	iPSCs MSCs: own protocol	Healthy controls	Oudrhiri et al.^615^

Genetic information is written as was published in the original article.

*UNK* unknown, *NR* not reported, *NA* not applicable, *HMZ* homozygous, *cHET* compound heterozygous, *HEMI* hemizygous, *iPSC* induced pluripotent stem cells, *ESC* embryonic stem cells, *GE* genetically engineered, *OL* oligodendrocyte, *cOrg* cerebral organoids, *BMEC* brain microvascular endothelial cells, *NPC* neural progenitor cells, *OPC* oligodendrocyte progenitor cells, *MNPs* motor neuron progenitor cells, *MN* motor neurons, *MGL* microglia, *mOrg* myelinating organoid, *GPCs* glial progenitor cells, *NCC* neural crest cells, *MSC* mesenchymal stem cells, *VSMC* vascular smooth muscle cells, *MC* mural cells, *EC* endothelial cells, *PBMC* peripheral blood mononuclear cell, *ncOrg* neural crest organoid, *BVO* blood vessel organoid, *RPE* retinal pigment epithelial, *HC* hematopoietic cells, *HLC* hepatic-like cells, *X-ALD* adrenoleukodystrophy, *MLD* metachromatic leukodystrophy, *MSD* Multiple Sulfatase Deficiency, *CTX* Cerebrotendinous xanthomatosis, *SLS* Sjögren–Larsson syndrome, *PMD* Pelizaeus-Merzbacher disease, *POLR3-HLD* POLR3-related leukodystrophy, *ODDD* oculodentodigital dysplasia, *AHDS* Allan-Herndon-Dudley syndrome, *PCWH* peripheral demyelinating neuropathy, central dysmyelination, Waardenburg syndrome, and Hirschsprung disease, *DEVDFB* Developmental delay, dysmorphic facies, and brain anomalies, *Bloc1s1-LD* Bloc1s1-related leukodystrophy, *CFD* cerebral folate deficiency, *PKU* phenylketonuria, *AxD* Alexander disease, *VWM* Vanishing White Matter, *AGS* Aicardi-Goutières syndrome, *TUBB4A-LD* TUBB4A-related leukodystrophy, *LBSL* leukoencephalopathy with brainstem and spinal cord involvement and lactate elevation, *GM1* GM1 gangliosidosis, *GM2* GM2 gangliosidosis, *GAN* giant axonal neuropathy, *MPS* mucopolysaccharidoses, *NCL* neuronal ceroid lipofuscinoses, *ZSD* Zellweger Spectrum Disorder, *PLOSL* polycystic lipomembranous osteodysplasia with sclerosing leukoencephalopathy, *CSF1R-LD* CSF1R-related leukoencephalopathy, *CADASIL* cerebral autosomal dominant arteriopathy with subcortical infarcts and leukoencephalopathy, *CARASIL* cerebral autosomal recessive arteriopathy with subcortical infracts and leukoencephalopathy, *LCC* leukoencephalopathy with brain calcifications and cysts, *COL4A-LD* COL4A-related disorders, *CMRCC* cerebroretinal microangiopathy with calcifications and cysts, *HTRA1-CSVD* HTRA1 cerebral small vessel disease (refers to the milder phenotype), *COFS* cerebral-oculo-fascio-skeletal syndrome, *DYT* spasmodic dysphonia.

^a-ah^Letters indicate if the iPSCs utilized in different studies are derived from the same individual.

The most frequently published leukodystrophies were mucopolysaccharidoses (MPS) (15.46%, n = 30), adrenoleukodystrophy (X-ALD) (9.278%, n = 18), Cerebral Autosomal Dominant Arteriopathy with Subcortical Infarcts and Leukoencephalopathy (CADASIL) (5.670%, n = 11), Neuronal Ceroid Lipofuscinosis (NCL) (5.670%, n = 11), Aicardi-Goutières Syndrome (AGS) (5.155%, n = 10), Alexander Disease (AxD) (4.639%, n = 9), and Pelizaeus-Merzbacher Disease (PMD) (4.124%, n = 8). Specifically, for manuscripts involving disease modeling (e.g. excluding reports of generation of iPSCs), the most commonly represented disorders were MPS (11.11%, n = 14), X-ALD (7.936%, n = 10), AxD (7.143%, n = 9), NCL (7.143%, n = 9), CADASIL (6.349%, n = 8), Vanishing White Matter (VWM) (4.762%, n = 6), and Krabbe (4.762%, n = 6) (Fig. 2). These disorders are among the more prevalent and longest-known leukodystrophies, and their increased representation in iPSC studies likely reflects their higher incidence, greater availability of patient-derived samples, and the longer history of clinical and genetic characterization, which has facilitated research efforts. Of all the included searched leukodystrophies (n = 108), only 41 were identified using the search criteria. Among these, iPSC models have been published for 35 different leukodystrophies, while an additional 6 leukodystrophies have reports describing the generation of patient-specific iPSC lines without any accompanying disease modeling or phenotypic characterization. The majority of leukodystrophies currently have no published iPSC models.

**Fig. 4:**
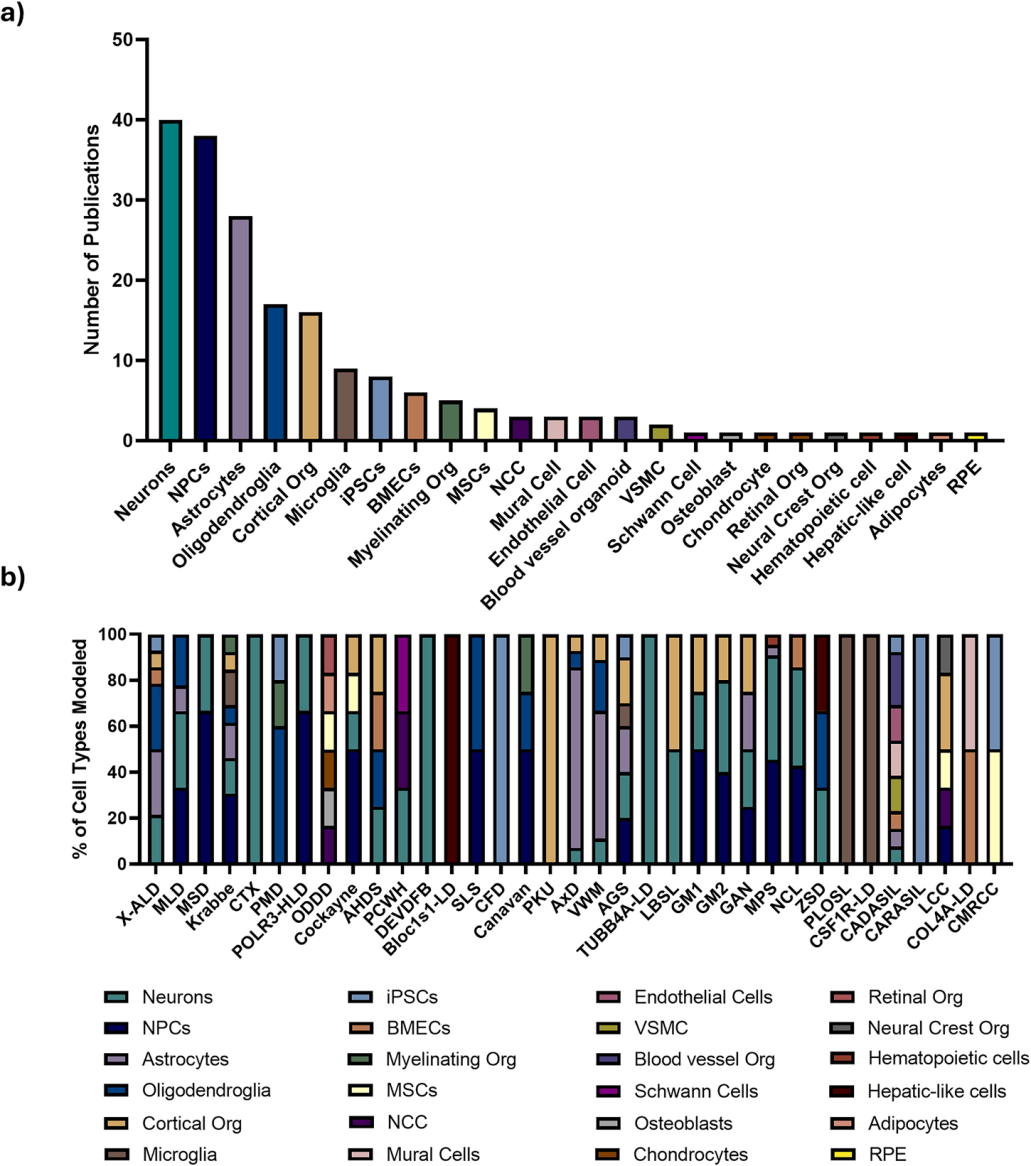
Diversity and frequency of cell types modeled in leukodystrophy research. **a** Total number of publications that modeled each cell type across all leukodystrophy studies.**b** Distribution of modeled cell types across individual leukodystrophies. For each leukodystrophy, bars show the relative proportion of modeled cell types, calculated as the percentage of total cell types studied for that disease. Figure created using GraphPad Prism Version 10.5.0. NPCs neural progenitor cells, Org Organoid, iPSCs induced pluripotent stem cells, BMECs brain microvascular endothelial cells, MSCs mesenchymal stem cells, NCC neural crest cell, VSMC vascular smooth muscle cells, RPE retinal pigment epithelial cells, X-ALD adrenoleukodystrophy, MLD metachromatic leukodystrophy, MSD multiple sulfatase deficiency, CTX cerebrotendinous xanthomatosis, SLS Sjögren–Larsson syndrome, PMD Pelizaeus-Merzbacher disease, POLR3-HLD POLR3-related leukodystrophy, ODDD oculodentodigital dysplasia, AHDS Allan-Herndon-Dudley syndrome, PCWH peripheral demyelinating neuropathy, central dysmyelination, Waardenburg syndrome, and Hirschsprung disease, DEVDFB developmental delay, dysmorphic facies, and brain anomalies, Bloc1s1-LD Bloc1s1-related leukodystrophy, CFD cerebral folate deficiency, PKU Phenylketonuria, AxD Alexander disease, VWM vanishing white matter, AGS Aicardi-Goutières syndrome, TUBB4A-LD TUBB4A-related leukodystrophy, LBSL leukoencephalopathy with brainstem and spinal cord involvement and lactate elevation, GM1 GM1 gangliosidosis, GM2 GM2 gangliosidosis, GAN giant axonal neuropathy, MPS mucopolysaccharidoses, NCL neuronal ceroid lipofuscinoses, ZSD Zellweger Spectrum Disorder, PLOSL polycystic lipomembranous osteodysplasia with sclerosing leukoencephalopathy, CSF1R-LD CSF1R-related leukoencephalopathy, CADASIL cerebral autosomal dominant arteriopathy with subcortical infarcts and leukoencephalopathy, CARASIL cerebral autosomal recessive arteriopathy with subcortical infracts and leukoencephalopathy, LCC leukoencephalopathy with brain calcifications and cysts, COL4A-LD COL4A-related disorders, CMRCC cerebroretinal microangiopathy with calcifications and cysts.

The differentiated cell types of focus varied among the studies, with a total of 24 different iPSC cell types/models. Most articles focused on differentiation to neurons (31.75%, n = 40), followed by neural progenitor cell (NPC) (30.16%, n = 38), astrocytes (22.22%, n = 28), oligodendrocytes or their progenitors (13.49%, n = 17), cerebral organoids (cOrg) (12.70%, n = 16), and microglia (7.143%, n = 9), among others (Fig. 4). 2D differentiation protocols (78.57%, n = 99) were more widely used compared to 3D approaches (22.22%, n = 28), however, since 2022 more publications have been adopting 3D technologies. Lastly, a majority of studies utilized growth-factor based differentiation protocols (84.92%, n = 107) compared to directed differentiation via overexpression (9.524%, n = 12).

### Insights from stem cell modeling across leukodystrophies

Since their development just over a decade ago, iPSC-based models have launched a new era in modeling leukodystrophies. For long-established leukodystrophies, stem cells offer the opportunity to study well-known disease mechanisms in human-specific cells. Additionally, they provide crucial insights into newly identified, ultra-rare leukodystrophies. While stem cell disease models for lysosomal storage disorders, including mucopolysaccharidosis and neuronal ceroid lipofuscinosis, and some astrocytopathies have been extensively reviewed^29–39^, we elected to exclude those and focus on leukodystrophies for which there were significant recent updates.

### Myelin disorders

Myelin disorders are a distinct category within the leukodystrophy classification system, characterized by primary dysfunction of oligodendrocytes and the myelin they produce. While all leukodystrophies involve white matter pathology, this category is defined by conditions in which the myelin pathology is the central feature, rather than a secondary consequence of defects in other CNS cell types. These disorders are further subclassified based on characteristic MRI patterns and underlying pathomechanisms as either demyelinating, hypomyelinating, or associated with myelin vacuolization^5^.

### Demyelinating disorders

Demyelinating disorders are a subcategory of myelin disorders defined by the progressive loss of previously formed myelin. Here, myelin is initially deposited normally, but is later degraded, typically due to underlying metabolic defects^5^. On MRI, demyelination is marked by a distinctly low T1 signal intensity compared to gray matter structures, reflecting substantial loss of myelin^40^. Well-known examples discussed in this review include adrenoleukodystrophy, metachromatic leukodystrophy, multiple sulfatase deficiency, and cerebrotendinous xanthomatosis.

### Adrenoleukodystrophy

Adrenoleukodystrophy (X-ALD) is an X-linked disorder caused by mutations in *ABCD1*, impacting the protein responsible for transporting very long-chain fatty acids (VCLFA) into peroxisomes for β-oxidation^41^. Mutant ABCD1 results in an accumulation of saturated VLCFA, though the link between VLCFA buildup and disease severity and progression remains unestablished^42^. X-ALD manifests in males in different forms: a severe childhood cerebral form (ccALD) associated with inflammatory cerebral demyelination, a milder and slowly progressive adult-onset adrenomyeloneuropathy (AMN) characterized by axonopathy and peripheral neuropathy, an adult cerebral demyelinating form (acALD), and Addison’s disease, an adrenocortical insufficiency without neurological involvement^43–45^. Carrier women often develop signs of the disease, with a later onset AMN-like phenotype^46–48^. The cerebral form is associated with rapid disease progression, driven by inflammatory demyelination. It typically manifests initially as cognition or behavioral changes and progresses to both motor and cognitive decline, with most affected individuals reaching a vegetative state or succumbing to the disease within a few years after onset^43,44,49^. Estimates suggest that approximately 30–40% of children and 20% of adult males with an *ABCD1* mutation will develop cerebral demyelination^50^. However, no genotype-phenotype correlations have been established, and it remains unknown why some individuals develop the cerebral form^51–53^. Hematopoietic stem cell transplantation (HSCT) is a potential treatment for ccALD, and in some instances in acALD, when administered before neurological symptoms manifest^54,55^. In recent years, elivaldogene autotemcel (eli-cel), a lentiviral-mediated hematopoietic stem cell gene therapy (HSCT-GT), has been approved in the United States for early-stage ccALD, mitigating donor constraints and the risks of graft rejection, immune-mediated complications and the adverse effects of chemotherapy and immunosuppression associated with allogenic HSCT^56–59^. Therapeutic options for adults with AMN are more limited, as neither HSCT nor HSCT-GT are viable options for this condition. However, adeno-associated virus (AAV) *ABCD1* gene therapy has shown promising results in an AMN mouse model^60,61^, and a Phase 1/2 clinical trial (NCT05394064) is currently underway.

Generating iPSCs from X-ALD patients was a significant milestone in leukodystrophy research, representing one of the first leukodystrophy iPSC models. Indeed, iPSC studies have uncovered novel insights into X-ALD that traditional animal models and in vitro studies overlooked. These findings reveal specific cellular dysfunctions and highlighted the critical role of iPSCs in understanding the disease mechanisms. Investigations at the pluripotent stage have shown only mild X-ALD features, confirming that disease pathogenesis is restricted to specific cell types. Sequencing of ccALD iPSCs demonstrated upregulated pathways related to peroxisome abundance, oxidative stress, and neuroinflammation^62^. Most studies in X-ALD iPSCs have shown no increase in VLCFA levels^62,63^, with one exception^64^. In this case, the same iPSC line previously reported with low VCLFA levels^63^ was later reported as high in a subsequent publication^64^. This variability may reflect differences in passage number or individual culture techniques, highlighting the importance of ensuring reproducibility across laboratories and experimental conditionals,

The derivation and differentiation of iPSCs from both AMN and ccALD patients have enabled detailed research into cell-specific responses to disease processes. Differentiation to glial cells, including both oligodendrocytes and astrocytes, has exclusively focused on VLCFA accumulation, showing elevated levels in all X-ALD lines^63–67^. Unlike findings in patient fibroblasts or plasma^68^, VLCFA accumulation in glial cells correlated with disease severity^63–67^, supporting the importance of examining disease-relevant cells. Recent studies have confirmed this finding by examining VLCFA levels in white matter (WM) from postmortem AMN and ccALD individuals^69,70^. VLCFA levels in iPSC-derived neurons have shown conflicting results, precluding insights into this cell type^63,65^. Functionally, oligodendrocytes showed no differences in the presence of galactocerebroside and neurons had normal calcium signaling responses, however, more detailed studies are necessary to understand how X-ALD may affect these cells^65^. Interestingly, the enzyme CH25H, which catalyzes the conversion of cholesterol and 25-hydroxycholesterol (25-HC), showed expression levels correlating with disease severity in iPSCs and oligodendrocyte progenitor cells (OPCs)^64,71^. Introducing 25-HC to patient OPCs decreased VLCFA levels, while VLCFA addition increased CH25H expression^64,71^, suggesting a potential feedback mechanism between VLCFA accumulation and CH25H expression that is moderated by 25-HC in OPCs. Studies in mice have suggested that 25-HC is linked to inflammation and oligodendrocyte apoptosis through NLRP3 inflammasome activation^64^, however this has yet to be examined in iPSC patient models.

Recent studies focusing on astrocytic differentiation have provided strong evidence for the primary role of astrocytes in disease pathogenesis^65–67,72^. X-ALD-derived astrocytes recapitulate the hallmark VLCFA accumulation, and, upon VLCFA exposure, exhibit a heightened reactive state that is not observed in control astrocytes, suggesting an increased predisposition to reactive transformation^67,72^. Indeed, other studies have confirmed increased proinflammatory responses from X-ALD astrocytes, particularly those derived from ccALD patients, including heightened activation and cytokine production^65,66^. Mitochondrial abnormalities were also reported, showing disrupted cristae and fatty acid deposits, impaired electron transport and altered energetic profiles^66,67^. Molecular investigations have highlighted diverse pathways modulating differential inflammatory responses. ccALD astrocytes were associated with elevated Toll-like receptor expression and elevated STAT3 phosphorylation^66,67^, the former being previously reported in X-ALD mice^73^. Conversely, AMN astrocytes demonstrated a more protective response featuring heightened anti-inflammatory-associated factors oncostatin M and interleukin 6 (IL-6)^66^. While a previous study did not find significant IL-6 elevation in AMN astrocytes^65^, elevated levels have been reported in the plasma of AMN patients^74^. These findings are corroborated by investigations of activated astrocytes in X-ALD postmortem brains and recent studies indicating that astrocyte stress may precede myelin loss in X-ALD^75,76^. The role of astrocytes as possible drivers of disease has been further reinforced by co-culture studies in which X-ALD-derived astrocytes were cultured with control motor neurons. Here, neurons co-cultured with patient-derived astrocytes exhibited significantly reduced dendritic arborization, suggesting disease-associated astrocytes may impair neuronal development, potentially through reduced neuronal support^72^.

With the development of differentiation protocols capable of patterning less widely studied cell subtypes, such as brain microvascular endothelial cells (BMECs), detailed studies of the neurovascular unit (NVU) and blood-brain barrier (BBB) function have become possible. Disruption of the BBB is a notable hallmark of cALD^77^ and recent studies have shown that BBB dysfunction coincides with the onset of demyelination and is accompanied by changes in brain endothelium characteristics^78^. iPSC studies utilizing differentiated ccALD stem cells to BMECs have further confirmed altered BMEC properties in disease pathogenesis. These alterations are characterized by the accumulation of lipid droplets and leaky barrier function, which permits passive transport^79^. Transcriptomic findings revealed elevated expression of inflammation-related genes and reduced expression of genes related to intracellular attachment^79^. Interestingly, both iPSCs and human pathological studies have noted elevated *TGFB*, a regulator of endothelial barrier function, further endorsing iPSC-derived BMECs as a valuable model to study the BBB in X-ALD^78,79^.

### Metachromatic leukodystrophy

Metachromatic leukodystrophy (MLD) is a lysosomal storage disorder caused by biallelic pathogenic variants in the gene encoding arylsulfatase A (ARSA), causing excessive sulfatide accumulation^80^. Sulfatides are the major sphingolipids in myelin^81^ and their buildup leads to progressive demyelination, neuroinflammation, and neurodegeneration in both central and peripheral nervous systems^82–84^. MLD is associated with four clinical subtypes, classified according to the age of symptomatic onset: late-infantile, early-juvenile, late juvenile, and adult MLD. Earlier disease onset is associated with a more rapid progression and severity^85,86^. While HSCT has been a therapeutic option for several decades, current guidelines recommend its use only for pre- or early symptomatic individuals with late-juvenile and adult-onset forms^87–91^. Atidarsagene autotemcel (arsa-cel) is a recently approved autologous HSCT-GT available in Europe and the United States which has demonstrated benefits in attenuating disease progression and maintaining motor and cognitive function. However, its use is only approved for pre-symptomatic late-infantile and pre- and early-symptomatic early-juvenile subtypes^92–94^. Despite the known biochemical defect, specific mechanisms underlying MLD pathogenesis are under investigation.

iPSCs have primarily been derived from patients with the late-infantile onset form of MLD^95–97^, although a model derived from a juvenile-onset patient has also been reported^98^. However, in some studies, the clinical form was reported for certain iPSC lines but omitted for others, making it difficult to fully assess how well the different subtypes are represented across models. In their undifferentiated state, MLD iPSCs show minimal sulfatide accumulation but exhibit mild signs of lysosomal abnormalities, implying some lysosomal dysfunction occurs independently of sulfatide buildup. When differentiated into glial and neuronal cells, MLD lines progressively accumulate sulfatides^96^, recapitulating the biochemical impairments observed in brain autopsy tissue^99^. Neurons appear especially vulnerable in MLD, as evidenced by increased apoptosis in iPSC-derived MLD motor neurons^97^ and continual reduction of mature neurons in cortical cultures^96^ suggestive of cell death. Likewise, MRI studies confirm neuronal involvement, with gray matter (GM) volume decreasing prior to WM loss^100^. Additionally, mouse models display spinal neurodegeneration and hyperexcitability^101,102^. Despite these findings, iPSC-derived cortical neurons exhibit normal spontaneous activity when assessed with multielectrode arrays^96^, however, more detailed electrophysiology studies may be required. Regarding glial cells, iPSC-derived OPCs and astrocytes indicate potential maturation anomalies^96,97,103,104^, along with ramified astrocytic morphology indicative of astrogliosis^96^. However, additional studies on glial development using derivation protocols capable of mature terminal differentiation are required to definitively confirm these findings. Further molecular examination of iPSC-derived neurons and glia also implicates more intricate pathways and intracellular functioning in MLD. Specifically, MLD neuronal and glial cells mirrored lysosomal and mitochondrial abnormalities seen in iPSCs, marked by impaired mitophagy and lysosomal accumulation^96,97^. Moreover, increases in endoplasmic reticulum (ER) stress markers in these cells signify activation of the unfolded protein response (UPR)^97^, a pathway impacted in other lysosomal storage disorders, including Krabbe disease (described below)^105^. Advances in cell-mediated gene therapy using iPSCs have demonstrated potential for treating MLD by replacing damaged cells and addressing enzymatic deficiencies^95,98^. By overexpressing ARSA in deficient iPSCs, differentiated neuroepithelial, astroglia^98^ and neural stem cells^95^, showed corrected enzymatic activity and reduced sulfatide levels.

### Multiple sulfatase deficiency

Multiple sulfatase deficiency (MSD) is an ultra-rare lysosomal storage disease arising from biallelic pathogenic variants in *SUMF1*, which encodes formylglycine-generating enzyme (FGE)^106,107^. FGE is essential for post-translational activation of sulfatases, and its deficiency results in the functional impairment of all sulfatase enzymes leading to the accumulation of sulfatides and glycosaminoglycans (GAGs) and widespread cellular and tissue dysfunction^108–110^. MSD typically presents in infancy with a complex, pleiotropic phenotype that reflects the broad range of deficient sulfatases. It can be classified into two subtypes: a severe form, characterized by onset within the first month of life with severe multisystemic impairment and disability, and an attenuated form, marked by initial acquisition of developmental milestones followed by regression^111^. Non-neurological features such as ichthyosis, hepatosplenomegaly, and hearing loss are also common. Mouse models of MSD have revealed neuronal loss^112,113^ and astrocytic dysfunction^113^, though the precise disease mechanisms remain under investigation. While no therapies are currently available, preclinical studies exploring gene therapy^114^ and HSCT-GT^115^ have shown promising results.

Stem cell modeling of MSD has focused exclusively on neuronal differentiation, with studies showing no apparent defects in the ability of MSD iPSCs to differentiate into NPCs^116,117^ or neurons^117^. However, upon neuronal maturation, MSD neurons exhibited defects in neurite outgrowth and organization, as evidenced by decreased MAP2 and Tuj1 fluorescence and reduced DAPI clustering. While these findings can suggest impaired neuronal maturation and morphology, further studies are required to confirm these observations. Biochemically, sulfatase activity remained comparable to isogenic controls at the pluripotent stage, showing a trend toward reduced activity at the NPC stage, with a significant decline during neuronal differentiation. This reduction coincided with progressive GAG accumulation and increased lysosomal stress, including altered LAMP1 expression and reduced lysosomal size, indicating that mature neurons appear more vulnerable to *SUMF1* mutations^117^. Additionally, MSD-derived NPCs have been utilized to test the efficacy of retinoids identified through fibroblast-based drug screening. Interestingly, sulfatase activity in this MSD NPC line was significantly reduced compared to a healthy control, providing an opportunity to evaluate the effects of tazarotene and bexarotene treatment, which successfully increased sulfatase activity^116^.

### Krabbe disease

Krabbe disease (KD) is an autosomal recessive disorder caused by a deficiency of galactosylceramidase, an enzyme tasked with degrading sphingolipids found in myelin. Biallelic pathogenic variants in *GALC* cause a buildup of galactosylsphingosine (psychosine), disrupting lysosomal metabolism primarily in oligodendrocytes and Schwann cells^118^. KD is also known as globoid cell leukodystrophy due to its unique neuropathology characteristic infiltration of multinucleated macrophages (globoid cells) in the cerebral WM^119,120^. Disease onset for the classical form of KD (early infantile form) occurs before 12 months of age and is associated with rapid neurodegeneration and early death, typically before 2 years of age^121,122^. Later onset forms include late infantile (1–3 years), juvenile (4–17 years), and adult (>18 years), which are associated with more variable clinical outcomes^122^. HSCT can decelerate disease progression in presymptomatic individuals, with treatment yielding a more favorable prognosis in later-onset forms compared to the early-infantile form, where motor outcomes remain poor despite intervention^123–125^. A clinical trial is underway for intravenous gene therapy in previously transplanted patients with early and late infantile Krabbe disease (NCT05739643)^126^.

Though a plethora of KD animal models have spontaneously arisen over the years, allowing for detailed study in multiple vertebral systems^127–131^, iPSCs have been instrumental in delineating human-specific pathology and complex cellular interactions contributing to disease. As with most lysosomal storage leukodystrophies, key biochemical features of reduced GALC enzymatic activity and psychosine accumulation were mildly recapitulated in iPSCs^132,133^, and further increased during differentiation towards neuroglial lineages^132–134^. Transcriptomic profiling of NPCs derived from a patient with KD revealed upregulation of genes involved in the MAPK, PI3K-Akt, and cAMP signaling pathways^133^. These pathways regulate key cellular processes including proliferation, survival, and neuronal function, and their dysregulation has been implicated in KD disease^135–137^. Oligodendrocytes were traditionally thought to be the most affected cells in KD as galacosylceramidase is highly expressed in the myelin sheath. Differentiation of patient iPSCs to mixed glial cultures showed decreased expression of lineage markers OLIG2 and APC, suggesting impaired maturation^132^. However, this decrease could be attributed to proliferative or apoptotic insults, and notably, cultures were only maintained for 24 days, a short time period for oligodendrocyte maturation. Studies of iPSC-derived neurons highlight patient-specific disease manifestations, likely reflecting either disease heterogeneity, small sample size or the limitations inherent to a generic mixed CNS cell differentiation protocol. Specifically, one patient line exhibited normal neuronal development while the other displayed reduced neuronal counts, maturation deficits, proliferative decline, lysosomal anomalies, premature senescence, and an abnormal lipid profile. No functional analyses were reported, and it remains unclear whether these defects are neuron-autonomous or result from culture heterogeneity^132^. Recent evidence in iPSCs has also implicated a distinct astrocytic phenotype from GALC deficiency^134^, corroborated by studies in psychosine-treated human cells^138,139^. iPSC-derived astrocytes exhibit psychosine buildup and heightened IL-6 secretion, suggesting an intrinsic inflammatory role^134^. Moreover, disruptions in lipid biosynthesis pathways were noted, with elevated glucosylceramide levels indicative of an adaptive lysosomal metabolic response^134^. This finding has been reported in the CSF of a KD canine model^140^. In co-culture, KD astrocytes impaired the survival of iPSC-derived neurons from healthy controls, partly explaining the neuronal abnormalities reported in mixed glial cultures, while paradoxically promoting microglial survival^132,134^. Differentiation of KD-derived iPSCs to microglia recapitulated the characteristic globoid cell formation, which was further exacerbated upon exposure to exogenous galactosylceramide^141^. Examination of lysosomal function revealed distinct responses between control and KD microglia under galactosylceramide overload, with KD microglia showing early accumulation of autophagic proteins and reduced LAMP1 expression. Over time, KD globoid cells showed signs of recovery, suggesting a compensatory mechanism involving lysosomal biogenesis^141^. Using the same patient-derived iPSCs, a 3D myelinating organoid (mOrg) model was developed to study neurodevelopment and myelination in a more complex system^141^. These mOrgs showed no significant differences in overall development, including the generation of neurons, astrocytes, and oligodendrocytes, contrasting with previous findings from 2D cultures that reported reduced neuronal and oligodendrocyte counts^132^. Upon maturation in pro-myelinating media, KD-derived mOrgs first exhibited signs of demyelination after 12 weeks, characterized by a reduction in MBP+ internodes. Notably, myelin loss did not correlate with changes in lysosomal or autophagic proteins and occurred in the absence of microglia, suggesting that initial myelin loss is independent of lysosomal impairment and independent of the globoid cell phenotype^141^.

Gene therapy approaches in iPSCs and their derivatives have demonstrated the usefulness of targeted genetic correction for improving disease-specific phenotypes. Specifically, lentiviral-mediated GALC correction in iPSCs and their derivatives restored GALC enzymatic activity and reduced psychosine levels^132^. Similarly, adeno-associated virus (AAV) serotype testing has been conducted in both patient-derived NPCs^142^ and cOrgs^143^. While both studies tested multiple AAV serotypes, the optimal serotype differed between models, with AAV2 performing best in NPCs and AAV5 in cOrgs, likely due to differences in cellular composition and gene expression. Transduction with the respective AAV-GALC vectors restored GALC activity and reduced psychosine accumulation^142,143^. Both studies also evaluated AAVrh10, which showed some potential but was not among the top-performing candidates. However, clinical trials for KD are currently underway using the AAVrh10 vector in combination with HSCT (NCT05739643)^126^.

### Cerebrotendinous xanthomatosis

Cerebrotendinous xanthomatosis (CTX) is a congenital metabolic disorder stemming from an insufficiency of the mitochondrial enzyme sterol-27-hydroxylase, due to biallelic variants in *CYP27A1*^144–146^. This enzyme catalyzes hydroxylation reactions in bile acid synthesis and cholesterol processing. Dysfunction results in decreased bile acid synthesis and the toxic accumulation of cholestanol and cholesterol (70), leading to diverse and multisystemic clinical manifestations, ranging from chronic diarrhea, juvenile cataracts, tendon xanthomas, premature arteriosclerosis, osteoporosis and progressive neurological involvement including ataxia, dystonia, epilepsy, dementia and psychiatric manifestations^147–150^. CTX is one of the few treatable leukodystrophies as supplementation with chenodeoxycholic acid (CDCA) or cholic acid, with or without an inhibitor of 3-hydroxy-3-methylglutaryl coenzyme A (HMG-CoA) reductase, can improve symptoms and stabilize or prevent further progression depending on time of intervention^151–153^. The mechanisms of how cholestanol and cholesterol accumulation in the cerebrum and cerebellum drive disease are largely unknown.

Only one study utilized iPSCs derived from a CTX patient with disease onset at 2 years old^154^, focusing on neuronal differentiation due to the prominent axonal degeneration seen on pathology^155^. Long-term culture of CTX-derived cortical glutamatergic neurons led to a reduction in axonal length and axonal swellings, mirroring typical neurodegeneration. As anticipated, cholesterol accumulated within CTX neurons, alongside reduced levels of the downstream product 27-hydroxycholesterol. No examination of cell death, proliferation, or neuronal activity was reported. Administration of CDCA mitigated cholesterol buildup and ameliorated axonal swelling^154^, offering direct evidence of CDCA’s impact on neurons and suggesting the potential for reversal of certain abnormalities. However, it is clear that not all phenotypes are reversible with CDCA intervention^151^, suggesting some aspects of neuronal function are not recoverable, though this is likely dependent on the timing of intervention in disease course.

### Hypomyelinating disorders

Hypomyelinating disorders are another subcategory of myelin disorders characterized by a permanent lack of myelin, rather than progressive loss of previously formed myelin^5,156^. MRI findings typically show cerebral white matter that is hyperintense, isointense, or only slightly hypointense relative to the cortex on T1-weighted images, with diffuse hyperintensity on T2-weighted images^40,157^. Examples discussed in this review include Pelizaeus-Merzbacher disease, POLR3-related leukodystrophy, and oculodentodigital dysplasia.

### Pelizaeus-Merzbacher disease

Pelizaeus-Merzbacher Disease (PMD) is the prototypical hypomyelinating leukodystrophy. It is an X-linked recessive disease caused by pathogenic variants in *PLP1*, encoding proteolipid protein 1 (PLP1), constituting one of the major CNS myelin proteins and its splice isoform DM20^158,159^. Patients with the most common classic PMD presentation, mainly caused by *PLP1* duplications, typically demonstrate onset of nystagmus during infancy, delayed motor development with axial hypotonia, progressive spasticity, and cerebellar signs^160–162^. The most severe form of the disease, known as connatal PMD, is associated with rapid disease progression after a neonatal onset, leading to early death in childhood^162^. Other PLP1-related disorders associated with different genetic mutations in *PLP1* demonstrate a range of phenotypes, including Hypomyelination of Early Myelinating Structures (HEMS)^163–166^, and milder presentations in PLP1-null syndrome^167,168^ and Spastic Paraplegia Type 2 (SPG2)^169,170^. The phenotypic severity of PMD and PLP1-related disorders is related to PLP1 dosage and functionality, including the degree of negative impact genetic variants have on cell dysfunction and cell death^161^. Further, the most severe form, connatal PMD, is caused by sequence variants resulting in protein misfolding, leading to downstream toxic pathophysiological mechanisms ultimately causing oligodendrocyte apoptosis^171–176^. While less severe, classic PMD is typically associated with protein overexpression caused by genetic duplications, which results in protein accumulation and mislocalization leading to oligodendrocyte dysfunction and death, and thus impairments in myelination^177–179^. In contrast, the milder disease forms are associated with loss of functional PLP1 protein, including PLP1-null syndrome (associated with null *PLP1* variants) and SPG2 (typically associated with missense variants). These forms do not directly impact cell survival, and therefore more subtle abnormalities are present in myelin formation^180–184^. These diverse genotypic and clinical presentations can present a challenge to therapy design and development. However, clinical trials are currently underway for iron chelator deferiprone (EudraCT Number: 2021-000070-29) and ION355 (NCT06150716), an ASO targeting the *PLP1* duplication for patients with classic PMD^185^.

As PLP1 is expressed predominantly in oligodendroglial and pluripotent stem cells, iPSC studies have shed insight into how specific mutations alter PLP1 expression^186^ and affect pathology^187–190^. Most prominently, iPSCs permit examination of OPCs and oligodendrocytes derived from patients with diverse clinical and genetic presentations, revealing considerable individual variation in cellular disease mechanisms. These studies emphasize the importance of incorporating multiple cell lines to fully capture the heterogeneity and complexity of disease^188^. Commonly, all iPSC studies demonstrated defects in the oligodendroglial lineage, though the nature of these defects varied among individuals^187,188,190^. Reduced numbers of OPCs were observed in many patient lines, though it remains unclear where this defect originates^188,189^. Upon their maturation to oligodendrocytes, PMD patient lines can be categorized into one of three categories based on cellular properties: an absence of mature oligodendrocytes, lack of PLP1 expression, or perinuclear retention of PLP1^188^. These classifications correlate with the genetic and clinical profiles of individuals as patients with *PLP1* deletions exhibit abundant but immature oligodendrocytes without PLP1 expression, those with duplications show perinuclear retention of PLP1 and patients with point mutations may fall into any of the three categories^188,189^. Oligodendrocytes able to myelinate show a reduced frequency and thickness of the myelin sheath in both 2D^187^ and 3D cultures^189^. Further scrutiny of derived OPCs and oligodendrocytes implicates ER stress as a potential pathogenic mechanism, often also exhibiting perinuclear retention of PLP1 protein^187–189^. Addressing ER stress with small molecules improved oligodendrocyte maturation and myelination in some lines^188,189^. However, as ER stress is not universally observed, it cannot fully account for oligodendrocyte defects. A recent iPSC study proposes a more consistent pathogenic mechanism across genotypically diverse PMD patient lines of increased oligodendrocyte cell death^190^. Specifically, PMD iPSCs showed no defects differentiating into pre-myelinating oligodendrocytes, but upon terminal differentiation underwent apoptosis as well as ferroptosis, a form of programmed cell death characterized by iron-dependent lipid peroxidation. These PMD OPCs displayed an increased sensitivity to iron, characterized by dysregulated genes for iron metabolism and transporter expression, and increased lipid peroxidation, findings that were confirmed in primary human OPCs and the Jimpy mouse model. Iron chelation with deferiprone led to a full phenotypic rescue, providing the basis for the current clinical trial (2021-000070-29)^190^. This evidence suggests that the reduced oligodendrocyte numbers reported in previous studies may be attributable, in part, to apoptotic and ferroptotic cell death, perhaps combined with stalled maturation in some cells as suggested by reduced morphological complexity. Overall, iPSC studies have suggested multiple interplaying mechanisms contributing to PMD pathology varying with genotype.

### POLR3-related leukodystrophy

RNA polymerase III-related hypomyelinating leukodystrophy (POLR3-HLD) is caused by biallelic pathogenic variants in genes encoding specific subunits of the transcription enzyme RNA polymerase III (Pol III). Due to its key clinical features involving a combination of hypomyelination, hypodontia, and hypogonadotropic hypogonadism, it is also known as 4H leukodystrophy^191^. Disease onset usually begins with motor delay or regression in childhood, evolving to further neurological involvement including cerebellar features, pyramidal and extrapyramidal signs^191–193^. The phenotypic spectrum of disease associated with variants in Pol III subunit genes also involves severe presentations, such as the early onset severe striatal form^194–196^, and milder presentations, such as the mild striatal form^196–198^, the spastic ataxia/paraparesis form^199–204^, patients with isolated hypogonadotropic hypogonadism^205^ or without hypomyelination^206^, and those incidentally diagnosed with brain MRI^194,207^. Causal genetic variants have been discovered in genes encoding five different subunits of Pol III: *POLR3A, POLR3B, POLR1C, POLR3D*, and *POLR3K*^208–213^. In total, Pol III is composed of 17 subunits, and functions to transcribe several non-coding RNAs with essential cellular roles (e.g., gene expression, RNA processing, and protein synthesis), including tRNAs, 5S rRNA, 7SL and 7SK RNA, and U6 snRNA, along with other vault, small nuclear, and micro-RNAs^214–217^. The causal relationship between hypofunction of this ubiquitously expressed transcription complex and brain hypomyelination is thought to result from either a lack of production of Pol III-specific transcripts important for oligodendrocyte and/or neuron function, or a general decrease in transcriptional capacity and global protein synthesis during critical periods of brain development^218,219^. These hypotheses are not mutually exclusive, and both mechanisms are likely involved.

To further study neurological phenotypes and pathophysiological mechanisms of POLR3-HLD, in one study, patient-derived iPSCs were used to research abnormalities in cerebellar and cortical cells, and to investigate neuron and oligodendrocyte co-culture interactions^220^. In iPSC-derived cerebellar granule cells and cortical neurons (including a mix GABAergic interneurons and glutamatergic cortical projection neurons), RNA sequencing showed downregulation of *ARX*, a gene associated with cortical interneuron development and migration. Additionally, this result was confirmed using the brain tissue of two POLR3-HLD patients, which supported alterations in cortical interneuron involvement. Decreased GABAergic synapses in iPSC-derived cortical interneurons, along with increased network activity and decreased GABAergic signaling were also seen. Additionally, when investigating subtypes of cortical interneurons, the parvalbumin interneuron lineage (via *ERBB4* expression) was increased in patient cultures, suggesting specific involvement of this cell type. Finally, co-cultures of control and patient oligodendrocytes and cortical neurons did not yield significant abnormalities, however, there was variability between patient lines regarding the number of oligodendrocyte lineage cells generated. As co-cultures involved a mix of GABAergic and glutamatergic neurons, additional studies using isolated neuron populations may provide further insight into myelination defects^220^.

Additionally, iPSCs have been studied from patients with the spastic ataxia/paraparesis form^199^. Patients did not demonstrate hypomyelination and had a milder disease course compared to typical POLR3-HLD, and the majority harbored a splice variant (1909 + 22 G > A) on one allele causing activation of a cryptic splice site and production of an aberrant splicing product. iPSCs from patients were generated and differentiated into neuroepithelial cells to study levels of the aberrant splicing transcript in different cell types, with neuroepithelial cells showing greater expression and therefore higher activation of the cryptic splice site compared to iPSCs^199^.

### Oculodentodigital dysplasia

Oculodentodigital dysplasia (ODDD), named for its cardinal clinical features of ocular, dental, and digit anomalies, is caused by autosomal dominant, and more rarely, recessive, mutations in *GJA1*^221,222^. Patients frequently exhibit distinctive facial characteristics, and a subset may develop cardiac and neurological manifestations^222^. Notably, only 30% of patients present with hypomyelination^223,224^. *GJA1* encodes Connexin 43 (Cx43), a gap junction (GJ) hemichannel protein enabling adjacent cell communication and transfer of small molecules^225^. ODDD-associated mutations have been shown to impair hemichannel and GJ functionality through various mechanisms^226,227^, yet it is unclear why some patients develop hypomyelination and not others. Few disease models have been developed to study the leukodystrophy phenotype of ODDD^226,228,229^, resulting in a limited understanding of how the CNS is affected. As Cx43 is predominantly expressed in astrocytes, where it facilitates astrocyte-astrocyte and astrocyte-oligodendrocyte communication^230^, pathogenic mechanisms are thought to arise due to altered astrocyte functionality, formally classifying this disorder as an astrocytopathy. However, we have opted to classify it under myelin disorders due to its distinctive hypomyelination observed on brain MRI and the current lack of functional evidence directly implicating astrocytes in its pathogenesis. Nevertheless, it is acknowledged that both classifications may be valid. Interestingly, loss-of-function of connexin 47 causes the hypomyelinating disorder Pelizaeus-Merzbacher-like disease (PMLD)^231^, suggesting a shared mechanism stemming from disrupted astrocyte-oligodendrocyte communication via Cx43-Cx47 GJs. Currently, there are no approved treatments, active clinical trials or preclinical therapies for ODDD.

Only two studies to date have utilized patient-derived iPSCs from individuals with ODDD, both of whom presented with prominent neurological manifestations^232,233^. Although brain MRI findings were not reported in these cases, we included these models because neurological manifestations in ODDD are almost invariably associated with leukodystrophy^221,223,224,234^. Investigations into these iPSCs revealed both decreased Cx43 expression and GJ dysfunction^232,235^. Differentiation to osteoblasts and chondrocytes to model craniofacial development revealed reduced Cx43 expression and impaired maturation and calcification in ODDD-derived osteoblasts^232^. Notably, evidence from mouse models suggests that osteoblast maturation is delayed rather than arrested^236,237^. This suggests that the impaired maturation observed in this study may also represent a delay rather than a complete developmental halt, as cultures were only maintained for 20 days^232^. Chondrocyte differentiation was normal; however, cartilage formation was structurally irregular, with Cx43 showing homogenous distribution rather than localization at GJ plaques, indicating altered intercellular communication or signaling^232^. Differentiation into adipocytes did not reveal abnormalities, unless the intermediate stage mesenchymal stem cells (MSCs) were differentiated at a higher passage^233^. Given that passaging cells do not represent in vivo disease conditions and no abnormalities in adipose tissue have been reported in ODDD patients, this finding may not directly contribute to our understanding of disease pathogenesis.

Given the prominent ophthalmic features linked to ODDD, including microphthalmia, microcornea, iris abnormalities, glaucoma, cataracts, and reduced vision^222^, generation of retinal organoids from a *GJA1* CRISPR-mediated KO iPSC line helped facilitate a specific investigation of ocular manifestations. *GJA1* KO organoids were significantly smaller with a thinner neural retina, attributed to loss of polarity in retinal progenitor cells (RPCs), rather than alterations in proliferation or cell death. Moreover, apical-basal polarity plays a crucial role in determining RPC cell fate, and disrupted polarization leads to a surplus of RPCs while diminishing the abundance of mature retinal cell types. The mechanisms by which Cx43 affects cell polarity remains unclear but one potential cause may involve disruption in interkinetic nuclear migration or cytoskeletal trafficking. Despite these changes, the overall neuroarchitecture remained intact^235^.

While these iPSC models have proven useful for examination of systemic manifestations of ODDD^232,233,235^, no studies to date have utilized iPSCs to model the CNS defects underlying hypomyelination. Developing CNS-specific iPSC models derived from patients with ODDD-related leukodystrophy would provide the first insights into the mechanisms driving hypomyelination and pave the road for the development of therapeutics for this disease.

### Myelin vacuolization

Disorders of myelin vacuolization refers to a subcategory of myelin disorders characterized by spongy degeneration of the white matter due to intramyelinic edema or splitting of the myelin layers. Unlike demyelination or hypomyelination, where myelin is lost or never properly deposited, vacuolization reflects structural disruption of existing myelin^4,5^. While the pattern of myelin vacuolization differs, typically on MRI, the white matter is hypointense on T1 and significantly hyperintense on T2, and may involve restriction diffusion in early stages, especially for leukodystrophies associated with myelin microvacuolization, such as some mitochondrial leukodystrophies and CLNC2-related leukodystrophy, among others^40,238,239^. An example of a vacuolating leukodystrophy discussed within our review is Canavan disease.

### Canavan disease

Canavan disease (CD) is caused by biallelic pathogenic variants in *ASPA* which encodes the oligodendrocyte-specific enzyme aspartoacylase, responsible for hydrolyzing N-acetylaspartic acid (NAA) into acetate and aspartate^240,241^. NAA is an intermediate metabolite predominantly produced by neurons and a crucial source of acetate for myelin lipid synthesis by oligodendrocytes^242,243^. ASPA deficiency leads to elevated NAA levels^244,245^ resulting in progressive myelin spongiform vacuolation^246^. The classical form of CD typically manifests in infancy with macrocephaly, ataxia, and hypotonia. Congenital and juvenile forms are less common and represent the severe and milder ends of the disease spectrum, respectively^247^. Studies in rodent models have shown a variety of possible disease mechanisms, including decreased acetate impairing oligodendrocyte myelination, disrupted osmoregulation causing cellular swelling, oxidative stress, and excitotoxicity arising from high NAA concentrations^248–253^. These mechanisms may not be mutually exclusive and further exploration in a broader range of disease models would help elucidate their relative contributions to disease pathogenesis. *ASPA* gene replacement therapy has shown promise in preclinical mouse models^254,255^, and two Phase I-II clinical trials are underway using intravenous delivery of rAAV9 (BBP-812; NCT04998396) and intracerebroventricular delivery of rAAV-Olig001-ASPA (NCT04833907)^256^.

iPSC research for CD has primarily explored therapeutic avenues via cell transplantation in CD mouse models^257,258^. Using lentiviral vectors or CRISPR-Cas9 to restore ASPA activity in patient iPSCs, these studies demonstrated that transplantation of derived NPCs^257,258^ and OPCs^257^ can improve survival and motor function, as well as reduce NAA levels and spongiform degeneration in the transplanted mice. Recently, mOrg have been derived from CD iPSCs utilizing a novel differentiation protocol^259^. Interestingly, mOrg did not show expected myelination defects, which was attributed to frequent media changes that prevented NAA accumulation, suggesting NAA buildup is necessary to cause myelin loss. Moreover, experimental exposure of patient-derived mOrgs to NAA recapitulated typical CD myelin sheath destruction and vacuolation, with reductions in the number and length of myelin sheaths. Despite these abnormalities, overall organoid structure remained intact, with neurons, astrocytes, and OPCs maintaining normal morphology and distribution, indicating particular susceptibility of myelin. Interestingly, *ASPA*-genetically corrected CD iPSCs exhibited no adverse phenotypes upon NAA treatment, demonstrating the ASPA’s ability to counteract the toxic effects of elevated NAA. Acetate levels remained unchanged after NAA treatment, lending further support to the hypothesis that NAA toxicity, rather than acetate deficiency, plays a crucial role in the pathology of CD.

### Astrocytopathies

Astrocytopathies are a group of leukodystrophies caused by defects in astrocyte function or by mutations in astrocyte-related genes^5^. Examples discussed in this review include Alexander disease, Vanishing White Matter, and Aicardi-Goutières syndrome.

### Alexander disease

Alexander Disease (AxD) arises from dominant mutations in the gene encoding glial fibrillary acidic protein (GFAP), an astrocytic intermediate filament^260^. AxD can be classified as type I or II, with the former representing the classical early-onset presentation of developmental delay, motor regression, macrocephaly, and seizures while the latter tends to present later in life with autonomic dysfunction and bulbar symptoms^261^. AxD-associated mutations lead to elevated GFAP levels, aggregation of Rosenthal fibers (RF), and astrocytic dysfunction^262,263^. Additional pathogenic mechanisms include impaired proteasome function, inflammation, altered metabolism, and disrupted neuronal support functions^263^. However, much of our understanding is derived from AxD murine models, which may not fully reflect the human disease, necessitating the need to determine which mechanisms are most relevant to AxD pathogenesis. While no commercial therapy exists for AxD, a phase 1–3 clinical trial (NCT04849741) using a GFAP-targeting ASO called zilganersen (ION373) has been underway since 2021^16,264^.

iPSC studies have largely focused on astrocytic differentiation and downstream studies^265–270^. The characteristic AxD pathology of increased GFAP expression and aggregation with RF formation has been recapitulated in iPSC-derived astrocytes^265–269,271–273^. Notably, these astrocytes showed changes in organelle distribution and enlarged vesicles concentrated in the cell body rather than cell processes^266^. Morphologically, studies have reported conflicting findings, with some observing no significant changes in astrocyte shape or complexity^265^, while others have described AxD-derived astrocytes as having larger cell bodies and shorter processes^273^, a phenotype also reported in in vitro studies using an AxD mouse model^274^. Functional studies also revealed elevated inflammatory cytokine levels^265^ and altered ion homeostasis^266^, consistent with findings in embryonic stem cell (ESC) knock-in astrocytes^271^ and animal models^275,276^. Specifically, AxD-derived astrocytes exhibited slower and less extensive calcium propagation and increased intracellular calcium levels, attributed to impaired extracellular ATP release^266,271^. Transcriptomic analysis revealed dysregulated pathways related to protein transport^266^, cellular adherence^265^, membrane composition and regulation^266,267^, immune response^267^, and cytokine production^267^, further corroborating these morphological and functional deficits. One study utilizing the iPSCs derived from an individual with the infantile-onset variant p.R239H, proposed that Ser13 phosphorylation of GFAP may serve as a marker of the infantile (type I) form^268^. Elevated levels were found in the post-mortem cortex tissue of AxD patients who died in infancy or childhood and iPSC-derived astrocytes exhibited perinuclear inclusions of pSer13-GFAP, compared to diffuse expression in the isogenic line^268^. While this finding is contradictory to the observation of selective Ser13 phosphorylation, the authors indicated discrepancies could be due to the in vitro culture conditions^268^. Caspase-6 expression and GFAP cleavage correlated with pSer13-GFAP in AxD astrocytes but not the isogenic control cells, suggesting pSer13-GFAP may trigger caspase-6 to cleave GFAP, contributing to protein aggregation^268,277^. These studies need to be further validated in additional patient iPSC lines.

Recent iPSC studies have implicated more intricate pathogenic mechanisms in AxD pathogenesis, including altered regulation of cell death, disrupted mitochondrial transfer, abnormal mechanotransduction, and altered cellular interactions in co-culture models. Among these, anastasis has been proposed as a potential mechanism in AxD pathology. One study found that AxD astrocytes exhibited increased indicators of cellular senescence, yet some astrocytes resisted apoptosis by entering a recovery phase known as anastasis. The senescent astrocytes secreted cytokines toxic to neurons, which may contribute to the neurodegeneration seen in disease progression^278^. Another study proposed that AxD-associated mutations led to decreased intercellular mitochondrial transfer between astrocytes, although the impact of this on disease pathogenesis is unknown^272^. Additionally, other studies have implicated an altered mechanical environment in AxD astrocytes. Specifically, patient-derived astrocytes showed increased expression of stress fibers and associated components^279^ or upregulation of extracellular matrix related genes^273^. These changes are thought to alter the rigidity of these astrocytes, as brain tissue from AxD mouse models exhibited increased stiffness compared to wild-type mice^279^. This altered mechanical environment may contribute to impaired OPC proliferation, migration, and oligodendrocyte myelination potential, as increased substrate stiffness has been shown to negatively impact these processes^263,280^. In co-culture studies, OPCs derived from healthy control iPSCs exhibited reduced proliferation and impaired maturation into myelinating oligodendrocytes when cultured alongside astrocytes derived from AxD iPSCs. However, these oligodendrocytes retained the ability to myelinate nanofibers effectively, indicating that white matter defects in AxD may be primarily linked to impaired oligodendrocyte maturation^267^. This effect is thought to be mediated by chitinase-3-like protein 1 (CHI3L1), a secreted neuroinflammatory-related protein found to be overexpressed in AxD astrocytes and patient brain tissue, which, when knock-downed, rescued OPC defects^267^. A recent study investigated the impact of AxD astrocytes on neurodevelopment using organoid models and co-cultures with either AxD or isogenic neurons^270^. In co-cultures with AxD astrocytes, there was an increased proportion of immature astrocytes and neurons, while organoids exhibited a reduction in both astrocytes and neurons compared to controls, suggesting impaired neuro-glia differentiation. Single-cell transcriptomics further revealed a shift toward non-neuronal differentiation, with increased mesoderm- and endoderm-derived populations in AxD co-cultures and organoids. Gene ontology analysis supported these findings, showing downregulation of pathways related to gliogenesis, forebrain development, and cell fate commitment^270^. Whether the increased mesenchymal-like population is relevant to AxD pathology remains unclear, as it may be an artifact of iPSC culture rather than a pathophysiological event.

### Vanishing white matter

Vanishing white matter (VWM), also known as childhood ataxia with central hypomyelination (CACH), is a prevalent leukodystrophy that can manifest at any age but typically emerges in early childhood^281,282^. It is marked by progressive deterioration in neurological function with predominant cerebellar ataxia, exacerbated by episodes of rapid decline triggered by external stressors such as fever and minor head injuries^283^. Biallelic variants in one of five genes encoding subunits of eukaryotic translation initiation factor eIF2B, including *EIF2B1*, *EIF2B2*, *EIF2B3*, *EIF2B4*, and *EIF2B5*, cause VWM^284,285^. eIF2B functions as a guanine nucleotide exchange factor for eIF2, a GTP-binding factor responsible for the delivery of initiator methionyl-tRNA to the ribosome. eIF2B also acts as a primary regulator of the integrated stress response (ISR)^286^. Distinctive astrocyte involvement is central to VWM pathology, characterized as immature and dysmorphic, coupled with an increased density of OPCs despite also showing a paucity of myelin^287^. While no commercial therapies have been approved, clinical trials for ISR modulators, fosigotifator (NCT05757141; NCT06594016) and guanabenz (EudraCT Number: 2017-001438-25), are currently in progress for VWM and preclinical studies using gene therapy in VWM mouse models have shown promising results^288^.

iPSC models of VWM have reinforced the critical involvement of astrocytes in disease pathology. Differentiation and phenotyping of VWM iPSCs to neuronal and oligodendroglial-like cells did not reveal notable abnormalities^289^. However, cOrg models exhibited impaired early neuronal development and reduced proliferation leading to smaller organoid size^290^. These observations may represent more severe forms of VWM, attributed to inherent metabolic and oxidative stress associated with differentiation exacerbating disease phenotypes. Impaired astrocyte differentiation has been reported in both 2D^291,292^ and 3D models^293^, except for one study possibly attributed to differences in differentiation protocols^289^. Consistent astrocytic findings across iPSCs, mouse models, and post-mortem tissues show elevated expression of the GFAP isoform, δ-GFAP^289,293^, and increased proliferation^291,292^. Only one study has derived both white and gray matter astrocyte subtypes^292^, citing greater transcriptomic dysregulation in WM astrocytes compared to GM. Specifically, WM astrocytes showed dysregulation in several pathways (i.e., immune system, extracellular space, cellular development, neuronal functioning, and vasculature), thus supporting the vulnerability of WM astrocytes to VWM-causing mutations. Explorations into the contribution of astrocytic dysfunction to CNS myelin rarefaction have revealed impaired oligodendrocyte maturation when exposed to VWM iPSC astrocyte-conditioned media^290,292^, alongside reduced myelination in organoid models^293^. No increase in cell death was observed in co-cultures of WM astrocytes and oligodendrocytes, indicating that the observed defects result from impaired oligodendrocyte maturation, rather than toxicity^292^. Proteomic analysis of conditioned media highlighted a downregulation in extracellular matrix (ECM) components and an upregulation of cytoskeletal and organelle components, combined with a reduction in the secretion of key factors essential for oligodendrocyte lineage differentiation and survival^290^. Specifically, hyaluronic acid (HA), an ECM substrate known to accumulate in VWM white matter and inhibit OPC differentiation^294^, was highly secreted by VWM astrocytes compared to controls^290^. Treating conditioned media with hyaluronidase to target HA restored oligodendrocyte maturation levels^292^, further emphasizing HA’s role in disease.

Various pathophysiological mechanisms have been proposed, however, inconsistencies between various disease models have hampered the identification of a unifying disease hypothesis. Previous in vivo and in vitro studies have demonstrated that VWM pathogenesis stems from dysfunction of the integrated stress response (ISR) rather than changes in the rate of protein synthesis^295–297^.However, discrepancies did exist between mouse models in the specific mechanisms underlying ISR dysregulation^298,299^. iPSC-derived astrocytes have confirmed findings of normal protein synthesis^291^ although the ISR responses varied dependent on the immunostimulant utilized^299^. Further, *ATF4* expression, a key ISR-related mRNA associated with VWM^299^, was not highly expressed in most stress-induced conditions. While the ATF4 transcriptome was not examined in this study, its activation has been reported in VWM mouse and patient brain tissue^299^. Intriguingly, VWM-derived astrocytes also showed a dampened unfolded protein response (UPR) with gene ontology categories from proteomic analysis indicating abnormalities in these pathways^291^. Results should be interpreted with caution given that the iPSC-derived astrocytes in this study did not faithfully recapitulate common findings in VWM. Signs of protein stress have also been associated in other iPSC-derived VWM astrocyte cultures, including elevated expression of αB-crystallin^289,293^, a protein chaperone^300^, provided further evidence of deregulation in these pathways. Another possible disease mechanism linked to VWM involves compromised mitochondrial function and impaired oxidative phosphorylation (OXPHOS)^287^. While reduced metabolic activity is a consistent observation amongst disease models and post-mortem tissue, the specific underlying mechanisms differ among them^301–304^. Two patient-derived astrocytes lines have demonstrated a minor reduction in mitochondrial membrane potential and increased presence of ROS, suggestive of an impairment in the ETC function^291,305^. Total ATP production was also unaffected^291^, contrasting findings obtained with murine primary glial cultures^301,303^. Proteomic and transcriptomic analysis corroborated these findings, revealing distinct metabolic signaling disruptions in glycolysis and tricarboxylic acid pathways^291^ and mitochondrial functioning^291,292^. Low levels of neuroinflammation have also been associated with VWM and an asthenic inflammatory response has previously been hypothesized to play a role in disease progression^287^, with iPSC studies showing little to no evidence of an overly reactive astrocyte responses^291,292^.

Few iPSC studies have explored potential therapeutic approaches. Preclinical studies for ongoing clinical trials have primarily focused on rodent models^288,299,306–308^. One iPSC study conducted a large-scale drug screen of 2400 FDA-approved drugs in patient-derived astrocytes, using ISR dysregulation (via CHOP and GADD34 expression), oxidative stress, and mitochondrial membrane potential as readouts. Edaravone (antioxidant), urosodiol (bile acid derivative), and zileuton (anti-inflammatory) emerged as promising candidates, suggesting that anti-inflammatory compounds may be beneficial for VWM treatment^305^. Interestingly, ISR modulators ISIRB and guanabenz, which have shown promise in mouse models and of which guanabenz is currently in clinical trial, did not demonstrate significant benefit in patient-derived astrocytes. Other compounds currently in clinical trials were not tested. This discrepancy may be due to cellular identity, as limited functional characterization was completed, or the choice of readouts not being suitable for these drug candidates. A follow-up study further examined these top drug candidates, as well as therapeutic mitochondrial transfer, for their ability to mitigate oxidative stress, mitochondrial dysfunction, and ISR dysregulation in these patient-derived astrocytes^291^. Edaravone successfully reduced oxidative stress and ER stress markers, while mitochondrial transfer restored mitochondrial function and reversed VWM-associated proteomic pathway alterations^291^.

### Aicardi-Goutières syndrome

Aicardi-Goutières Syndrome (AGS) is a genetically and clinically heterogenous disease typically characterized by neurological and systemic manifestations. The classic neurological presentation is that of early-onset encephalopathy with calcifications and white matter abnormalities on brain MRI, severe global developmental delay, generalized dystonia, and spastic quadriparesis. Systemic features are common and include skin manifestations such as chilblains, diabetes insipidus, glaucoma, hypothyroidism, and others^309–311^. Chronic overproduction of cytokine type I interferon (IFN) is a ubiquitous finding, labeling this disorder a type I interferonopathy^309^. Nine genes are implicated in AGS etiology, each integral to pathways of nucleic acid metabolism and sensing including *TREX1*, *RNASEH2A*, *RNASEH2B, RNASEH2C, ADAR, SAMHD1*, *IFIH1, LSM11*, and *RNU7-1*^309,312–317^. Mouse models of *Trex1*, *Adar1* and *Rnaseh2b*^318^ have suggested that the elevated IFN response may result from an aberrant response to self-derived nucleic acids and misrecognition of retroelements^319^. Therapies and clinical trials for AGS have primarily explored the use of reverse transcriptase inhibitors (EudraCT Number: 2022-000064-21)^320^ and JAK inhibitors (EudraCT Number: 2015-003424-31), though current treatment strategies are primarily focused on the latter, including baricitinib and ruxolitinib, which have shown promise in recent studies^321–324^.

To date, four AGS iPSC models have been generated, encompassing a variety of AGS-implicated genes, including *TREX1*^325–328^, *RNASEH2B*^326,327^ and *IFIH1*^326^. These models have emphasized the contribution and vulnerability of astrocytes and neurons. Specifically, astrocytes have shown an increase in type I IFN and downstream proinflammatory signatures^325,327^, thought to be mediated via the STING pathway^327^. Further, astrocyte supernatant indicated an enrichment for an inflammatory responses, including the NLRP3 inflammasome, and complement system^327^, which appear to be the primary drivers of neuroinflammation. Though traditionally viewed as an astrocytopathy, AGS-derived neurons exhibited intrinsic vulnerability to toxicity and apoptosis, which was further exacerbated with astrocyte co-culture and exposure to astrocyte conditioned media. Neuronal vulnerability has been further exemplified by AGS-derived 3D organoid models which showed significant neuronal cell death resulting in an overall decrease in organoid size^325,327^.

Recent work utilizing *TREX2* KO ESCs has implicated the role of microglia in disease pathogenesis and provided the first evidence linking microglia dysfunction to the white matter defects associated with AGS. *TREX2* KO microglia were characterized as having a proinflammatory phenotype, together with dysregulated cholesterol biosynthesis and intracellular lipid accumulation, independent of IFN signaling^328^. Defects in cholesterol metabolism is an emerging pathogenic mechanism in AGS, reported in a *samhd1* mutant zebrafish^329^ and patient peripheral blood samples^328,329^, although more characterization is necessary. When these TREX2 KO microglia were co-cultured with control mOrgs, there was a decrease in the number of myelinating oligodendrocytes, thought to result from alterations in the transition from glial restricted progenitor (GRP) to OPCs^328^.

In AGS, disease mechanisms are delineated by two distinct genotype-dependent pathways: DNA accumulation and RNA sensing dysregulation. In iPSC models involving mutations in *TREX1* and *RNASEH2B*, accumulation of cytoplasmic DNA, including micronuclei, is apparent in CNS cells^325,327^. This extranuclear DNA primarily resulted from reverse transcription of L1 elements in the *TREX1* model^325^, while the *RNASEH2B* model reported DNA damage and disrupted R-loop homeostasis in astrocytes^327^, believed to activate the innate immune response. These findings have been previously reported in mouse models^319,330^ and their contribution to neurological manifestations may not be mutually exclusive as treatment with reverse transcriptase inhibitors (RTi) does not ameliorate all clinical symptoms^331^ or benefit all patients^320^. AGS iPSC models with variants in genes causing RNA processing defects have not been reported, though an ESC *ADAR1* KO model has shown aberrant activation of cytoplasmic dsRNA sensors resulting in IFN production and inflammation^332^. Furthermore, despite varying mechanisms behind underlying autoimmunity, all models demonstrated an induction of a complex inflammatory cascade^325,327^. However, the role of elevated IFN in neurotoxicity remains unclear, and evidence suggests that neurotoxic effects can be mitigated by targeting specific inflammatory pathways without altering IFN levels^327^. This implies that while IFN is involved, it may not be the primary driver of neurotoxicity and instead plays a part in a broader inflammatory response.

Potential therapeutic avenues have also been explored in iPSC models, with the most notable involving the use of reverse transcriptase inhibitors (RTi), which are currently in a phase 2 clinical trial (EudraCT Number: 2022-000064-21)^320^. RTi treatment partially rescued phenotypic abnormalities in *TREX1*-deficient neurons^325^, astrocytes^327^, and microglia^328^. Specifically, when *TREX1*-deficient astrocytes were co-cultured with neurons, RTis were only able to improve IFN scores but not other phenotypic abnormalities^327^. Conversely, RTis were unable to ameliorate defects in *RNASEH2B* KO astrocytes cultures^327^, further emphasizing how RTi treatment may not be a uniform solution for AGS. Various immune-modulating therapies (e.g. corticosteroids, azathioprine, intravenous immunoglobulins) have been reported in small case reports and studies^333–337^, yet their clinical evidence remains inconclusive. One study examined potential cytotoxicity effects in stem cells derived from AGS patients with biallelic pathogenic variants in either *TREX1*, *RNASEH2B* and *IFIH1* variants, however, efficacy was not examined, precluding further insights into immune-modulatory therapy in AGS^326^.

### Leuko-axonopathies

Leuko-axonopathies are a group of leukodystrophies that arise from defects in neuron- or axon-specific genes, or in which the primary pathomechanism is related to axonal dysfunction^5^. Examples discussed in this review include TUBB4A-related leukodystrophy and leukoencephalopathy with brainstem and spinal cord involvement and lactate elevation.

### TUBB4A-related leukodystrophy

TUBB4A-related leukodystrophy (TUBB4A-LD), also known as hypomyelination with atrophy of the basal ganglia and cerebellum (H-ABC), is a disorder dominated by extrapyramidal signs, cerebellar ataxia, and spasticity. Its name is derived from the characteristic MRI pattern of hypomyelination along with progressive atrophy of the putamen, caudate nucleus, and cerebellum^338^. De novo pathogenic variants in *TUBB4A* cause TUBB4A-LD^339^. *TUBB4A* encodes a brain-specific microtubule protein β-tubulin-4A^340^. A spectrum of neurological disorders have been linked to variants in *TUBB4A*, including dystonia type 4^340^ and cases of isolated hypomyelination^341^. In vitro^342,343^ and in vivo models^22,344^ have demonstrated that TUBB4A-LD pathogenesis may be attributed to developmental abnormalities in neurons and/or OLs, contingent on the specific mutations and effects on tubulin dynamics and stability. A phase I/II clinical trial (NCT06369974) is scheduled to begin to evaluate the use of ASO therapy in a single participant.

To model TUBB4A-LD, researchers engineered an iPSC line to express a *TUBB4A* variant (p.D249N), with subsequent differentiation into neurons. Microtubule function was studied alongside additional iPSC lines with *TUBB4A* mutations associated with other neurological disorders^345^. Intraorganellar transport, measured via mitochondrial tracking, indicated that transport speed, but not overall quantity, was altered in TUBB4A-LD neurons. Enhanced microtubule incorporation was proposed to facilitate this increased velocity^345^. Other studies have suggested that such acceleration has been associated with neurodegeneration^346^ and may disrupt energy balance and calcium dynamics^347^.

### Leukoencephalopathy with brain stem and spinal cord involvement and lactate elevation

Leukoencephalopathy with brainstem and spinal cord involvement and lactate elevation (LBSL) is a mitochondrial leukoencephalopathy named for its distinctive MRI pattern and magnetic resonance spectroscopy (MRS) abnormalities. It is generally characterized by a gradual progression of cerebellar ataxia and spasticity, accompanied by dorsal column dysfunction^348^, though a wide phenotypic spectrum is appreciated^349^. LBSL is caused by biallelic pathogenic variants in *DARS2*, encoding the mitochondrial-specific aspartyl-tRNA synthetase^350^, and disease mechanisms primarily involve reduced enzymatic activity of aspartate charging^349^. A large proportion of patients harbor a heterozygous splice site mutation in intron 2 on one allele, resulting in leaky expression of the wild-type transcript, as well as an aberrant truncated transcript lacking exon 3^349,351^. Classification of LBSL as a neuronal disorder has been reinforced by *Dars2* mouse models, as conditional deletion in neurons caused a more severe phenotype compared to OL-specific deletions^352,353^. While no commercial therapies or clinical trials are underway for LBSL, ASOs have been proposed as a potential approach to enhance the production of full-length *DARS2* transcripts, thereby restoring protein functionality^351^.

In iPSC studies of LSBL, cOrgs, and neurons have been generated from seven LBSL-iPSC lines, almost all harboring at least one splice site variant^354^. Mature organoids were characterized transcriptionally, revealing downregulation of genes relating to CNS development and neuronal differentiation, further confirmed via functional studies of 2D neuronal cultures, which exhibited reduced neurite outgrowth, decreased *DARS2* expression, and increased aberrant splicing, a finding previously documented in neuronal cell lines^351^. Interestingly, transcriptional profiles varied genotypically, distinguishing patients with one or more missense variants from those with two splice site variants. Patients with missense variants exhibited a downregulation of mRNA metabolic processes and splicing, indicating potential impairments in RNA processing and modification. Conversely, patients with two splice variants showed an upregulation in these pathways, suggesting a compensatory mechanism to mitigate the effects of disrupted splicing. Findings in mouse hematopoietic stem cells (HSCs) have demonstrated a non-canonical function for *Dars2* in regulating splicing factor stability and Dars2 loss led to disrupted splicing of genes associated with metabolism^355^. Thus, iPSC studies have provided the evidence that LBSL pathogenesis may involve non-canonical dysfunction of DARS2, specifically related to its role in splicing.

### Microgliopathies

Leukodystrophies caused by mutations in microglia-related genes, or in which primary microglial dysfunction is a key disease driver, are referred to as microgliopathies^5^. Examples discussed in this review include polycystic lipomembranous osteodysplasia with sclerosing leukoencephalopathy and CSF1R-related leukodystrophy.

### Polycystic lipomembranous osteodysplasia with sclerosing leukoencephalopathy

Polycystic lipomembranous osteodysplasia with sclerosing leukoencephalopathy (PLOSL), or Nasu-Hakola disease (NHD), is an adult-onset leukodystrophy distinguished by bone cysts, frontotemporal presenile dementia, neuropsychiatric comorbidities and demyelination with axonal spheroid accumulation^356–360^. It is caused by biallelic pathogenic variants in *TREM2* or *TYROBP*/*DAP12*^361,362^. Evidence from histopathology and mouse models have suggested that PLOSL is a primary immunological disorder, characterized by activated microglia, neuronal loss, and astrogliosis^363,364^. TREM2 and DAP12 form part of a complex and are expressed exclusively by cells of the myeloid lineage, including microglia and osteoclasts, partly explaining the pleiotropic phenotype of PLOSL. Various functions of these proteins and disease modeling in *Trem2* and *Dap12* deficient mouse models have revealed diverse, occasionally conflicting, phenotypes and mechanisms^365,366^, necessitating evidence from relevant patient samples to examine disease mechanisms.

Three studies that utilized iPSCs from individuals with homozygous *TREM2* variants revealed that the overall pathogenesis stems from a loss of TREM2 function, ultimately impacting normal functioning of microglia. Consistent with this, TREM2 production, membrane trafficking, and proteolysis were decreased in all patient lines^367–369^ along with reductions in immune activation and antigen presentation markers, aligning with a homeostatic microglia profile^369^. Functionally, these the rate of cell death for these microglia increased upon growth factor withdrawal, coupled with impaired migration, substrate-specific phagocytic defects^368^, and diminished engulfment of apoptotic neuronal cells^367–369^. Cytokine secretion varied across studies, with some showing normal levels^367,368^ while others showing an increase following lipopolysaccharide (LPS) stimulation^369^. Discrepancies may stem from experimental setup, as previous studies indicated that cytokine production was delayed but reached comparable levels after 72 hours^370^. Both PLOSL human brain tissue and mouse models display inflammation and immune cell activation^365,366,369,371–373^, implying that microglia acquire pathogenic inflammatory profiles during the disease course. Lysosomal dysfunction has also been linked to PLOSL^374,375^, where iPSC-derived microglia show reduced endolysosomal gene expression, coupled with compromised vesicle acidification and downregulation of cholesterol genes^369^. Interestingly, interventions to improve lysosomal biogenesis corrected some of the microglial abnormalities^369^, indicating that lysosomal dysfunction may be a central factor in microglia dysfunction. However, the full implications of this treatment needs further exploration.

### CSF1R-related leukoencephalopathy

CSF1R-related leukoencephalopathy (CSF1R-LD) is a progressive disorder characterized by a specific constellation of neuropathologic findings including pigmented glia, neuroaxonal spheroids, and demyelination. Typically emerging in mid to late adulthood, CSF1R-LD presents with neuropsychiatric features followed by progressive motor and cognitive impairment^376,377^. CSF1R-LD is also commonly known as adult-onset leukoencephalopathy with axonal spheroids and pigmented glia (ALSP) and formally unifies two previously distinct conditions, hereditary diffuse leukoencephalopathy with spheroids (HDLS) and pigmentary orthochromatic leukodystrophy (POLD), under a single diagnostic umbrella due to their shared genetic basis and overlapping clinical features^378^. Autosomal dominant pathogenic variants in the colony-stimulating factor 1 receptor (*CSF1R*) gene, a microglial cytokine receptor, are causative^379^. Very little is known about the underlying disease mechanisms. While zebrafish^380^ and mouse models^381–384^ suggest primary microglial dysfunction, the underlying mechanisms appear inconsistent across studies, highlighting the need for further investigation. HSCT offers the potential to provide clinically meaningful stabilization of disease progression in some individuals^385,386^, however, clear guidelines are needed to define its role in disease management and identify which individuals are most likely to benefit for optimal outcomes. To address this, a clinical trial evaluating the efficacy of HSCT for CSF1R-LD is ongoing (NCT04503213).

Three CSF1R-LD iPSC lines have been generated for disease modeling, all focusing on differentiation to microglia^387–389^. Differentiation of CSF1R-LD iPSCs to microglia using standard protocols^390,391^ has proven challenging, with some studies reporting unsuccessful generation of patient-derived microglia^387^ or producing sub-confluent, non-proliferative cultures^388^. To address these limitations, one study developed a novel microglial differentiation protocol, achieving an increased yield and enhanced primary microglial identity in iPSC-derived cells compared to earlier methods^387^. Despite methodological improvements, CSF1R-LD iPSCs still presented with reduced numbers of microglia, consistent with other iPSC studies^388^ further mirroring findings in CSF1R-LD human brain tissue and zebrafish modeling^380^, though the cause of this decrease is unclear. In contrast, mouse models displayed increased or unaltered microglia density^381,382^. Loss of canonical CSF1R function was evident in microglia, with reduced cell surface expression and decreased autophosphorylation without changes in transcript or protein levels, suggesting defects in CSF1R trafficking as previously demonstrated^392^. At the cellular level, CSF1R-LD microglia displayed migration defects and a reduction in P2RY12, an important receptor for the chemotactic movement of microglia toward dead cells, indicating compromised chemotaxis. Phagocytic ability was enhanced across various substrates in CSF1R-LD microglia, along with elevated lysosomal storage, despite no increase in lysosomal activity^387^. It is unknown how increased phagocytosis may contribute to CSF1R-LD, though this has been reported in *Csf1r*^+/-^ mice^383^.

To further examine these cells in vivo, one study injected CSF1R-LD and isogenic control microglia into a mouse model of CSF1R-LD^388^. Isogenic microglia were able to successfully engraft, distribute throughout the brain, and ameliorate disease pathology. In contrast, patient-derived microglia failed to correct the disease, and were predominantly localized near the injection site. While in vitro experiments showed reduced proliferative capacity of CSF1R-LD microglia compared to its isogenic, in vivo, patient-derived microglia showed no hypoproliferative defects six weeks post-injection. Though delayed proliferation was proposed as a potential cause of the engraftment defects^388^, it is also plausible that migration is impaired in this patient-derived cell line, although this was not directly assessed. Supporting this hypothesis, reduced expression of P2RY12 was also observed in these cells. Future studies are needed to investigate migratory defects in greater detail to determine their potential role in the pathogenesis of CSF1R-LD.

The preclinical studies supporting the recently terminated clinical trial of iluzanebart (NCT05677659) relied almost exclusively on evidence from in vitro models, including iPSC derived microglia, marking a significant milestone in the use of iPSC models for preclinical development in leukodystrophies^389^. Specifically, treatment with iluzanbart in microglial cultures edited to carry the common p.I794T mutation in *CSF1R* restored CSF1R surface levels and increased cell viability, providing the first evidence that targeting TREM2 receptors can functionally compensate for CSF1R deficiency^389^.

### Leuko-vasculopathies

Leuko-vasculopathies, also referred to as leuko-microangiopathies, are leukodystrophies that arise from cerebral small vessel pathology^5^. Prominent examples discussed in this review include cerebral autosomal dominant arteriopathy with subcortical infarcts and leukoencephalopathy and leukoencephalopathy with calcifications and cysts.

### Cerebral autosomal dominant arteriopathy with subcortical infarcts and leukoencephalopathy

Cerebral autosomal dominant arteriopathy with subcortical infarcts and leukoencephalopathy (CADASIL) is a cerebral small vessel disease caused by heterozygous pathogenic variants in *NOTCH3*^393^. The disease typically presents during the third or fourth decade of life with aura-associated migraines progressing to psychiatric symptoms, ischemic strokes, dementia, and motor impairment, with prominent white matter lesions, cerebral microbleeds, and lacunar infarcts on brain MRI^394,395^. Most patients exhibit granular osmiophilic material, a diagnostic and pathological hallmark, on the surface of vascular smooth muscle cells (VSMCs) and pericytes, along with NOTCH3 accumulation, VSMC degeneration, and increased thickening of small blood vessel walls^396,397^. NOTCH3, predominantly expressed in VSMCs and pericytes, is a transmembrane signaling receptor involved in cell fate and development^397^. Pathogenic mechanisms underlying NOTCH3 variants and CADASIL are still under investigation, as altered signaling has only been observed with some mutations^398,399^. Therapeutic avenues are primarily supportive, with a clinical trial underway to evaluate the efficacy of lomerizine hydrochloride in reducing the risk of recurrent cerebral ischemic events (jRCTs051220072)^400,401^.

CADASIL iPSC disease modeling has concentrated on investigating capillary and vessel formation using endothelial and mural cells, a broad cellular category including both VSMCs and pericytes. Findings in mural cells and vascular organoids have recapitulated common pathogenic hallmarks of CADASIL such as NOTCH3 extracellular domain deposits^402–404^, and confirmed previous reports of cytoskeletal structure abnormalities marked by irregular F-actin branching and distribution^402,403,405–407^. Investigation of PDGFRβ expression, cell proliferation, and migration have varied among studies due to varying culture conditions and protocols. Specifically, VSMC cultures have shown increased proliferation^406^ and PDGFRβ levels^402^, without differences in migration^406^. Conversely, broader mural cell cultures have reported conflicting PDGFRβ expression^407,408^, faster migration^407^, and no changes in proliferation^407^. The observed discrepancies in proliferation compared to documented hypo-proliferation in mouse and human VSMCs^409–411^ could be influenced by factors such as mitogen levels, source of VSMCs, and differing stages of disease progression. In contrast, vascular organoids containing mural and endothelial cells showed decreased PDGFRβ expression and either reduced^412^ or no changes in proliferation^403^. RNA sequencing of VSMCs has supported phenotypic observations of cytoskeletal disorganization and abnormal proliferation but also implicated upregulation of the NF-kB inflammatory pathway, suggesting multifaceted disease mechanisms^406^. Further, proteomic analysis of CADASIL iPSCs also highlighted dysregulated pathways related to cytoskeleton formation and cell adhesion, among others^404^.

In CADASIL iPSC-derived cells, functional examination of capillary tubule formation showed compromised capillary stability over time, resulting from a progressive decrease in MC coverage at the tubule^403,408^. Though deterioration of the smooth muscle layer is a well-documented finding, iPSC models indicated MC sensitivity to apoptosis only under stress^408^ and derived endothelial cells displayed increased apoptosis when cultured in the presence of CADASIL-derived MCs. Increased cell death was further confirmed in blood vessel organoids^403,412^. Vascular endothelial growth factor (VEGF) secretion by MCs was identified as a critical factor influencing endothelial survival, with VEGF supplementation and NOTCH3 siRNA-mediated knockdown effectively restoring capillary stability^408^. Sequencing of CADASIL-derived blood vessel organoids demonstrated abnormalities in vessel functioning, with differentially expressed genes relating to cell adhesion, extracellular matrix organization, and vessel development, while KEGG pathway analysis further implicated altered inflammatory pathways in disease pathogenesis^402,412^. Drug screening in these organoids demonstrated that ROCK inhibition helped partially improve the interaction between mural and endothelial cells^403^.

Recent studies have attempted to model the NVU, as the BBB is frequently implicated in other cerebral small vessel diseases^413–415^, and reduced cerebral blood flow and BBB leakage have been reported in CADASIL patients and mouse models^416,417^. Here, the NVU was established using a combination of iPSC-derived MCs, BMECs, astrocytes, and neurons, where BBB integrity and permeability were impacted due to intrinsic defects in BMECs and MCs contributing to reduced barrier function and mislocalization of some tight junction proteins^418^.

### Leukoencephalopathy with calcifications and cysts

Leukoencephalopathy with calcifications and cysts (LCC), also known as Labrune syndrome, is a progressive microangiopathy associated with biallelic variants in *SNORD118* encoding the box C/D small nucleolar RNA (snoRNA) U8. U8 snoRNA plays an important role in ribosomal RNA (rRNA) maturation, classifying this disorder within the umbrella of the ribosomopathies, a heterogenous group of diseases arising due to abnormalities in the ribosome biogenesis process. As its name implies, LCC is associated with a distinctive MRI triad of diffuse cerebral white matter abnormalities, cerebral calcifications, and parenchymal cysts^419^. Although disease onset and course are variable, LCC typically presents in childhood with developmental delay and progressive neurological and cognitive decline. It is often accompanied by epilepsy and variable neurological signs depending partially on the location and size of the cysts^420^. Symptomatic management can provide temporary relief of some clinical symptoms through the use of surgical intervention to drain cysts. A few cases have been treated with Bevacizumab, a VEGF inhibitor, showing apparent beneficial effects^421–423^, although the variable disease course and the possibility of spontaneous improvement^424^ highlight the importance of considering these case reports with caution. To thoroughly assess its efficacy in this highly variable disease, delineation of its natural history as well as further studies involving a larger patient cohort and ideally using a double-blind methodology are necessary.

iPSC models have been developed modeling both neurological and non-neurological features^425,426^. In both cases, CRISPR-Cas9 genome editing was utilized to generate either heterozygous *SNORD118* knock-out iPSC lines (as homozygous KOs were non-viable)^425,426^ or homozygous iPSC lines with the common *5 C > G point mutation^425,427^. cOrgs differentiated from both the heterozygous KO and homozygous *5 C > G iPSCs were significantly smaller than controls, due to a loss of proliferative ability leading to decreased numbers of NPCs and thereby post-mitotic neurons, modeling microcephaly seen in LCC. Mechanistically, this microcephalic-like phenotype was found to be due to increased p53 signaling activation, leading to both cell cycle defects and apoptosis in NPCs^425,427^. Similar findings of reduced cell proliferation, aberrant p53 signaling and increased cell death were also seen in neural crest organoids (ncOrg)^427^. Further studies of the 5*C > G mutant NPCs revealed impaired rRNA synthesis and maturation^425^, defective ribosome biogenesis^425,427^, decreased protein synthesis^425,427^, proteotoxic stress^427^ and aberrant ISR responses^425^. Moreover, nucleolar defects were identified as a central cause of these pathologies, characterized as abnormalities in nucleolar morphology due to impaired liquid-liquid phase separation (LLPS) in specific nucleolar regions such as the dense fibrillar component (DFC) and the granular component (GC), together with reduced expression and function of nucleolar chaperone FBL^427^. Treatment of cerebral organoids with 2BAct, a small eIF2B activator that acts on the ISR, partially rescued proliferative defects increasing the numbers of NPCs and neurons^425^. Similarly, overexpression of full-length FBL in mutant organoids restored nucleolar morphology, ribosome biogenesis and neuronal differentiation, demonstrating that LLPS nucleolar function play a critical role in disease pathogenesis^427^. Other studies have focused on modeling the effect of heterozygous *SNORD118* KO on cranial suture formation and fusion^426^. However, to our knowledge, no reports of craniosynostosis has been reported in LCC. Though not representative of the genetic underpinnings of LCC, heterozygous iPSC lines showed reduced expression of U8 RNA and exhibited defects in MSC proliferation and subsequent premature osteogenic differentiation. Ribosomal profiling showed decreased expression of genes involved in the complement pathway and those encoding ribosomal proteins, suggesting altered ribosome biogenesis function^426^.

### Stem cell models to advance leukodystrophy therapeutics

Though leukodystrophies have been described in the literature for nearly a century^428^, few disease modifying therapies are widely available. Of these, most are restricted to early intervention and specific disease subtypes and serve to slow disease progression. Although more clinical trials and disease-modifying therapies are available now than ever before, they are predominantly restricted to classical and historically well-studied leukodystrophies. However, most diseases and disease forms remain without therapeutic options. This translational gap can be attributed partly to a lack of appropriate disease models recapitulating white matter pathology, thereby limiting insights into disease pathogenesis and subsequent development of therapeutics. Stem cells provide scalable personalized disease models that facilitate high-throughput drug screening and offer a human-relevant system for the development and testing of therapies. In this review, we have highlighted emerging areas of stem cell-based therapeutics, including AAV serotype testing, drug screening, and cell therapy. Transplantation of allogenic or genetically corrected autologous neural lineage cells, including NPCs and OPCs, shows potential in replacing CNS cells in preclinical studies of neurological disorders, including CD, MLD, and PMD. However, initial human applications have shown minimal success and carry risks associated with the procedure, immunosuppression, and potential malignancy. To date, only one cell therapy clinical trial for PMD (NCT01005004) has been conducted, using allogenic fetal NPCs, and while transplantation safety was demonstrated, clinical and biomarker outcomes did not substantially improve, leading to study dissolution^429,430^. However, preclinical studies in animal models have yielded promising results using either NPCs or glial progenitor cells^431–436^, suggesting that cell transplantation may become a viable therapeutic avenue.

### Strategies to enhance reliability and clinical relevance of stem cell models in leukodystrophy research

As discussed in this review, stem cell research has significantly advanced our understanding of leukodystrophies, offering unique insights into their complex pathologies. Despite this progress, inherent variability in stem cell studies highlights the urgent need for enhanced quality control and standardization. These variations arise from varying culture techniques between research groups and handling of cell lines, distinct differentiation protocols, and individual stem cell line differences that often arise from prolonged passaging. To mitigate these issues and improve reproducibility, it is vital to standardize experimental setup where possible and ensure meticulous reporting of experimental conditions. While most leukodystrophy stem cell modeling studies have adhered to established reporting guidelines, not all studies reviewed here consistently verified or reported cellular identity before conducting sensitive disease-relevant assays. Including this information will greatly help the field robustly compare studies. To reduce intra-study variability when using iPSCs, isogenic controls can be generated using gene editing technology to correct patient-specific mutations, allowing for parallel analysis to delineate the effect of the pathogenic variant(s) in cells carrying the same genetic background. In the study of rare and ultra-rare diseases, such as leukodystrophies, isogenic controls can help increase the statistical power of the cell-specific assays. However, few leukodystrophy studies have employed isogenic controls (19.05% n = 24) and those utilizing healthy controls often use an insufficient number of lines (i.e., n = 1), limiting definitive conclusions. Another notable challenge is the lack of detailed clinical characterization on the patient from which iPSCs are derived, which significantly limits the translational value of the research. The reporting of both the genotype and phenotype of the individual from whom the iPSCs are derived (or in the case of engineered mutations, which phenotype the mutation typically corresponds to), is arguably one of the most critical aspects of iPSC-based studies. This is especially true for leukodystrophies, which are highly heterogeneous disorders associated with a wide range of clinical subtypes and presentations. Some studies included within this review failed to report patient genotypes (3.093%, n = 6), and the majority provided only a general clinical diagnosis (89.69%, n = 174), omitting specific subtypes or patient presentations associated with the derived iPSCs that may help to clarify similarities and differences in findings among studies. This lack of detailed characterization could contribute to the variability observed in iPSC studies. Indeed, variability may not necessarily stem from experimental methodologies but rather reflect distinct pathogenic mechanisms between individuals with varied clinical presentations. Though it is tempting to speculate that a single disease entity is unified by a singular pathogenic mechanism, the diverse clinical presentations associated with distinct leukodystrophies suggest that different clinical subtypes may correspond to a different pathophysiology. This implies that variability in findings could reflect true biological differences rather than technical inconsistencies. Without a thorough reporting of genotype and phenotype, it becomes challenging to discern whether observed differences are methodological artifacts or a genuine biological variability. Most importantly, research must be informed by clinical insights to ensure the relevance of identified stem cell phenotypes to actual disease mechanisms. This focus helps in distinguishing phenotypes truly indicative of the disease to avoid identifying an overwhelming number of non-specific and incidental findings.

### Reprogramming and differentiation protocols in leukodystrophy modeling

A wide range of somatic cell types have been successfully used to generate iPSCs for leukodystrophy modeling, most commonly fibroblasts and PBMCs. When rigorous quality control checks are in place, the source cell type has little effect on iPSC characteristics, though it can impact how efficiently reprogramming occurs^437^. For instance, more differentiated cells, like fibroblasts, generally reprogram less efficiently than more proliferative, less lineage-committed cells such as CD34+ hematopoetic stem cells^438^. These differences likely reflect intrinsic variations in chromatin structure, proliferative capacity, and responsiveness to reprogramming factors^438^. Further, during reprogramming, much of the cell’s epigenetic landscape is reset, much of the epigenetic landscape is reset, including widespread erasure of DNA methylation status, which minimizes the influence of the source cell’s epigenetic memory on iPSC behavior^439^. Reprogramming methods have also evolved over time. Earlier studies often used integrating viral vectors such as retroviral and lentiviral systems, which pose risks of insertional mutagenesis and genomic instability^440^. More recent approaches favor non-integrating strategies, such as Sendai virus and mRNA-based transfection, which have become increasingly popular in leukodystrophy modeling^441–443^. Despite these differences in both somatic cell sources and reprogramming methods, downstream variation in disease modeling outcomes is more often driven by the differentiation protocol used or experimental design than by the initial reprogramming itself.

Various differentiation protocols were used to derive over 24 different cell types for leukodystrophy disease modeling, contributing to extensive variability across studies. The most commonly used protocols included Chambers et al.^444^ for NPCs (13.15%, n = 5/38), Shi et al.^445^ for neurons (12.50%, n = 5/40), Canals et al.^271^ for astrocytes (21.43%, n = 6/28), Douvaras and Fossati^104^ for oligodendroglial lineage cells (29.41%, n = 5/17), van Wilgenburg et al.^446^ for microglia (33.33%, n = 3/9), and Lancaster et al.^447^ for cortical organoids (50.00%, n = 8/16). Among the 27 studies utilizing 3D modeling, most followed guided differentiation protocols using small molecules (n = 18), rather than spontaneous unguided differentiation methods (n = 9). An emerging approach in 2D stem cell differentiation involves overexpression-based protocols, which offer faster differentiation times and produce more homogeneous cell populations. In total, 12 studies (9.524%) utilized overexpression protocols, mainly for neuronal^448^ or astrocyte^271^ differentiation. While advantageous for certain applications and end-stage phenotyping of mature cells, overexpression may be less suitable for modeling disorders where neurodevelopment may be affected, as it imposes a cellular identity rather than allowing growth-factor-based differentiation to better mimic natural developmental processes.

Given the relative ease and plethora of differentiation protocols to generate NPCs, neurons, and astrocytes, the majority of iPSC modeling has focused on these cell types (Fig. 4). Though oligodendrocytes have been generated in a number of disease models reviewed here, many of the protocols utilized failed to generate terminally mature cells, focusing their assays on the oligodendrocyte progenitor cells (OPCs) or early oligodendrocytes (O4+ cells), precluding insights into oligodendrocytes and myelination. Moreover, despite the primary impact of leukodystrophies on white matter, there is a noticeable scarcity of studies that employ co-culture or 3D systems including OPCs and OLs. Such models are crucial for accurately simulating white matter pathology. Historically, the efficiency of oligodendroglial differentiation protocols has been a limiting factor; however, recent improvements and publications of more effective protocols offer promising avenues for future research^449,450^.

## Discussion

Since the introduction of the first leukodystrophy stem cell models in 2011, the field has witnessed a significant expansion in the development of this technology (Fig. 2). Stem cell models have been developed for over 41 leukodystrophies, focusing predominantly on the more prevalent diseases. Insights from these models have led to significant advances in our understanding of leukodystrophies, including reinforcing previous knowledge gained from rodent models and human-immortalized cell lines, permitting new insights into disease mechanisms, and providing a relevant platform for the development and evaluation of therapeutics. However, we also identified several key limitations of current modeling approaches, including the lack of reported clinical information on derived iPSCs and variability in methodology and findings between studies. Improved reporting of clinical and methodological data will be essential to improve reproducibility and standardization across the highly heterogenous group of disorders, ensuring that stem cell-based modeling continues to serve as a reliable and scalable tool for understanding leukodystrophies.

Leukodystrophy modeling is steadily expanding beyond traditional CNS cell types to better reflect the complexity of these disorders and support ongoing therapeutic development. While most studies to date have focused on differentiation to neurons, astrocytes, and oligodendrocyte lineage cells, recent advances in differentiation protocols have enabled the generation of more specialized cell types, such as the use of BMECs to model the blood-brain barrier. In parallel, the integration of 3D systems and co-culture approaches have allowed researchers to examine more complex pathophysiological processes, including cell-cell interactions and tissue architecture. Further, as many leukodystrophies present with non-neurological features, we anticipate increasing interest in modeling these phenotypes. A handful of studies included in this review have begun to address these aspects, using neural crest cells to model craniofacial abnormalities, retinal organoids for ocular anomalies, and chondrocytes and osteoblasts to model skeletal involvement, among others. Lastly, stem cell-based models also hold immense promise for therapeutic development. While therapeutic testing has so far been applied in a limited number of leukodystrophy models, we expect a growing shift towards the use of these systems for therapeutic screening and evaluation. The ability to assess therapeutic efficacy in human-relevant models will be essential for bridging the gap between preclinical findings and clinical translation.

Further, while stem cell models have provided important insights into leukodystrophy biology, it remains useful to use them alongside other disease modeling approaches to validate findings and build a more complete understanding of disease mechanisms. In particular, in vivo models offer the advantages of capturing systemic interactions, multi-cellular dynamics, and longitudinal disease progression that can be more difficult to replicate in vitro. Conversely, stem cell systems enable precise manipulation and mechanistic studies in human-derived cell types that are otherwise inaccessible or behave differently in animal models. The choice between models should be guided by the specific research goal, whether the aim is to dissect cell-autonomous mechanisms, test therapeutic interventions, or study disease progression in a whole-organism context. If possible, using both models in tandem can bridge the gap between cellular level insight and physiological relevance, offering a more complete picture of leukodystrophy pathology.

This scoping review is associated with some limitations inherent to the review process and study scope. First, the search was limited to three databases, and only articles published in English were included, which may have led to the omission of relevant studies. Inherent variability in study designs, stem cell sources, differentiation protocols, and phenotypic outcomes also posed challenges for direct comparisons across studies. Finally, the evolving definition of leukodystrophies presented additional limitations to conducting this review. Although we followed the most recent terminology guidelines and adopted an inclusive approach, it is possible that relevant disorders were missed, particularly if they were not explicitly labeled as a leukodystrophy or leukoencephalopathy, or if they were not captured in our extensive gene list, which was based on previously published literature.

In conclusion, this review highlights the versatility and utility of leukodystrophy disease modeling using stem cells. Continued development and refinement of existing models, along with the generation of new models for ultra-rare and newly described disorders, will be essential for capturing the full spectrum of leukodystrophy pathology. Moving forward, the field could greatly benefit from the inclusion of a broader array of patient-derived iPSC lines and the consideration of various disease presentations to further enhance our ability to model these complex disorders and develop effective therapeutic strategies.

## Methods

This scoping review was conducted to systematically identify, categorize, and analyze the relevant literature to provide a comprehensive overview of existing stem cell models and their applications in leukodystrophy research.

### Search strategy

Methodological framework proposed by Arksey and O’Malley^451,452^ and refined by the Joanna Briggs Institute (JBI)^453,454^ were followed and results were reported in accordance with the PRISMA-ScR (Preferred Reporting Items for Systematic reviews and Meta-Analyses extension for Scoping Reviews) guidelines^455^. A comprehensive search was implemented across three separate electronic databases, including Ovid MEDLINE, Ovid Embase and Scopus (Elsevier), for articles published from inception to April 2025. The search strategy incorporated a combination of keywords and Medical Subject Headings (MeSH) terms associated with stem cells and leukodystrophies (Supplementary Table 1). Articles retrieved were subjected to title, abstract, and full-text screening based on a set of predetermined inclusion criteria by two independent reviewers. Inter-rater reliability statistics were calculated to assess the level of agreement between reviewers during the screening process^456^. Disagreements were resolved through discussion. Following selection, relevant data was charted from included studies using a standardized form developed by our team, capturing study characteristics, disease type/subtype, reported pathogenic variants, stem cell type and source, differentiation strategy, and modeled phenotypes. The results were summarized in tabular format and supplemented by narrative synthesis to identify insights, gaps, and research trends among studies.

### Inclusion and exclusion criteria

The criteria for inclusion required published articles that utilized human-derived stem cells to model a leukodystrophy. For the search, a broad definition of leukodystrophies was applied, encompassing genetic white matter disorders that have been previously classified as leukodystrophies in published literature^1,3,5,457,458^ or listed as such in the Online Mendelian Inheritance in Man (OMIM) database. Both patient-derived iPSCs and stem cells genetically engineered to carry leukodystrophy-associated mutations were included. Additionally, knockout stem cell lines for genes known to cause leukodystrophies through loss-of-function mechanisms were considered eligible. For disorders with high allelic heterogeneity, only studies that included stem cells derived from individuals diagnosed with leukodystrophy or carrying confirmed leukodystrophy-causative genetic variants were included. Studies utilizing stem cells from individuals with uncertain pathogenic variants or no white matter abnormalities on brain MRI were excluded. Stem cells derived from rodents and other model organisms were excluded. Preclinical cell therapy studies were excluded, unless the stem cells utilized were patient derived. Reviews, preprints, and conference abstracts that did not provide sufficient methodological detail or original experimental data were excluded.

## Supplementary information

Chapleau et al. Supplementary Information.

PRISMA-ScR-Fillable-Checklist_10Sept2019.

## Data Availability

All data generated in this study are included within this published article and the supplementary files.
