# Supplementary material for: A scoping review of stem cell models of leukodystrophies: advances in understanding pathophysiological mechanisms
Source: NPJ Genom Med. 2025 Nov 28;10:77. doi: 10.1038/s41525-025-00533-0 (PMC12663344; doi:10.1038/s41525-025-00533-0)
Supplement: Supplementary file 1 — Chapleau et al. Supplementary Information. [file 41525_2025_533_MOESM1_ESM.pdf]

Chapleau et al. Supplementary Information

Supplementary Table 1. iPSC search strategy for OVID, EMBASE and SCOPUS with records retrieved.

Search Concept	Search Terms	Records Retrieved
**Ovid Medline**		
Human (1)	exp Humans/ or Human*.mp. or "Human Physiology".mp. or "Homo sapiens".mp. or "Human Cells".mp. or "Human-Derived".mp. or Patient*.mp.	24893987
iPSCs (2)	exp Induced Pluripotent Stem Cells/ or "Pluripotent Stem Cell*".mp. or "Stem Cell Model*".mp. or iPSC*.mp. or iPS.mp.	58577
Leukodystrophies (3)	leukodystroph*.mp. OR leukoencephalopath*.mp. OR "white matter dis*".mp. OR hypomyelinat*.mp. OR dysmyelinat*.mp. OR demyelinate*.mp. OR "myelin vacuolization".mp. OR "myelin dis*".mp. OR leuko-axonopath*.mp. OR astrocytopath*.mp. OR microgliopath*.mp. OR leuko-vasculopath*.mp. OR exp Adrenoleukodystrophy/ OR Adrenoleukodystrophy.mp. OR ALD.mp. OR adrenomyeloneuropathy.mp. OR AMN.mp. OR ccALD.mp. OR acALD.mp. OR ABCD1.mp. OR X-ALD.mp. OR exp Metachromatic Leukodystrophy/ OR MLD.mp. OR ARSA.mp. OR SapB.mp. OR "Arylsulfatase A".mp. OR "Saposin B".mp. OR "Metachromatic Leukoencephalopathy".mp. OR "Diffuse Cerebral Sclerosis, Metachromatic Form".mp. OR "Sulfatide Lipidosis".mp. OR "Arylsulfatase A Deficiency".mp. OR "ARSA Deficiency".mp. OR "Cerebroside Sulfatase Deficiency".mp. OR MLDSAPB.mp. OR "Saposin B Deficiency".mp. OR PSAP.mp. OR Prosaposin.mp. OR Krabbe.mp. OR "Globoid cell leukodystrophy".mp. OR GALC.mp. OR galactocerebrosidase.mp. OR galactosylceramidase.mp. OR "Cerebrotendinous xanthomatosis".mp. OR CTX.mp. OR CYP27A1.mp. OR "Pelizaeus-Merzbacher disease".mp. OR PMD.mp. OR "hypomyelination of early myelinating structures".mp. OR HEMS.mp. OR "PLP1-null syndrome".mp. OR "spastic paraplegia type 2".mp. OR SPG2.mp. OR PLP1.mp. OR HLD1.mp. OR "POLR3-related leukodystrophy".mp. OR POLR3-HLD.mp. OR 4H.mp. OR "RNA polymerase III".mp. OR POLR3A.mp. OR POLR3B.mp. OR POLR1C.mp. OR POLR3D.mp. OR POLR3K.mp. OR HLD7.mp. OR HLD8.mp. OR HLD11.mp. OR HLD21.mp. OR "Tremor Ataxia with Central Hypomyelination".mp. OR TACH.mp. OR ADDH.mp. OR "Ataxia Delayed Dentition and Hypomyelination".mp. OR "4H syndrome".mp. OR HCAHC.mp. OR "hypomyelination with cerebellar atrophy and hypoplasia of the corpus callosum".mp. OR Canavan*.mp. OR aspartoacylase.mp. OR ASPA.mp. OR Alexander*.mp. OR AxD.mp. OR ALXDRD.mp. OR "Vanishing white matter".mp. OR VWM.mp OR "childhood ataxia with central hypomyelination".mp. OR CACH.mp. OR eIF2B.mp. OR EIF2B1.mp. OR EIF2B2.mp OR EIF2B3.mp OR EIF2B4.mp. OR EIF2B5.mp. OR	449998

	"Cree Leukoencephalopathy".mp. OR "Aicardi-Goutières syndrome".mp. OR AGS.mp. OR TREX1.mp. or RNASEH2A.mp. OR RNASEH2B.mp. OR RNASEH2C.mp. OR ADAR.mp. OR SAMHD1.mp. OR IFIH1.mp. OR LSM11.mp. OR RNU7-1.mp. OR "Cree encephalitis".mp. OR "Cerebral leukodystrophy with retinal vasculopathy".mp. OR "Oculodentodigital dysplasia".mp. OR ODDD.mp. OR "Hypomyelination with atrophy of the basal ganglia and cerebellum".mp. OR "H-ABC".mp. OR "TUBB4A".mp. OR "B-tubulin-4A".mp. OR "TUBB4A-related".mp. OR "HLD6".mp. OR "UMF1".mp. OR "HLD14".mp. OR "Leukoencephalopathy with brain stem and spinal cord involvement and lactate elevation".mp. OR "LBSL".mp. OR "DARS2".mp. OR "aspartyl-tRNA synthetase".mp. OR "mt-AspRS".mp. OR "Nasu-Hakola disease".mp. OR "NHD".mp. OR "polycystic lipomembranous osteodysplasia with sclerosing leukoencephalopathy".mp. OR "PLOSL".mp. OR "Adult-onset leukoencephalopathy with axonal spheroids".mp. OR "ALSP".mp. OR "hereditary diffuse leukoencephalopathy with spheroids".mp. OR "HDLS".mp. OR "pigmentary orthochromatic leukodystrophy".mp. OR "POLD".mp. OR "CSF1R".mp. OR "CSF1R-related".mp. OR "brain abnormalities, neurodegeneration, and dysosteosclerosis".mp. OR "BANDDOS".mp. OR "Cerebral autosomal dominant arteriopathy with subcortical infarcts and leukoencephalopathy".mp. OR "CADASIL".mp. OR "NOTCH3".mp. OR "Leukoencephalopathy with calcifications and cysts".mp. OR "LCC".mp. OR "U8".mp. OR "SNORD118".mp. OR "Labrune syndrome".mp. OR "CARASAL".mp. OR "Cathepsin-A related arteriopathy with strokes and leukoencephalopathy".mp. OR CTSA.mp. OR "Waardenburg-Hirschsprung".mp. OR "PCWH".mp. OR "Waardenburg syndrome".mp. OR "SOX10-related".mp. OR "Multiple sulfatase deficiency".mp. OR "MSD".mp. OR "SUMF1".mp. OR "PMD-like".mp. OR "Pelizaeus-Merzbacher-like disease".mp. OR "Cx47-related".mp. OR "Cx47".mp. OR "AHDS".mp. OR "Allan-Herndon-Dudley syndrome".mp. OR "SLC16A2".mp. OR "MCT8".mp. OR "Hypomyelination with congenital cataract*".mp. OR "HCC".mp. OR "FAM126A".mp. OR "HYCC1".mp. OR "DRCTNNB1A".mp. OR "HLD5".mp. OR "Hypomyelination with spondylometaphyseal dysplasia".mp. OR "AIFM1".mp. OR "PDCD8".mp. OR "Spondyloepimetaphyseal dysplasia with hypomyelinating leukodystrophy".mp. OR "SEMDHL".mp. OR "Spondyloepimetaphyseal dysplasia, X-linked, with hypomyelinating leukodystrophy".mp. OR "leukoencephalopahy with metaphyseal chondrodysplasia".mp. OR "LKMCD".mp. OR "spondyloepimetaphyseal dysplasia, x-linked, with mental deterioration".mp. OR "Megalencephalic leukoencephalopathy with subcortical cysts".mp. OR MLC.mp. OR MLC1.mp. OR MLC2.mp. OR HEPACAM.mp. OR GLIALCAM.mp. OR AQP4.mp. OR GPRC5B.mp. OR "Gangliosidosis".mp. OR "GM1".mp. OR "beta-galactosidase-1 deficiency".mp. OR "GLB1".mp. OR "generalized gangliosidosis".mp. OR "GLB1 deficiency".mp. OR "GM2".mp. OR "Tay-Sachs".mp. OR	

	"GM2-gangliosidosis type I".mp. OR "B variant GM2-gangliosidosis".mp. OR "hexosaminidase A deficiency".mp. OR "HexA".mp. OR "GM2A".mp. OR "Sandhoff".mp. OR "GM2-gangliosidosis type II".mp. OR "hexosaminidases A and B deficiency".mp. OR "HexB".mp. OR "AIMP1-related".mp. OR "AIMP1".mp. OR "EMAP2".mp. OR "EMAPII".mp. OR "HLD3".mp. OR "AIMP2-related".mp. OR "AIMP2".mp. OR "HLD17".mp. OR "HSPD1-related".mp. OR "HSPD1".mp. OR "HSP60".mp. OR "HLD4".mp. OR "MitCHAP60".mp. OR "CPN60".mp. OR "mitochondrial HSP60 chaperonopathy".mp. OR "Cerebral autosomal recessive arteriopathy with subcortical infarcts and leukoencephalopathy".mp. OR "cerebral AR arteriopathy with subcortical infarcts and leukoencephalopathy".mp. OR "CARASIL".mp. OR "HTRA1-related cerebral small vessel disease".mp. OR "HTRA1".mp. OR "Adult-onset autosomal dominant leukodystrophy".mp. OR "ADLD".mp. OR "LMNB1-related".mp. OR "LMNB1".mp. OR "adult-onset autosomal dominant leukodystrophy with autonomic symptoms".mp. OR "autosomal dominant adult-onset demyelinating leukodystrophy".mp. OR "autosomal dominant leukodystrophy with autonomic symptoms".mp. OR "autosomal dominant Pelizaeus-Merzbacher disease".mp. OR "lamin B1-related".mp. OR "lamin-B1-related".mp. OR “Cockayne syndrome”.mp. OR ERCC6.mp. OR ERCC8.mp. OR ERCC2.mp. OR ERCC3.mp. OR "Trichothiodystrophy".mp. OR "ERCC2".mp. OR "ERCC3".mp. OR "GTF2H5".mp. OR "MPLKIP".mp. OR "RNF113A".mp. OR "Salla disease".mp. OR "Salla’s disease".mp. OR "free sialic acid storage disorder*".mp. OR "free sialic acid storage disease*".mp. OR "intermediate-severe Salla disease".mp. OR "infantile free sialic storage disease".mp. OR "SLC17A5".mp. OR "Fucosidosis".mp. OR "FUCA1".mp. OR "alpha-L fucosidase".mp. OR "fucosidase".mp. OR "Adult polyglucosan body disease".mp. OR "APBD".mp. OR "GBE1".mp. OR "glycogen storage disease IV".mp. OR "Methylenetetrahydrofolate reductase deficiency".mp. OR "MTHFR".mp. OR "Small vessel disease with ocular abnormalities".mp. OR "Gould syndrome".mp. OR "COL4A1".mp. OR "hereditary angiopathy with nephropathy, aneurysms and muscle cramps".mp. OR "HANAC".mp. OR "porencephaly type 1".mp. OR "porencephaly type I".mp. OR "brain small-vessel disease with or without ocular anomalies".mp. OR "retinal vasculopathy with cerebral leukodystrophy".mp. OR "RVCL".mp. OR "COL4A2".mp. OR "Gould syndrome 2".mp. OR "porencephaly type 2".mp. OR "porencephaly 2".mp. OR "poren2".mp. OR "Progressive early childhood-onset leukodystrophy".mp. OR "ACER3".mp. OR "PLDECO".mp. OR "leukodystrophy due to alkaline ceramidase 3 deficiency".mp. OR "ACER3-related".mp. OR "Cerebroretinal microangiopathy with calcifications and cysts".mp. OR "CTC1".mp. OR "CMRCC".mp. OR "coats plus".mp. OR "coats disease".mp. OR "Sjogren-Larsson syndrome".mp. OR "SLS".mp. OR "ALDH3A2".mp. OR "RNASET2-deficient leukoencephalopathy".mp. OR	

	"RNASET2".mp. OR "RNAse T2 deficient leukoencephalopathy".mp. OR "Mucopolysaccharidoses".mp. OR "MPS".mp. OR "Hurler syndrome".mp. OR "Hurler-Scheie syndrome".mp. OR "Scheie syndrome".mp. OR "Hunter syndrome".mp. OR "Sanfilippo syndrome".mp. OR "Morquio syndrome".mp. OR "Maroteaux-Lamy syndrome".mp. OR "Sly syndrome".mp. OR "Natowicz syndrome".mp. OR "ARSB".mp. OR "GALNS".mp. OR "GLB1".mp. OR "GNPTAB".mp. OR "GNPTG".mp. OR "GNS".mp. OR "GUSB".mp. OR "HGSNAT".mp. OR "IDS".mp. OR "IDUA".mp. OR "NAGLU".mp. OR "SGSH".mp. OR "Autosomal recessive spastic ataxia with leukoencephalopathy".mp. OR "ARSAL".mp. OR "MARS2".mp. OR "methionyl-tRNA synthetase 2".mp. OR "mt-metRS".mp. OR "COA8".mp. OR "APOPT1".mp. OR "COA8-related".mp. OR "Mitochondrial complex IV deficiency nuclear type 17".mp. OR "MC4DN17".mp. OR "2-hydroxyglutaric aciduria".mp. OR "combined D-2- and L-2-hydroxyglutaric aciduria".mp. OR "D2L2AD".mp. OR "2HGA".mp. OR "D2HGDH".mp. OR "IHD2".mp. OR "L2HGDH".mp. OR "SLC25A1".mp. OR "NKX6-2-related spastic ataxia with hypomyelination".mp. OR "SPAX8".mp. OR "NKX6-2".mp. OR "spastic ataxia 8, autosomal recessive with hypomyelinating leukodystrophy".mp. OR "CNP-related hypomyelinating leukodystrophy".mp. OR "CNP".mp. OR "hypomyelinating leukodystrophy 20".mp. OR "HLD20".mp. OR "CNTNAP1-related arthrogryposis and leukodystrophy".mp. OR "CNTNAP1".mp. OR "hypomyelinating neuropathy, congenital 3".mp. OR "congenital hypomyelinating neuropathy-3".mp. OR "congenital hypomyelinating neuropathy 3".mp. OR "CHN3".mp. OR "MAG-related PMLD".mp. OR "MAG-related Pelizaeus-Merzbacher-like disease".mp. OR "MAG".mp. OR "spastic 75".mp. OR "SPG75".mp. OR “MAL-related leukodystrophy”.mp. OR MAL.mp. OR "SLC35B-related chondrodysplasia with hypomyelinating leukodystrophy".mp. OR "SLC35B2".mp. OR "PAPST1".mp. OR "HLD26".mp. OR "hypomyelinating leukodystrophy with chondrodysplasia".mp. OR "TMEM163-related ".mp. OR "HLD25".mp. OR "TMEM163".mp. OR "DEGS1-related".mp. OR "DEGS1-HLD".mp. OR "HLD18".mp. OR "DEGS1".mp. OR "TMEM63A-related".mp. OR "TMEM63A-HLD".mp. OR "HLD19".mp. OR "TMEM63A".mp. OR "HSPD1-related leukodystrophy".mp. OR HSPD1.mp. OR "RARS1-related".mp. OR "RARS-related".mp. OR "RARS1".mp. OR "RARS".mp. OR "HLD9".mp. OR "arginyl-tRNA synthetase".mp. OR "argRS".mp. OR "PYCR2-related".mp. OR "HLD10".mp. OR "VPS11-related".mp. OR "HLD12".mp. OR "VPS11".mp. OR "HLD13".mp. OR "C11ORF73".mp. OR "HIKESHI".mp. OR "C11ORF3-related".mp. OR "EPRS1-related".mp. OR “EPRS-related”.mp. OR "EPRS1-HLD".mp. OR "EPRS-HLD".mp. OR "EPRS1".mp. OR "EPRS".mp. OR "HLD15".mp. OR "Glutamyl-prolyl-tRNA synthetase".mp. OR "GluProRS".mp. OR "CLDN11-related".mp. OR "HLD22".mp. OR "CLDN11".mp. OR "RNF220-related".mp. OR "HLD23".mp. OR "RNF220".mp. OR "ATP11A-	

	related".mp. OR "HLD24".mp. OR "ATP11A".mp. OR "AARS1-related".mp. OR "AARS-related".mp. OR "AARS".mp. OR "AARS1".mp. OR "developmental and epileptic encephalopathy 29".mp. OR "EIEE29".mp. OR "hereditary diffuse leukoencephalopathy with spheroids 2".mp. OR "HDLS2".mp. OR "alanyl-tRNA synthetase".mp. OR "AlaRS".mp. OR "AARS2-related".mp. OR "combined oxidative phosphorylation deficiency 8".mp. OR "COXPD8".mp. OR "progressive leukoencephalopathy with ovarian failure".mp. OR "ovarioleukodystrophy".mp. OR "AARS2".mp. OR "alanyl-tRNA synthetase 2".mp. OR "mt-AlaRS".mp. OR "Leukoencephalopathy with thalamus and brainstem involvement and high lactate".mp. OR "LTBL".mp. OR "EARS2".mp. OR "EARS2-related".mp. OR "combined oxidative phosphorylation deficiency 12".mp. OR "COXPD12".mp. OR "glutamyl-tRNA synthetase 2".mp. OR "mt-GluRS".mp. OR "Hypomyelination with brainstem and spinal cord involvement and leg spasticity".mp. OR "HBSL".mp. OR "DARS1".mp. OR "DARS".mp. OR "DARS1-related".mp. OR "DARS-related".mp. OR "aspartyl-tRNA synthetase".mp. OR "Asp-RS".mp. OR "WARS2-related leukoencephalopathy".mp. OR "WARS2-related leukodystrophy".mp. OR "WARS2".mp. OR "tryptophanyl-tRNA synthetase".mp. OR "mt-TryRS".mp. OR "childhood-onset parkinsonism-dystonia 3".mp. OR "PKDYS3".mp. OR "KARS-related".mp. OR "KARS1-related".mp. OR "infantile-onset progressive leukoencephalopathy with or without deafness".mp. OR "LEPID".mp. OR "KARS1".mp. OR "lysyl-tRNA synthetase".mp. OR "LysRS".mp. OR "KARS2".mp. OR "KARS2-related".mp. OR "congenital deafness and adult-onset progressive leukoencephalopathy".mp. OR "LSM7-related leukodystrophy".mp. OR "LSM7".mp. OR "POLR1A-related leukodystrophy".mp. OR "HLD27".mp. OR "POLR1A".mp. OR "Neurodevelopmental disorder with spasticity, hypomyelinating leukodystrophy and brain abnormalities".mp. OR "NEDSPLB".mp. OR "polymicrogyria, perisylvian with cerebellar hypoplasia and arthrogryposis".mp. OR "PMGYCHA".mp. OR "PI4KA".mp. OR "PI4KA-related".mp. OR "PI4KA-spectrum".mp. OR "Leukodystrophy and acquired microcephaly with or without dystonia".mp. OR "LDAMD".mp. OR "PLEKHG2".mp. OR "Retinal dystrophy with leukodystrophy".mp. OR "RDLKD".mp. OR "ACBD5".mp. OR "Remitting childhood-onset leukodystrophy".mp. OR "Leukodystrophy, childhood-onset, remitting".mp. OR "CORLK".mp. OR "FBP2".mp. OR "FBP2-related".mp. OR "CLDN25-related".mp. OR "CLDN25-PMLD".mp. OR "CLDN25".mp. OR "Peroxisomal acyl-CoA oxidase deficiency".mp. OR "straight-chain acyl-CoA oxidase deficiency".mp. OR "pseudoneonatal adrenoleukodystrophy".mp. OR "ACOX1".mp. OR "Developmental delay, dysmorphic facies, and brain anomalies".mp. OR "DEVDFB".mp. OR "U2AF2".mp. OR "U2AF2-related".mp. OR "Leukoencephalopathy with ataxia".mp. OR "CLCN2".mp. OR "CLC2".mp. OR "LKPAT".mp OR "2,4-dienoyl-	

	CoA reductase deficiency".mp. OR "DECRD".mp. OR "NADK2".mp. OR "nad kinase 2".mp. OR "progressive encephalopathy with leukodystrophy due to DECR deficiency".mp. OR "DECR deficiency with hyperlysinemia".mp. OR "3-methylcrotonyl-CoA carboxylase 1 deficiency".mp. OR "MCC1D".mp. OR "MCCD type 1".mp. OR "MCC1 deficiency".mp. OR "3-methylcrotonylglycinuria I".mp. OR "methylcrotonylglycinuria type I".mp. OR "MCCC1".mp. OR "3-methylcrotonyl-CoA carboxylase I".mp. OR "Cerebroretinal microangiopathy with calcifications and cysts 2".mp. OR "CRMCC2".mp. OR "CRMCC2-related".mp. OR "STN1".mp. OR "Neurodevelopment disorder with seizures, hypotonia and brain imaging abnormalities".mp. OR "NEDSHBA".mp. OR "GRM7".mp. OR "GRM7-related".mp. OR "L-2-hydroxyglutaric aciduria".mp. OR "L2HGA".mp. OR "L2HGDH".mp. OR "L-2-hydroxyglutarate dehydrogenase".mp. OR "ReNU syndrome".mp. OR "RENU".mp. OR "neurodevelopmental disorder with hypotonia, brain anomalies, distinctive facies, and absent language".mp. OR "NEDHAFA".mp. OR "RNU4-2".mp. OR "Spondyloenchondrodysplasia with immune dysregulation".mp. OR "SPENCDI".mp. OR "SPENCD".mp. OR "combined immunodeficiency with autoimmunity and spondylometaphyseal dysplasia".mp. OR "ACP5".mp. OR "Tartrate-resistant acid phosphatase".mp. OR "TRAP".mp. OR "Fatty acid 2-hydroxylase deficiency".mp. OR "fatty acid hydroxylase-associated neurodegeneration".mp. OR "FAHN".mp. OR "FA2H".mp. OR "hereditary spastic paraplegia 35".mp. OR "HSP35".mp. OR "SPG35".mp. OR "Fahn leukodystrophy".mp. OR "Phenylketonuria".mp. OR "PKU".mp. OR "phenylalanine hydroxylase deficiency".mp. OR "Cx32-related charcot-marie-tooth disease".mp. OR "GJB1".mp. OR "Leukoencephalopathy with ataxia".mp. OR "LKPAT".mp. OR "Giant axonal neuropathy".mp. OR "GAN".mp. OR "GAN-related neurodegeneration".mp. OR "Infantile neuronal ceroid lipofuscinosis".mp. OR "classic infantile CLN1 disease".mp. OR "CLN1".mp. OR "INCL".mp. OR "Batten".mp. OR "PPT1".mp. OR "AGC1-related disease".mp. OR "AGC1".mp. OR "SLC25A12".mp. OR "developmental and epileptic encephalopathy 39 with leukodystrophy".mp. OR "DEE39".mp. OR "Aspartate-glutamate carrier 1 deficiency".mp. OR "Aralar".mp. OR "18q minus syndrome".mp. OR "18q23".mp. OR "18q deletion syndrome".mp. OR "18q-".mp. OR "18q syndrome with deficiency of myelin basic protein".mp. OR "Acute reversible leukoencephalopathy and alpha-ketoglutarate accumulation".mp. OR "ARLIAK".mp. OR "SLC13A3".mp. OR Homocystinuria.mp. OR CBS.mp. OR "Mitochondrial DNA depletion syndrome 1".mp. OR "MTDPS1".mp. OR "Mitochondrial neurogastrointestinal encephalopathy".mp. OR "MNGIE".mp. OR "TYMP".mp. OR "MNGIE syndrome".mp. OR "thymidine phosphorylase deficiency".mp. OR "Mitochondrial DNA depletion syndrome 6".mp. OR "MTDPS6".mp. OR "MPV17".mp. OR "Mucolipidosis IV".mp. OR "MLIV".mp. OR "MCOLN1".mp. OR "mucolipin 1".mp. OR "FOLR1-related cerebral folate transport	

	deficiency".mp. OR "FOLR1-CFTD".mp. OR "cerebral folate deficiency".mp. OR "FOLR1".mp. OR "Neurodegeneration due to cerebral folate transport deficiency".mp. OR "NCFTD".mp. OR "Multiple mitochondrial dysfunctions syndrome".mp. OR "MMDS1".mp. OR "NFU1".mp. OR "MMDS2".mp. OR "BOLA3".mp. OR "MMDS3".mp. OR "IBA57".mp. OR "MMDS4".mp. OR "ISCA2".mp. OR "MMDS5".mp. OR "ISCA1".mp. OR "Mitochondrial complex I deficiency".mp. OR "MC1DN21".mp. OR "NUBPL".mp. OR "IND1".mp. OR "MC1DN5".mp. OR "NDUFS1".mp. OR "MC1DN1".mp. OR "NDUFS4".mp. OR "MC1DN2".mp. OR "NDUFS8".mp. OR "MC1DN4".mp. OR "NDUFV1".mp. OR "MC1DN27".mp. OR "combined oxidative phosphorylation deficiency 15".mp. OR "COXPD15".mp. OR "MTFMT".mp. OR "MC1DN13".mp. OR "NDUFA2".mp. OR "Mitochondrial complex II deficiency".mp. OR "MC2DN1".mp. OR "neurodegeneration with ataxia and late-onset optic atrophy".mp. OR "NDAXOA".mp. OR "SDHA".mp. OR "MC2DN4".mp. OR "SDHB".mp. OR "MC2DN2".mp. OR "SDHAF1".mp. OR "Mitochondrial complex III deficiency".mp. OR "MC3DN8".mp. OR "LYRM7".mp. OR "Mitochondrial complex IV deficiency".mp. OR "MC4DN3".mp. OR "COX10".mp. OR "COA8-related leukoencephalopathy".mp. OR "COA8".mp. OR "APOPT1".mp. OR "MC4DN17".mp. OR "MC4DN1".mp. OR "SURF1".mp. OR "MC4DN7".mp. OR "Combined oxidative phosphorylation deficiency 5".mp. OR "COXPD5".mp. OR "MRPS22".mp. OR "COXPD4".mp. OR "TUFM".mp. OR "COXPD13".mp. OR "PNPT1".mp. OR "COXPD1".mp. OR "GFM1".mp. OR "Perrault syndrome 3".mp. OR "PRLTS3".mp. OR "deafness, autosomal recessive 81".mp. OR "DFNB81".mp. OR "CLPP".mp.	
TOTAL		980
**Embase**		
Human (1)	exp Humans/ or Human*.mp. or "Human Physiology".mp. or "Homo sapiens".mp. or "Human Cells".mp. or "Human-Derived".mp. OR Patient*.mp.	31738246
iPSCs (2)	exp Induced Pluripotent Stem Cells/ or "Pluripotent Stem Cell*".mp. or "Stem Cell Model*".mp. or iPSC*.mp. or iPS.mp.	94033
Leukodystrophies (3)	leukodystroph*.mp. OR leukoencephalopath*.mp. OR "white matter dis*".mp. OR hypomyelinat*.mp. OR dysmyelinat*.mp. OR demyelinate*.mp. OR "myelin vacuolization".mp. OR "myelin dis*".mp. OR leuko-axonopath*.mp. OR astrocytopath*.mp. OR microgliopath*.mp. OR leuko-vasculopath*.mp. OR exp Adrenoleukodystrophy/ OR Adrenoleukodystrophy.mp. OR ALD.mp. OR adrenomyeloneuropathy.mp. OR AMN.mp. OR ccALD.mp. OR acALD.mp. OR ABCD1.mp. OR X-ALD.mp. OR exp Metachromatic Leukodystrophy/ OR MLD.mp. OR ARSA.mp. OR SapB.mp. OR "Arylsulfatase A".mp. OR "Saposin B".mp. OR "Metachromatic Leukoencephalopathy".mp. OR "Diffuse Cerebral Sclerosis,	665193

	Metachromatic Form".mp. OR "Sulfatide Lipidosis".mp. OR "Arylsulfatase A Deficiency".mp. OR "ARSA Deficiency".mp. OR "Cerebroside Sulfatase Deficiency".mp. OR MLDSAPB.mp. OR "Saposin B Deficiency".mp. OR PSAP.mp. OR Prosaposin.mp. OR Krabbe.mp. OR "Globoid cell leukodystrophy".mp. OR GALC.mp. OR galactocerebrosidase.mp. OR galactosylceramidase.mp. OR "Cerebrotendinous xanthomatosis".mp. OR CTX.mp. OR CYP27A1.mp. OR "Pelizaeus-Merzbacher disease".mp. OR PMD.mp. OR "hypomyelination of early myelinating structures".mp. OR HEMS.mp. OR "PLP1-null syndrome".mp. OR "spastic paraplegia type 2".mp. OR SPG2.mp. OR PLP1.mp. OR HLD1.mp. OR "POLR3-related leukodystrophy".mp. OR POLR3-HLD.mp. OR 4H.mp. OR "RNA polymerase III".mp. OR POLR3A.mp. OR POLR3B.mp. OR POLR1C.mp. OR POLR3D.mp. OR POLR3K.mp. OR HLD7.mp. OR HLD8.mp. OR HLD11.mp. OR HLD21.mp. OR "Tremor Ataxia with Central Hypomyelination".mp. OR TACH.mp. OR ADDH.mp. OR "Ataxia Delayed Dentition and Hypomyelination".mp. OR "4H syndrome".mp. OR HCAHC.mp. OR "hypomyelination with cerebellar atrophy and hypoplasia of the corpus callosum".mp. OR Canavan*.mp. OR aspartoacylase.mp. OR ASPA.mp. OR Alexander*.mp. OR AxD.mp. OR ALXDRD.mp. OR "Vanishing white matter".mp. OR VWM.mp OR "childhood ataxia with central hypomyelination".mp. OR CACH.mp. OR eIF2B.mp. OR EIF2B1.mp. OR EIF2B2.mp OR EIF2B3.mp OR EIF2B4.mp. OR EIF2B5.mp. OR "Cree Leukoencephalopathy".mp. OR "Aicardi-Goutières syndrome".mp. OR AGS.mp. OR TREX1.mp. or RNASEH2A.mp. OR RNASEH2B.mp. OR RNASEH2C.mp. OR ADAR.mp. OR SAMHD1.mp. OR IFIH1.mp. OR LSM11.mp. OR RNU7-1.mp. OR "Cree encephalitis".mp. OR "Cerebral leukodystrophy with retinal vasculopathy".mp. OR "Oculodentodigital dysplasia".mp. OR ODDD.mp. OR "Hypomyelination with atrophy of the basal ganglia and cerebellum".mp. OR "H-ABC".mp. OR "TUBB4A".mp. OR "B-tubulin-4A".mp. OR "TUBB4A-related".mp. OR "HLD6".mp. OR "UMF1".mp. OR "HLD14".mp. OR "Leukoencephalopathy with brain stem and spinal cord involvement and lactate elevation".mp. OR "LBSL".mp. OR "DARS2".mp. OR "aspartyl-tRNA synthetase".mp. OR "mt-AspRS".mp. OR "Nasu-Hakola disease".mp. OR "NHD".mp. OR "polycystic lipomembranous osteodysplasia with sclerosing leukoencephalopathy".mp. OR "PLOSL".mp. OR "Adult-onset leukoencephalopathy with axonal spheroids".mp. OR "ALSP".mp. OR "hereditary diffuse leukoencephalopathy with spheroids".mp. OR "HDLS".mp. OR "pigmentary orthochromatic leukodystrophy".mp. OR "POLD".mp. OR "CSF1R".mp. OR "CSF1R-related".mp. OR "brain abnormalities, neurodegeneration, and dysosteosclerosis".mp. OR "BANDDOS".mp. OR "Cerebral autosomal dominant arteriopathy with subcortical infarcts and leukoencephalopathy".mp. OR "CADASIL".mp. OR "NOTCH3".mp. OR "Leukoencephalopathy with calcifications and cysts".mp. OR "LCC".mp. OR "U8".mp. OR "SNORD118".mp. OR "Labrune syndrome".mp. OR "CARASAL".mp.	

	OR “Cathepsin-A related arteriopathy with strokes and leukoencephalopathy”.mp. OR CTSA.mp. OR "Waardenburg-Hirschsprung".mp. OR "PCWH".mp. OR "Waardenburg syndrome".mp. OR "SOX10-related".mp. OR "Multiple sulfatase deficiency".mp. OR "MSD".mp. OR "SUMF1".mp. OR "PMD-like".mp. OR "Pelizaeus-Merzbacher-like disease".mp. OR "Cx47-related".mp. OR "Cx47".mp. OR "AHDS".mp. OR "Allan-Herndon-Dudley syndrome".mp. OR "SLC16A2".mp. OR "MCT8".mp. OR "Hypomyelination with congenital cataract*".mp. OR "HCC".mp. OR "FAM126A".mp. OR "HYCC1".mp. OR "DRCTNNB1A".mp. OR "HLD5".mp. OR "Hypomyelination with spondylometaphyseal dysplasia".mp. OR "AIFM1".mp. OR "PDCD8".mp. OR "Spondyloepimetaphyseal dysplasia with hypomyelinating leukodystrophy".mp. OR "SEMDHL".mp. OR "Spondyloepimetaphyseal dysplasia, X-linked, with hypomyelinating leukodystrophy".mp. OR "leukoencephalopahy with metaphyseal chondrodysplasia".mp. OR "LKMCD".mp. OR "spondyloepimetaphyseal dysplasia, x-linked, with mental deterioration".mp. OR "Megalencephalic leukoencephalopathy with subcortical cysts".mp. OR MLC.mp. OR MLC1.mp. OR MLC2.mp. OR HEPACAM.mp. OR GLIALCAM.mp. OR AQP4.mp. OR GPRC5B.mp. OR "Gangliosidosis".mp. OR "GM1".mp. OR "beta-galactosidase-1 deficiency".mp. OR "GLB1".mp. OR "generalized gangliosidosis".mp. OR "GLB1 deficiency".mp. OR "GM2".mp. OR "Tay-Sachs".mp. OR "GM2-gangliosidosis type I".mp. OR "B variant GM2-gangliosidosis".mp. OR "hexosaminidase A deficiency".mp. OR "HexA".mp. OR "GM2A".mp. OR "Sandhoff".mp. OR "GM2-gangliosidosis type II".mp. OR "hexosaminidases A and B deficiency".mp. OR "HexB".mp. OR "AIMP1-related".mp. OR "AIMP1".mp. OR "EMAP2".mp. OR "EMAPII".mp. OR "HLD3".mp. OR "AIMP2-related".mp. OR "AIMP2".mp. OR "HLD17".mp. OR "HSPD1-related".mp. OR "HSPD1".mp. OR "HSP60".mp. OR "HLD4".mp. OR "MitCHAP60".mp. OR "CPN60".mp. OR "mitochondrial HSP60 chaperonopathy".mp. OR "Cerebral autosomal recessive arteriopathy with subcortical infarcts and leukoencephalopathy".mp. OR "cerebral AR arteriopathy with subcortical infarcts and leukoencephalopathy".mp. OR "CARASIL".mp. OR "HTRA1-related cerebral small vessel disease".mp. OR "HTRA1".mp. OR "Adult-onset autosomal dominant leukodystrophy".mp. OR "ADLD".mp. OR "LMNB1-related".mp. OR "LMNB1".mp. OR "adult-onset autosomal dominant leukodystrophy with autonomic symptoms".mp. OR "autosomal dominant adult-onset demyelinating leukodystrophy".mp. OR "autosomal dominant leukodystrophy with autonomic symptoms".mp. OR "autosomal dominant Pelizaeus-Merzbacher disease".mp. OR "lamin B1-related".mp. OR "lamin-B1-related".mp. OR “Cockayne syndrome”.mp. OR ERCC6.mp. OR ERCC8.mp. OR ERCC2.mp. OR ERCC3.mp. OR "Trichothiodystrophy".mp. OR "ERCC2".mp. OR "ERCC3".mp. OR "GTF2H5".mp. OR "MPLKIP".mp. OR	

	"RNF113A".mp. OR "Salla disease".mp. OR "Salla's disease".mp. OR "free sialic acid storage disorder*".mp. OR "free sialic acid storage disease*".mp. OR "intermediate-severe Salla disease".mp. OR "infantile free sialic storage disease".mp. OR "SLC17A5".mp. OR "Fucosidosis".mp. OR "FUCA1".mp. OR "alpha-L fucosidase".mp. OR "fucosidase".mp. OR "Adult polyglucosan body disease".mp. OR "APBD".mp. OR "GBE1".mp. OR "glycogen storage disease IV".mp. OR "Methylenetetrahydrofolate reductase deficiency".mp. OR "MTHFR".mp. OR "Small vessel disease with ocular abnormalities".mp. OR "Gould syndrome".mp. OR "COL4A1".mp. OR "hereditary angiopathy with nephropathy, aneurysms and muscle cramps".mp. OR "HANAC".mp. OR "porencephaly type 1".mp. OR "porencephaly type I".mp. OR "brain small-vessel disease with or without ocular anomalies".mp. OR "retinal vasculopathy with cerebral leukodystrophy".mp. OR "RVCL".mp. OR "COL4A2".mp. OR "Gould syndrome 2".mp. OR "porencephaly type 2".mp. OR "porencephaly 2".mp. OR "poren2".mp. OR "Progressive early childhood-onset leukodystrophy".mp. OR "ACER3".mp. OR "PLDECO".mp. OR "leukodystrophy due to alkaline ceramidase 3 deficiency".mp. OR "ACER3-related".mp. OR "Cerebroretinal microangiopathy with calcifications and cysts".mp. OR "CTC1".mp. OR "CMRCC".mp. OR "coats plus".mp. OR "coats disease".mp. OR "Sjogren-Larsson syndrome".mp. OR "SLS".mp. OR "ALDH3A2".mp. OR "RNASET2-deficient leukoencephalopathy".mp. OR "RNASET2".mp. OR "RNAse T2 deficient leukoencephalopathy".mp. OR "Mucopolysaccharidoses".mp. OR "MPS".mp. OR "Hurler syndrome".mp. OR "Hurler-Scheie syndrome".mp. OR "Scheie syndrome".mp. OR "Hunter syndrome".mp. OR "Sanfilippo syndrome".mp. OR "Morquio syndrome".mp. OR "Maroteaux-Lamy syndrome".mp. OR "Sly syndrome".mp. OR "Natowicz syndrome".mp. OR "ARSB".mp. OR "GALNS".mp. OR "GLB1".mp. OR "GNPTAB".mp. OR "GNPTG".mp. OR "GNS".mp. OR "GUSB".mp. OR "HGSNAT".mp. OR "IDS".mp. OR "IDUA".mp. OR "NAGLU".mp. OR "SGSH".mp. OR "Autosomal recessive spastic ataxia with leukoencephalopathy".mp. OR "ARSAL".mp. OR "MARS2".mp. OR "methionyl-tRNA synthetase 2".mp. OR "mt-metRS".mp. OR "COA8".mp. OR "APOPT1".mp. OR "COA8-related".mp. OR "Mitochondrial complex IV deficiency nuclear type 17".mp. OR "MC4DN17".mp. OR "2-hydroxyglutaric aciduria".mp. OR "combined D-2- and L-2-hydroxyglutaric aciduria".mp. OR "D2L2AD".mp. OR "2HGA".mp. OR "D2HGDH".mp. OR "IHD2".mp. OR "L2HGDH".mp. OR "SLC25A1".mp. OR "NKX6-2-related spastic ataxia with hypomyelination".mp. OR "SPAX8".mp. OR "NKX6-2".mp. OR "spastic ataxia 8, autosomal recessive with hypomyelinating leukodystrophy".mp. OR "CNP-related hypomyelinating leukodystrophy".mp. OR "CNP".mp. OR "hypomyelinating leukodystrophy 20".mp. OR "HLD20".mp. OR "CNTNAP1-related arthrogryposis and leukodystrophy".mp. OR "CNTNAP1".mp. OR "hypomyelinating neuropathy, congenital 3".mp. OR "congenital	

	hypomyelinating neuropathy-3".mp. OR "congenital hypomyelinating neuropathy 3".mp. OR "CHN3".mp. OR "MAG-related PMLD".mp. OR "MAG-related Pelizaeus-Merzbacher-like disease".mp. OR "MAG".mp. OR "spastic 75".mp. OR "SPG75".mp. OR “MAL-related leukodystrophy”.mp. OR MAL.mp. OR "SLC35B-related chondrodysplasia with hypomyelinating leukodystrophy".mp. OR "SLC35B2".mp. OR "PAPST1".mp. OR "HLD26".mp. OR "hypomyelinating leukodystrophy with chondrodysplasia".mp. OR "TMEM163-related ".mp. OR "HLD25".mp. OR "TMEM163".mp. OR "DEGS1-related".mp. OR "DEGS1-HLD".mp. OR "HLD18".mp. OR "DEGS1".mp. OR "TMEM63A-related".mp. OR "TMEM63A-HLD".mp. OR "HLD19".mp. OR "TMEM63A".mp. OR "HSPD1-related leukodystrophy".mp. OR HSPD1.mp. OR "RARS1-related".mp. OR "RARS-related".mp. OR "RARS1".mp. OR "RARS".mp. OR "HLD9".mp. OR "arginyl-tRNA synthetase".mp. OR "argRS".mp. OR "PYCR2-related".mp. OR "HLD10".mp. OR "VPS11-related".mp. OR "HLD12".mp. OR "VPS11".mp. OR "HLD13".mp. OR "C11ORF73".mp. OR "HIKESHI".mp. OR "C11ORF3-related".mp. OR "EPRS1-related".mp. OR “EPRS-related”.mp. OR "EPRS1-HLD".mp. OR "EPRS-HLD".mp. OR "EPRS1".mp. OR "EPRS".mp. OR "HLD15".mp. OR "Glutamyl-prolyl-tRNA synthetase".mp. OR "GluProRS".mp. OR "CLDN11-related".mp. OR "HLD22".mp. OR "CLDN11".mp. OR "RNF220-related".mp. OR "HLD23".mp. OR "RNF220".mp. OR "ATP11A-related".mp. OR "HLD24".mp. OR "ATP11A".mp. OR "AARS1-related".mp. OR "AARS-related".mp. OR "AARS".mp. OR "AARS1".mp. OR "developmental and epileptic encephalopathy 29".mp. OR "EIEE29".mp. OR "hereditary diffuse leukoencephalopathy with spheroids 2".mp. OR "HDLS2".mp. OR "alanyl-tRNA synthetase".mp. OR "AlaRS".mp. OR "AARS2-related".mp. OR "combined oxidative phosphorylation deficiency 8".mp. OR "COXPD8".mp. OR "progressive leukoencephalopathy with ovarian failure".mp. OR "ovarioleukodystrophy".mp. OR "AARS2".mp. OR "alanyl-tRNA synthetase 2".mp. OR "mt-AlaRS".mp. OR "Leukoencephalopathy with thalamus and brainstem involvement and high lactate".mp. OR "LTBL".mp. OR "EARS2".mp. OR "EARS2-related".mp. OR "combined oxidative phosphorylation deficiency 12".mp. OR "COXPD12".mp. OR "glutamyl-tRNA synthetase 2".mp. OR "mt-GluRS".mp. OR "Hypomyelination with brainstem and spinal cord involvement and leg spasticity".mp. OR "HBSL".mp. OR "DARS1".mp. OR "DARS".mp. OR "DARS1-related".mp. OR "DARS-related".mp. OR "aspartyl-tRNA synthetase".mp. OR "Asp-RS".mp. OR "WARS2-related leukoencephalopathy".mp. OR "WARS2-related leukodystrophy".mp. OR "WARS2".mp. OR "tryptophanyl-tRNA synthetase".mp. OR "mt-TryRS".mp. OR "childhood-onset parkinsonism-dystonia 3".mp. OR "PKDYS3".mp. OR "KARS-related".mp. OR "KARS1-related".mp. OR "infantile-onset progressive leukoencephalopathy with or without deafness".mp. OR	

	"LEPID".mp. OR "KARS1".mp. OR "lysyl-tRNA synthetase".mp. OR "LysRS".mp. OR "KARS2".mp. OR "KARS2-related".mp. OR "congenital deafness and adult-onset progressive leukoencephalopathy".mp. OR "LSM7-related leukodystrophy".mp. OR "LSM7".mp. OR "POLR1A-related leukodystrophy".mp. OR "HLD27".mp. OR "POLR1A".mp. OR "Neurodevelopmental disorder with spasticity, hypomyelinating leukodystrophy and brain abnormalities".mp. OR "NEDSPLB".mp. OR "polymicrogyria, perisylvian with cerebellar hypoplasia and arthrogryposis".mp. OR "PMGYCHA".mp. OR "PI4KA".mp. OR "PI4KA-related".mp. OR "PI4KA-spectrum".mp. OR "Leukodystrophy and acquired microcephaly with or without dystonia".mp. OR "LDAMD".mp. OR "PLEKHG2".mp. OR "Retinal dystrophy with leukodystrophy".mp. OR "RDLKD".mp. OR "ACBD5".mp. OR "Remitting childhood-onset leukodystrophy".mp. OR "Leukodystrophy, childhood-onset, remitting".mp. OR "CORLK".mp. OR "FBP2".mp. OR "FBP2-related".mp. OR "CLDN25-related".mp. OR "CLDN25-PMLD".mp. OR "CLDN25".mp. OR "Peroxisomal acyl-CoA oxidase deficiency".mp. OR "straight-chain acyl-CoA oxidase deficiency".mp. OR "pseudoneonatal adrenoleukodystrophy".mp. OR "ACOX1".mp. OR "Developmental delay, dysmorphic facies, and brain anomalies".mp. OR "DEVDFB".mp. OR "U2AF2".mp. OR "U2AF2-related".mp. OR "Leukoencephalopathy with ataxia".mp. OR "CLCN2".mp. OR "CLC2".mp. OR "LKPAT".mp OR "2,4-dienoyl-CoA reductase deficiency".mp. OR "DECRD".mp. OR "NADK2".mp. OR "nad kinase 2".mp. OR "progressive encephalopathy with leukodystrophy due to DECR deficiency".mp. OR "DECR deficiency with hyperlysinemia".mp. OR "3-methylcrotonyl-CoA carboxylase 1 deficiency".mp. OR "MCC1D".mp. OR "MCCD type 1".mp. OR "MCC1 deficiency".mp. OR "3-methylcrotonylglycinuria I".mp. OR "methylcrotonylglycinuria type I".mp. OR "MCCC1".mp. OR "3-methylcrotonyl-CoA carboxylase I".mp. OR "Cerebroretinal microangiopathy with calcifications and cysts 2".mp. OR "CRMCC2".mp. OR "CRMCC2-related".mp. OR "STN1".mp. OR "Neurodevelopment disorder with seizures, hypotonia and brain imaging abnormalities".mp. OR "NEDSHBA".mp. OR "GRM7".mp. OR "GRM7-related".mp. OR "L-2-hydroxyglutaric aciduria".mp. OR "L2HGA".mp. OR "L2HGDH".mp. OR "L-2-hydroxyglutarate dehydrogenase".mp. OR "ReNU syndrome".mp. OR "RENU".mp. OR "neurodevelopmental disorder with hypotonia, brain anomalies, distinctive facies, and absent language".mp. OR "NEDHAFA".mp. OR "RNU4-2".mp. OR "Spondyloenchondrodysplasia with immune dysregulation".mp. OR "SPENCDI".mp. OR "SPENCD".mp. OR "combined immunodeficiency with autoimmunity and spondylometaphyseal dysplasia".mp. OR "ACP5".mp. OR "Tartrate-resistant acid phosphatase".mp. OR "TRAP".mp. OR "Fatty acid 2-hydroxylase deficiency".mp. OR "fatty acid hydroxylase-associated neurodegeneration".mp. OR "FAHN".mp. OR "FA2H".mp. OR "hereditary spastic paraplegia 35".mp. OR "HSP35".mp. OR	

	"SPG35".mp. OR "Fahn leukodystrophy".mp. OR "Phenylketonuria".mp. OR "PKU".mp. OR "phenylalanine hydroxylase deficiency".mp. OR "Cx32-related charcot-marie-tooth disease".mp. OR "GJB1".mp. OR "Leukoencephalopathy with ataxia".mp. OR "LKPAT".mp. OR "Giant axonal neuropathy".mp. OR "GAN".mp. OR "GAN-related neurodegeneration".mp. OR "Infantile neuronal ceroid lipofuscinosis".mp. OR "classic infantile CLN1 disease".mp. OR "CLN1".mp. OR "INCL".mp. OR "Batten".mp. OR "PPT1".mp. OR "AGC1-related disease".mp. OR "AGC1".mp. OR "SLC25A12".mp. OR "developmental and epileptic encephalopathy 39 with leukodystrophy".mp. OR "DEE39".mp. OR "Aspartate-glutamate carrier 1 deficiency".mp. OR "Aralar".mp. OR "18q minus syndrome".mp. OR "18q23".mp. OR "18q deletion syndrome".mp. OR "18q-".mp. OR "18q syndrome with deficiency of myelin basic protein".mp. OR "Acute reversible leukoencephalopathy and alpha-ketoglutarate accumulation".mp. OR "ARLIAK".mp. OR "SLC13A3".mp. OR Homocystinuria.mp. OR CBS.mp. OR "Mitochondrial DNA depletion syndrome 1".mp. OR "MTDPS1".mp. OR "Mitochondrial neurogastrointestinal encephalopathy".mp. OR "MNGIE".mp. OR "TYMP".mp. OR "MNGIE syndrome".mp. OR "thymidine phosphorylase deficiency".mp. OR "Mitochondrial DNA depletion syndrome 6".mp. OR "MTDPS6".mp. OR "MPV17".mp. OR "Mucolipidosis IV".mp. OR "MLIV".mp. OR "MCOLN1".mp. OR "mucolipin 1".mp. OR "FOLR1-related cerebral folate transport deficiency".mp. OR "FOLR1-CFTD".mp. OR "cerebral folate deficiency".mp. OR "FOLR1".mp. OR "Neurodegeneration due to cerebral folate transport deficiency".mp. OR "NCFTD".mp. OR "Multiple mitochondrial dysfunctions syndrome".mp. OR "MMDS1".mp. OR "NFU1".mp. OR "MMDS2".mp. OR "BOLA3".mp. OR "MMDS3".mp. OR "IBA57".mp. OR "MMDS4".mp. OR "ISCA2".mp. OR "MMDS5".mp. OR "ISCA1".mp. OR "Mitochondrial complex I deficiency".mp. OR "MC1DN21".mp. OR "NUBPL".mp. OR "IND1".mp. OR "MC1DN5".mp. OR "NDUFS1".mp. OR "MC1DN1".mp. OR "NDUFS4".mp. OR "MC1DN2".mp. OR "NDUFS8".mp. OR "MC1DN4".mp. OR "NDUFV1".mp. OR "MC1DN27".mp. OR "combined oxidative phosphorylation deficiency 15".mp. OR "COXPD15".mp. OR "MTFMT".mp. OR "MC1DN13".mp. OR "NDUFA2".mp. OR "Mitochondrial complex II deficiency".mp. OR "MC2DN1".mp. OR "neurodegeneration with ataxia and late-onset optic atrophy".mp. OR "NDAXOA".mp. OR "SDHA".mp. OR "MC2DN4".mp. OR "SDHB".mp. OR "MC2DN2".mp. OR "SDHAF1".mp. OR "Mitochondrial complex III deficiency".mp. OR "MC3DN8".mp. OR "LYRM7".mp. OR "Mitochondrial complex IV deficiency".mp. OR "MC4DN3".mp. OR "COX10".mp. OR "COA8-related leukoencephalopathy".mp. OR "COA8".mp. OR "APOPT1".mp. OR "MC4DN17".mp. OR "MC4DN1".mp. OR "SURF1".mp. OR "MC4DN7".mp. OR "Combined oxidative phosphorylation deficiency 5".mp. OR "COXPD5".mp. OR "MRPS22".mp. OR "COXPD4".mp. OR "TUFM".mp. OR	

	"COXPD13".mp. OR "PNPT1".mp. OR "COXPD1".mp. OR "GFM1".mp. OR "Perrault syndrome 3".mp. OR "PRLTS3".mp. OR "deafness, autosomal recessive 81".mp. OR "DFNB81".mp. OR "CLPP".mp.	
TOTAL		2019
**Scopus**		
Human (1)	("human*" OR "human" OR "physiology" OR "homo sapiens" OR "human cells" OR "human-derived" OR "patient*")	31169485
iPSCs (2)	("Induced Pluripotent Stem Cells" OR "Pluripotent Stem Cell*" OR "Stem Cell Model*" OR "iPSC*" OR "iPS")	92537
Leukodystrophies (3)	("leukodystroph*" OR "leukoencephalopath*" OR "white matter dis*" OR "hypomyelinat*" OR "dysmyelinat*" OR "demyelinate*" OR "myelin vacuolization" OR "myelin dis*" OR "leuko-axonopath*" OR "astrocytopath*" OR "microgliopath*" OR "leuko-vasculopath*" OR "Adrenoleukodystrophy" OR "ALD" OR "adrenomyeloneuropathy" OR "AMN" OR "ccALD" OR "acALD" OR "ABCD1" OR "X-ALD" OR "Metachromatic Leukodystrophy" OR "MLD" OR "ARSA" OR "SapB" OR "Arylsulfatase A" OR "Saposin B" OR "Metachromatic Leukoencephalopathy" OR "Diffuse Cerebral Sclerosis, Metachromatic Form" OR "Sulfatide Lipidosis" OR "Arylsulfatase A Deficiency" OR "ARSA Deficiency" OR "Cerebroside Sulfatase Deficiency" OR "MLDSAPB" OR "Saposin B Deficiency" OR "PSAP" OR "Prosaposin" OR "Krabbe" OR "Globoid cell leukodystrophy" OR "GALC" OR "galactocerebrosidase" OR "galactosylceramidase" OR "Cerebrotendinous xanthomatosis" OR "CTX" OR "CYP27A1" OR "Pelizaeus-Merzbacher disease" OR "PMD" OR "hypomyelination of early myelinating structures" OR "HEMS" OR "PLP1-null syndrome" OR "spastic paraplegia type 2" OR "SPG2" OR "PLP1" OR "HLD1" OR "POLR3-related leukodystrophy" OR "POLR3-HLD" OR "4H" OR "RNA polymerase III" OR "POLR3A" OR "POLR3B" OR "POLR1C" OR "POLR3D" OR "POLR3K" OR "HLD7" OR "HLD8" OR "HLD11" OR "HLD21" OR "Tremor Ataxia with Central Hypomyelination" OR "TACH" OR "ADDH" OR "Ataxia Delayed Dentition and Hypomyelination" OR "4H syndrome" OR "HCAHC" OR "hypomyelination with cerebellar atrophy and hypoplasia of the corpus callosum" OR "Canavan*" OR "aspartoacylase" OR "ASPA" OR "Alexander*" OR "AxD" OR "ALXDRD" OR "Vanishing white matter" OR "VWM" OR "childhood ataxia with central hypomyelination" OR "CACH" OR "eIF2B" OR "EIF2B1" OR "EIF2B2" OR "EIF2B3" OR "EIF2B4" OR "EIF2B5" OR "Cree Leukoencephalopathy" OR "Aicardi-Goutières syndrome" OR "AGS" OR "TREX1" OR "RNASEH2A" OR "RNASEH2B" OR "RNASEH2C" OR "ADAR" OR "SAMHD1" OR "IFIH1" OR "LSM11" OR "RNU7-1" OR "Cree encephalitis" OR "Cerebral leukodystrophy with retinal vasculopathy" OR "Oculodentodigital dysplasia" OR "ODDD" OR "Hypomyelination with atrophy of the basal ganglia and cerebellum" OR "H-ABC" OR "TUBB4A" OR "B-tubulin-4A" OR "TUBB4A-related"	1308922

	OR "HLD6" OR "UMF1" OR "HLD14" OR "Leukoencephalopathy with brain stem and spinal cord involvement and lactate elevation" OR "LBSL" OR "DARS2" OR "aspartyl-tRNA synthetase" OR "mt-AspRS" OR "Nasu-Hakola disease" OR "NHD" OR "polycystic lipomembranous osteodysplasia with sclerosing leukoencephalopathy" OR "PLOSL" OR "Adult-onset leukoencephalopathy with axonal spheroids" OR "ALSP" OR "hereditary diffuse leukoencephalopathy with spheroids" OR "HDLS" OR "pigmentary orthochromatic leukodystrophy" OR "POLD" OR "CSF1R" OR "CSF1R-related" OR "brain abnormalities, neurodegeneration, and dysosteosclerosis" OR "BANDDOS" OR "Cerebral autosomal dominant arteriopathy with subcortical infarcts and leukoencephalopathy" OR "CADASIL" OR "NOTCH3" OR "Leukoencephalopathy with calcifications and cysts" OR "LCC" OR "U8" OR "SNORD118" OR "Labrune syndrome" OR "CARASAL" OR "Cathepsin-A related arteriopathy with strokes and leukoencephalopathy" OR "CTSA" OR "Waardenburg-Hirschsprung" OR "PCWH" OR "Waardenburg syndrome" OR "SOX10-related" OR "Multiple sulfatase deficiency" OR "MSD" OR "SUMF1" OR "PMD-like" OR "Pelizaeus-Merzbacher-like disease" OR "Cx47-related" OR "Cx47" OR "AHDS" OR "Allan-Herndon-Dudley syndrome" OR "SLC16A2" OR "MCT8" OR "Hypomyelination with congenital cataract*" OR "HCC" OR "FAM126A" OR "HYCC1" OR "DRCTNNB1A" OR "HLD5" OR "Hypomyelination with spondylometaphyseal dysplasia" OR "AIFM1" OR "PDCD8" OR "Spondyloepimetaphyseal dysplasia with hypomyelinating leukodystrophy" OR "SEMDHL" OR "Spondyloepimetaphyseal dysplasia, X-linked, with hypomyelinating leukodystrophy" OR "leukoencephalopathy with metaphyseal chondrodysplasia" OR "LKMCD" OR "spondyloepimetaphyseal dysplasia, x-linked, with mental deterioration" OR "Megalencephalic leukoencephalopathy with subcortical cysts" OR "MLC" OR "MLC1" OR "MLC2" OR "HEPACAM" OR "GLIALCAM" OR "AQP4" OR "GPRC5B" OR "Gangliosidosis" OR "GM1" OR "beta-galactosidase-1 deficiency" OR "GLB1" OR "generalized gangliosidosis" OR "GLB1 deficiency" OR "GM2" OR "Tay-Sachs" OR "GM2-gangliosidosis type I" OR "B variant GM2-gangliosidosis" OR "hexosaminidase A deficiency" OR "HexA" OR "GM2A" OR "Sandhoff" OR "GM2-gangliosidosis type II" OR "hexosaminidases A and B deficiency" OR "HexB" OR "AIMP1-related" OR "AIMP1" OR "EMAP2" OR "EMAPII" OR "HLD3" OR "AIMP2-related" OR "AIMP2" OR "HLD17" OR "HSPD1-related" OR "HSPD1" OR "HSP60" OR "HLD4" OR "MitCHAP60" OR "CPN60" OR "mitochondrial HSP60 chaperonopathy" OR "Cerebral autosomal recessive arteriopathy with subcortical infarcts and leukoencephalopathy" OR "cerebral AR arteriopathy with subcortical infarcts and leukoencephalopathy" OR "CARASIL" OR "HTRA1-related cerebral small vessel disease" OR "HTRA1" OR "Adult-onset autosomal dominant leukodystrophy" OR "ADLD" OR "LMNB1-related" OR "LMNB1" OR "adult-onset autosomal dominant leukodystrophy with autonomic symptoms" OR "autosomal	

	dominant adult-onset demyelinating leukodystrophy" OR "autosomal dominant leukodystrophy with autonomic symptoms" OR "autosomal dominant Pelizaeus-Merzbacher disease" OR "lamin B1-related" OR "lamin-B1-related" OR "Cockayne syndrome" OR "ERCC6" OR "ERCC8" OR "ERCC2" OR "ERCC3" OR "Trichothiodystrophy" OR "ERCC2" OR "ERCC3" OR "GTF2H5" OR "MPLKIP" OR "RNF113A" OR "Salla disease" OR "Salla's disease" OR "free sialic acid storage disorder*" OR "free sialic acid storage disease*" OR "intermediate-severe Salla disease" OR "infantile free sialic storage disease" OR "SLC17A5" OR "Fucosidosis" OR "FUCA1" OR "alpha-L fucosidase" OR "fucosidase" OR "Adult polyglucosan body disease" OR "APBD" OR "GBE1" OR "glycogen storage disease IV" OR "Methylenetetrahydrofolate reductase deficiency" OR "MTHFR" OR "Small vessel disease with ocular abnormalities" OR "Gould syndrome" OR "COL4A1" OR "hereditary angiopathy with nephropathy, aneurysms and muscle cramps" OR "HANAC" OR "porencephaly type 1" OR "porencephaly type I" OR "brain small-vessel disease with or without ocular anomalies" OR "retinal vasculopathy with cerebral leukodystrophy" OR "RVCL" OR "COL4A2" OR "Gould syndrome 2" OR "porencephaly type 2" OR "porencephaly 2" OR "poren2" OR "Progressive early childhood-onset leukodystrophy" OR "ACER3" OR "PLDECO" OR "leukodystrophy due to alkaline ceramidase 3 deficiency" OR "ACER3-related" OR "Cerebroretinal microangiopathy with calcifications and cysts" OR "CTC1" OR "CMRCC" OR "coats plus" OR "coats disease" OR "Sjogren-Larsson syndrome" OR "SLS" OR "ALDH3A2" OR "RNASET2-deficient leukoencephalopathy" OR "RNASET2" OR "RNAse T2 deficient leukoencephalopathy" OR "Mucopolysaccharidoses" OR "MPS" OR "Hurler syndrome" OR "Hurler-Scheie syndrome" OR "Scheie syndrome" OR "Hunter syndrome" OR "Sanfilippo syndrome" OR "Morquio syndrome" OR "Maroteaux-Lamy syndrome" OR "Sly syndrome" OR "Natowicz syndrome" OR "ARSB" OR "GALNS" OR "GLB1" OR "GNPTAB" OR "GNPTG" OR "GNS" OR "GUSB" OR "HGSNAT" OR "IDS" OR "IDUA" OR "NAGLU" OR "SGSH" OR "Autosomal recessive spastic ataxia with leukoencephalopathy" OR "ARSAL" OR "MARS2" OR "methionyl-tRNA synthetase 2" OR "mt-metRS" OR "COA8" OR "APOPT1" OR "COA8-related" OR "Mitochondrial complex IV deficiency nuclear type 17" OR "MC4DN17" OR "2-hydroxyglutaric aciduria" OR "combined D-2- and L-2-hydroxyglutaric aciduria" OR "D2L2AD" OR "2HGA" OR "D2HGDH" OR "IHD2" OR "L2HGDH" OR "SLC25A1" OR "NKX6-2-related spastic ataxia with hypomyelination" OR "SPAX8" OR "NKX6-2" OR "spastic ataxia 8, autosomal recessive with hypomyelinating leukodystrophy" OR "CNP-related hypomyelinating leukodystrophy" OR "CNP" OR "hypomyelinating leukodystrophy 20" OR "HLD20" OR "CNTNAP1-related arthrogryposis and leukodystrophy" OR "CNTNAP1" OR "hypomyelinating neuropathy, congenital 3" OR "congenital hypomyelinating neuropathy-3" OR "congenital hypomyelinating neuropathy 3" OR "CHN3" OR "MAG-	

	related PMLD" OR "MAG-related Pelizaeus-Merzbacher-like disease" OR "MAG" OR "spastic 75" OR "SPG75" OR "MAL-related leukodystrophy" OR "MAL" OR "SLC35B-related chondrodysplasia with hypomyelinating leukodystrophy" OR "SLC35B2" OR "PAPST1" OR "HLD26" OR "hypomyelinating leukodystrophy with chondrodysplasia" OR "TMEM163-related" OR "HLD25" OR "TMEM163" OR "DEGS1-related" OR "DEGS1-HLD" OR "HLD18" OR "DEGS1" OR "TMEM63A-related" OR "TMEM63A-HLD" OR "HLD19" OR "TMEM63A" OR "HSPD1-related leukodystrophy" OR "HSPD1" OR "RARS1-related" OR "RARS-related" OR "RARS1" OR "RARS" OR "HLD9" OR "arginyl-tRNA synthetase" OR "argRS" OR "PYCR2-related" OR "HLD10" OR "VPS11-related" OR "HLD12" OR "VPS11" OR "HLD13" OR "C11ORF73" OR "HIKESHI" OR "C11ORF3-related" OR "EPRS1-related" OR "EPRS-related" OR "EPRS1-HLD" OR "EPRS-HLD" OR "EPRS1" OR "EPRS" OR "HLD15" OR "Glutamyl-prolyl-tRNA synthetase" OR "GluProRS" OR "CLDN11-related" OR "HLD22" OR "CLDN11" OR "RNF220-related" OR "HLD23" OR "RNF220" OR "ATP11A-related" OR "HLD24" OR "ATP11A" OR "AARS1-related" OR "AARS-related" OR "AARS" OR "AARS1" OR "developmental and epileptic encephalopathy 29" OR "EIEE29" OR "hereditary diffuse leukoencephalopathy with spheroids 2" OR "HDLS2" OR "alanyl-tRNA synthetase" OR "AlaRS" OR "AARS2-related" OR "combined oxidative phosphorylation deficiency 8" OR "COXPD8" OR "progressive leukoencephalopathy with ovarian failure" OR "ovarioleukodystrophy" OR "AARS2" OR "alanyl-tRNA synthetase 2" OR "mt-AlaRS" OR "Leukoencephalopathy with thalamus and brainstem involvement and high lactate" OR "LTBL" OR "EARS2" OR "EARS2-related" OR "combined oxidative phosphorylation deficiency 12" OR "COXPD12" OR "glutamyl-tRNA synthetase 2" OR "mt-GluRS" OR "Hypomyelination with brainstem and spinal cord involvement and leg spasticity" OR "HBSL" OR "DARS1" OR "DARS" OR "DARS1-related" OR "DARS-related" OR "aspartyl-tRNA synthetase" OR "Asp-RS" OR "WARS2-related leukoencephalopathy" OR "WARS2-related leukodystrophy" OR "WARS2" OR "tryptophanyl-tRNA synthetase" OR "mt-TryRS" OR "childhood-onset parkinsonism-dystonia 3" OR "PKDYS3" OR “KARS-related" OR "KARS1-related" OR "infantile-onset progressive leukoencephalopathy with or without deafness" OR "LEPID" OR "KARS1" OR "lysyl-tRNA synthetase" OR "LysRS" OR "KARS2" OR "KARS2-related" OR "congenital deafness and adult-onset progressive leukoencephalopathy" OR "LSM7-related leukodystrophy" OR "LSM7" OR "POLR1A-related leukodystrophy" OR "HLD27" OR "POLR1A" OR "Neurodevelopmental disorder with spasticity, hypomyelinating leukodystrophy and brain abnormalities" OR "NEDSPLB" OR "polymicrogyria, perisylvian with cerebellar hypoplasia and arthrogryposis" OR "PMGYCHA" OR "PI4KA" OR "PI4KA-related" OR "PI4KA-spectrum" OR "Leukodystrophy and acquired microcephaly with or without dystonia" OR "LDAMD" OR "PLEKHG2" OR "Retinal dystrophy with leukodystrophy" OR "RDLKD" OR "ACBD5" OR "Remitting childhood-onset leukodystrophy" OR	

	"Leukodystrophy, childhood-onset, remitting" OR "CORLK" OR "FBP2" OR "FBP2-related" OR "CLDN25-related" OR "CLDN25-PMLD" OR "CLDN25" OR "Peroxisomal acyl-CoA oxidase deficiency" OR "straight-chain acyl-CoA oxidase deficiency" OR "pseudoneonatal adrenoleukodystrophy" OR "ACOX1" OR "Developmental delay, dysmorphic facies, and brain anomalies" OR "DEVDFB" OR "U2AF2" OR "U2AF2-related" OR "Leukoencephalopathy with ataxia" OR "CLCN2" OR "CLC2" OR "LKPAT" OR "2,4-dienoyl-CoA reductase deficiency" OR "DECRD" OR "NADK2" OR "nad kinase 2" OR "progressive encephalopathy with leukodystrophy due to DECR deficiency" OR "DECR deficiency with hyperlysinemia" OR "3-methylcrotonyl-CoA carboxylase 1 deficiency" OR "MCC1D" OR "MCCD type 1" OR "MCC1 deficiency" OR "3-methylcrotonylglycinuria I" OR "methylcrotonylglycinuria type I" OR "MCCC1" OR "3-methylcrotonyl-CoA carboxylase I" OR "Cerebroretinal microangiopathy with calcifications and cysts 2" OR "CRMCC2" OR "CRMCC2-related" OR "STN1" OR "Neurodevelopment disorder with seizures, hypotonia and brain imaging abnormalities" OR "NEDSHBA" OR "GRM7" OR "GRM7-related" OR "L-2-hydroxyglutaric aciduria" OR "L2HGA" OR "L2HGDH" OR "L-2-hydroxyglutarate dehydrogenase" OR "ReNU syndrome" OR "RENU" OR "neurodevelopmental disorder with hypotonia, brain anomalies, distinctive facies, and absent language" OR "NEDHAFA" OR "RNU4-2" OR "Spondyloenchondrodysplasia with immune dysregulation" OR "SPENCDI" OR "SPENCD" OR "combined immunodeficiency with autoimmunity and spondylometaphyseal dysplasia" OR "ACP5" OR "Tartrate-resistant acid phosphatase" OR "TRAP" OR "Fatty acid 2-hydroxylase deficiency" OR "fatty acid hydroxylase-associated neurodegeneration" OR "FAHN" OR "FA2H" OR "hereditary spastic paraplegia 35" OR "HSP35" OR "SPG35" OR "Fahn leukodystrophy" OR "Phenylketonuria" OR "PKU" OR "phenylalanine hydroxylase deficiency" OR "Cx32-related charcot-marie-tooth disease" OR "GJB1" OR "Leukoencephalopathy with ataxia" OR "LKPAT" OR "Giant axonal neuropathy" OR "GAN" OR "GAN-related neurodegeneration" OR "Infantile neuronal ceroid lipofuscinosis" OR "classic infantile CLN1 disease" OR "CLN1" OR "INCL" OR "Batten" OR "PPT1" OR "AGC1-related disease" OR "AGC1" OR "SLC25A12" OR "developmental and epileptic encephalopathy 39 with leukodystrophy" OR "DEE39" OR "Aspartate-glutamate carrier 1 deficiency" OR "Aralar" OR "18q minus syndrome" OR "18q23" OR "18q deletion syndrome" OR "18q-" OR "18q syndrome with deficiency of myelin basic protein" OR "Acute reversible leukoencephalopathy and alpha-ketoglutarate accumulation" OR "ARLIAK" OR "SLC13A3" OR "Homocystinuria" OR "CBS" OR "Mitochondrial DNA depletion syndrome 1" OR "MTDPS1" OR "Mitochondrial neurogastrointestinal encephalopathy" OR "MNGIE" OR "TYMP" OR "MNGIE syndrome" OR "thymidine phosphorylase deficiency" OR "Mitochondrial DNA depletion syndrome 6" OR	

	"MTDPS6" OR "MPV17" OR "Mucolipidosis IV" OR "MLIV" OR "MCOLN1" OR "mucolipin 1" OR "FOLR1-related cerebral folate transport deficiency" OR "FOLR1-CFTD" OR "cerebral folate deficiency" OR "FOLR1" OR "Neurodegeneration due to cerebral folate transport deficiency" OR "NCFTD" OR "Multiple mitochondrial dysfunctions syndrome" OR "MMDS1" OR "NFU1" OR "MMDS2" OR "BOLA3" OR "MMDS3" OR "IBA57" OR "MMDS4" OR "ISCA2" OR "MMDS5" OR "ISCA1" OR "Mitochondrial complex I deficiency" OR "MC1DN21" OR "NUBPL" OR "IND1" OR "MC1DN5" OR "NDUFS1" OR "MC1DN1" OR "NDUFS4" OR "MC1DN2" OR "NDUFS8" OR "MC1DN4" OR "NDUFV1" OR "MC1DN27" OR "combined oxidative phosphorylation deficiency 15" OR "COXPD15" OR "MTFMT" OR "MC1DN13" OR "NDUFA2" OR "Mitochondrial complex II deficiency" OR "MC2DN1" OR "neurodegeneration with ataxia and late-onset optic atrophy" OR "NDAXOA" OR "SDHA" OR "MC2DN4" OR "SDHB" OR "MC2DN2" OR "SDHAF1" OR "Mitochondrial complex III deficiency" OR "MC3DN8" OR "LYRM7" OR "Mitochondrial complex IV deficiency" OR "MC4DN3" OR "COX10" OR "COA8-related leukoencephalopathy" OR "COA8" OR "APOPT1" OR "MC4DN17" OR "MC4DN1" OR "SURF1" OR "MC4DN7" OR "Combined oxidative phosphorylation deficiency 5" OR "COXPD5" OR "MRPS22" OR "COXPD4" OR "TUFM" OR "COXPD13" OR "PNPT1" OR "COXPD1" OR "GFM1" OR "Perrault syndrome 3" OR "PRLTS3" OR "deafness, autosomal recessive 81" OR "DFNB81" OR "CLPP")	
Total		1859

Supplementary Table 2. Published iPSC lines that have been generated and characterized for future use in disease modeling.

Disease	Gene(s)	iPSC Lines	Reprogramming Method(s)	References
**Myelin Disorders**				
Demyelinating Disorders				
X-ALD	*ABCD1*	1. Pd iPSC, AMN: *ABCD1* (HEMI), c.1534G>A (p.G512S)<br>2. Pd iPSC, AMN: *ABCD1* (HEMI), c.1968_1970delCAT, p.I657del	Sendai virus (Fibroblasts)	Son et al. 2017
		1. Pd iPSC, ccALD: *ABCD1* (HEMI), c.1661G>A (p.R554H)	Sendai virus (Fibroblasts)	You et al. 2017
		1. Pd iPSC, acALD: *ABCD1* (HEMI), c.1240_1253 delGGCTACACAGCCCGinsCCGTCC	Sendai virus (Fibroblasts)	Yeon et al. 2019
		1. Pd iPSC, UNK: *ABCD1* (HEMI), c.2013insA	Sendai virus (Urine cells)	Wang et al. 2021
		1. Pd iPSC, UNK: *ABCD1* (HEMI), c.1201C>T (p.R401W)<br>2. Pd iPSC, UNK: *ABCD1* (HEMI), c.2010GCTAC>TAT (p.L670Ffs*63)	Retroviral (Fibroblasts)	Kuramochi et al. 2021
		1. Pd iPSC, UNK: *ABCD1* (HEMI), Isogenic corrected line of c.1534G>A	Sendai virus (Fibroblasts)	Sik Jung et al. 2022
		1. Pd iPSC, ccALD: *ABCD1* (HEMI), c.1676A>G (p.Y559C)<br>2. Pd iPSC, adrenocortical insufficiency: *ABCD1* (HEMI), c.906G>T (p.E302D)	Sendai virus (Fibroblasts)	Wang et al. 2023
		1. Pd iPSC, UNK: *ABCD1* (HEMI), c.871G>A (p.E291K)	Sendai virus (PBMCs)	Gornostal et al. 2024
Krabbe	*GALC*	1. Pd iPSC, UNK: *GALC* (cHET), c.461C>A; c.1244G>A	Sendai virus (Fibroblasts)	Wang et al. 2020
CTX	*CYP27A1*	1. Pd iPSC, UNK: *CYP27A1* (HMZ), c.1183C>A (p.R395S)	Episomal vectors (Fibroblasts)	Hoflinger et al. 2016
Hypomyelinating Disorders				
PMD	*PLP1*	1. Pd iPSC, UNK: *PLP1* (HEMI), c.643C>T (p.215S)	Retroviral (Fibroblasts)	Kim et al. 2020
		1. Pd iPSC, UNK: *PLP1* (HEMI), c.437T>C (p.L146P)	Episomal plasmids (PBMCs)	Liu et al. 2020
		1. GE iPSC: *PLP1* (HEMI), c.98G>A, p.C33Y	Episomal vectors (UCBC)	Schreiber et al. 2024
POLR3-HLD	*POLR3A, POLR3B, POLR1C, POLR3D, POLR3K*	1. Pd iPSC, POLR3-HLD: *POLR3A* (cHET), c.1802T>A; c.4072G>A	Episomal vectors (Fibroblasts)	Casamassa et al. 2024
		1. Pd iPSC, SA/HSP: *POLR3A* (cHET), c.1909+22G>A; c.3944_3945delTG<br>2. Pd iPSC, SA/HSP: *POLR3A* (cHET), c.1909+22G>A; c.1531C>T<br>3. Pd iPSC, SA/HSP: *POLR3A* (cHET), c.1909+22G>A; c.2472_2472delC	Episomal vectors (Fibroblasts)	Manibarathi et al. 2024
Cockayne	*ERCC6*	1. Pd iPSC, UNK: *ERCC6* (cHET), c.1131A>T; c.2571C>T	Episomal vectors (Fibroblasts)	Martins et al. 2021
AHDS	*SLC16A2*	1. Pd iPSC, Severe AHDS: *SLC16A2* (HEMI), c.1026+1G>A	Sendai virus (PBMCs)	Wang et al. 2020
		1. GE iPSC: *SLC16A2* (HEMI), p.G401R<br>2. GE iPSC: *SLC16A2* (HEMI), p.F400Sfs*17	RNA (Fibroblasts)	Ludwik et al. 2023
Bloc1s1-LD	*BLOC1S1*	1. Pd iPSC, UNK: *BLOC1S1* (cHET), c.206A>C; c.359G>A^{p}	Sendai virus (PBMCs)	Wu et al. 2022

		2. Pd iPSC, Unaffected carrier: *BLOC1S1* (HET), c.206A>C<br>3. Pd iPSC, Unaffected carrier: *BLOC1S1* (HET), c.359G>A<br>4. Pd iPSC, UNK: *BLOC1S1* (HMZ), c.185T>C		
CLDN11-HLD	*CLDN11*	1. Pd iPSC, UNK: *CLDN11* (HET), c.622T>C (p.*208Glnext*39)	Sendai virus (Fibroblasts)	Ozgoren et al. 2023
FSASD	*SLC17A5*	1. Pd iPSC, Salla disease: *SLC17A5* (HMZ), c.115C>T (p.R39C)<br>2. Pd iPSC, IntSSD: *SLC17A5* (cHET), c.406A>G (p.K136E); c.533delC (p.T178Nfs*34)	Episomal vectors (Fibroblasts)	Sabir et al. 2024
Myelin Vacuolization				
Canavan	*ASPA*	1. Pd iPSC, UNK: *ASPA* (cHET), c.556_559dupGTTC (p.L187Rfs*5); c.919delA (p.S307Vfs*24)	Episomal vectors (PBMCs)	Liu et al. 2024
PKU	*PAH*	1. Pd iPSC, UNK: *PAH* (cHET), c.331C>T; c.975C>G	Sendai virus (PBMCs)	Xu et al. 2017
		1. Pd iPSC, UNK: *PAH* (UNK), UNK	Episomal vectors (Urine cells)	Qi et al. 2018
		1. Pd iPSC, UNK: *PAH* (cHET), c.117C>G (p.F39L); c.IVS10-11G>A (p.?)<br>2. Pd iPSC, UNK: *PAH* (cHET), c.896T>G (p.F299C), c.1222C>T (p.R408W)	Sendai virus (Fibroblasts)	Veleva et al. 2024
		1. Pd iPSC, UNK: *PAH* (cHET), c.590_612del23 (p.?); c.912+1G>A<br>2. Pd iPSC, UNK: *PAH* (cHET), c.755G>A, p.R252Q; c.1315+1G>A	Sendai virus (LCL)	Veleva et al. 2024
Cx32-CMT	*GJB1*	1. Pd iPSC, UNK: *GJB1* (HET), c.139G>A (p.M139V)	Episomal vectors (Fibroblasts)	Son et al. 2017
FAHN	*FA2H*	1. Pd iPSC, UNK: *FA2H* (cHET), p.G45R; p.H319R	Sendai virus (Fibroblasts)	Efendic et al. 2022
		1. Pd iPSC, UNK: *FA2H* (cHET), p.P65S; p.D35Y<br>2. Pd iPSC, UNK: *FA2H* (HMZ), p.Y231H	Sendai virus (Fibroblasts)	Efendic et al. 2023
**Astrocytopathies**				
AGS	*TREX1, RNASEH2B, IFIH1, ADAR1, LSM11, RNU7-1*	1. Pd iPSC, UNK: TREX1 c.260insAG, p.S88fs*22; c.290G>A, p.R97H	Sendai virus (Fibroblasts)	Ferraro et al. 2019
		1. Pd iPSC, UNK: *RNASEH2B* (HMZ), c.529G>A (p.A177T)	Sendai virus (Fibroblasts)	Ferraro et al. 2019
		1. Pd iPSC, UNK: *IF1H1* (HET), c.2471G>A (p.R824K)	Sendai virus (Fibroblasts)	Masneri et al. 2019
		1. Pd iPSC, UNK: *SAMHD1* (HMZ), exon 14-15 deletion	Sendai virus (PBMCs)	Fuchs et al. 2020
		1. Pd iPSC, UNK: *TREX1* (HMZ), c.602T>A (p.V201D)<br>2. Pd iPSC, UNK: *TREX1* (HMZ), c.341G>A (p.R114H)	Sendai virus (Fibroblasts)	Hanchen et al. 2022
		1. Pd iPSC, UNK: *SAMHD1* (cHET), c.869G>A (p.R290H); c.1642C>T (p.Q548*)<br>2. Pd iPSC, UNK: *SAMHD1* (HMZ), c.499C>T (p.H167YP)<br>3. Pd iPSC, UNK: *SAMHD1* (HMZ), c.490C>T (p.R164*)	Sendai virus (Fibroblasts, PBMCs)	Hanchen et al. 2022
		1. Pd iPSC, UNK: *ADAR1* (HET), c.3019G>A (p.G1007R)<br>2. Pd iPSC, UNK: *ADAR1* (HET), c.3019G>A (p.G1007R)	Episomal vectors (PBMCs)	Garcia et al. 2024

		3. Pd iPSC, UNK: *ADAR1* (HET), c.3019G>A (p.G1007R)		
CLCN2-LD	*CLCN2*	1. Pd iPSC, UNK: *CLCN2* (HMZ), c.2257C>T (p.R753*)	Episomal vectors (urine cells)	Chen et al. 2020
**Leuko-axonopathies**				
TUBB4A-LD	*TUBB4A*	1. Pd iPSC, UNK: *TUBB4A* (HET), c.745G>A (p.D249N)<br>2. Pd iPSC, UNK: *TUBB4A* (HET), c.745G>A (p.D249N)<br>3. Pd iPSC, UNK: *TUBB4A* (HET), c.745G>A (p.D249N)	Sendai virus (Fibroblasts, PBMCs)	Almad et al. 2023
LBSL	*DARS2*	1. Pd iPSC, UNK: *DARS2* (cHET), c.228-21_228-20delinsC (p.R76fs); c.518A>G (p.Y173C)	Episomal vectors (PBMCs)	Zhang et al. 2022
ARSAL	*MARS2*	1. Pd iPSC, UNK: *MARS2* (cHET), c.550C>T (p.Q184*); c.424C>T (p.R142W)	Sendai virus (Fibroblasts)	Salemi et al. 2025
GM1	*GLB1*	1. Pd iPSC, UNK: *GLB1* (cHET), c.523C>T (p.Q175*); c.574T>C>T (p.Y192H)	Episomal vectors (PBMCs)	Guan et al. 2024
		1. Pd iPSC, Infantile GM1: *GLB1* (cHET), c.380G>A; c.481T>G	Sendai virus (Fibroblasts)	Rha et al. 2024
GM2	*HEXA*	1. Pd iPSC, Infantile GM2: *HEXA* (cHET), c.1278insTATC; c.IVS12+1G>C	Lentiviral (Fibroblasts)	Liu et al. 2016
MPS	*IDUA, IDS, SGSH, NAGLU, HGSNAT, GNS, GUSB*	1. Pd iPSC, Unaffected carrier: *IDS* (HET), c.85C>T	Lentiviral (PBMCs)	Varga et al. 2016
		1. Pd iPSC, Mild MPS type II: *IDS* (HEMI), c.182C>T (p.S61F)	Lentiviral (PBMCs)	Varga et al. 2016
		1. Pd iPSC, Severe MPS type II (Hunter): *IDS* (HEMI), c.85C>T (p.Q29*)	Lentiviral (PBMCs)	Varga et al. 2016
		1. Pd iPSC, Severe MPS type II (Hunter): *IDS* (HEMI), c.85C>T (p.Q29*)	Lentiviral (PBMCs)	Varga et al. 2016
		1. Pd iPSC, MPS type III B (Sanfilippo): *NAGLU* (HMZ), c.1073C>T (p.P358L)	Episomal plasmids (Fibroblasts)	Vallejo-Diez et al. 2018
		1. Pd iPSC, MPS type III A (Sanfilippo): *SGSH* (cHET), c.1351G>A (p.E447K); c.746G>A (p.R245H)	Episomal plasmids (Fibroblasts)	Vallejo et al. 2018
		1. GE iPSC: *HGSNAT* (cHET), c.195_210del (p.N66Gfs*15); c.207_208insCA (p.Y70Hfs*17)[ab]<br>2. GE iPSC: *HGSNAT* (cHET), c.195_210del (p.N66Gfs*15); c.209delinsGAATG (p.Y70*)[ac]	Retrovirus (Fibroblasts)	Beneto et al. 2019
		1. Pd iPSC, MPS type II (Hunter): *IDS* (HEMI), c.208insC (p.H70Pfs*29)[ae]	Sendai virus (Fibroblasts)	Hong et al. 2019
		1. Pd iPSC, MPS type III B (Sanfilippo): *NAGLU* (HMZ), c.457G>A (p.E153K)[ad]	Sendai virus (Fibroblasts)	Huang et al. 2019
		1. Pd iPSC, MPS type I (Hurler): *IDUA* (cHET), c.1073_1093del (p.H358_T364del); c.1205G>A (p.W402*)[z]	Sendai virus (Fibroblasts)	Lito et al. 2019
		1. Pd iPSC, Attenuated MPS I: *IDUA* (HMZ), c.266G>A (p.R89Q)	Episomal vectors (PBMCs)	Suga et al. 2019
		1. GE iPSC: *NAGLU* (HMZ), p.F288_I290del<br>2. GE iPSC: *NAGLU* (HMZ), p.F288Ifs*27	Retrovirus (UNK)	Beneto et al. 2020

		1. Pd iPSC, MPS type III B (Sanfilippo), *NAGLU* (cHET), c.1336G>A; c.608G>A	Episomal vectors (PBMCs)	Guan et al. 2021
		1. Pd iPSC, MPS type III B (Sanfilippo), *NAGLU* (HMZ), c.1876C>T (p.R626*)	Sendai virus (Fibroblasts)	Rodriguez-Lopez et al. 2024
		1. Pd iPSC, MPS type II (Hunter), *IDS* (HEMI), c.418+495_1006+1304	Sendai virus (PBMCs)	Lee et al. 2025
		1. Pd iPSC, MPS type III A (Sanfilippo): *SGSH* (cHET), c.706G>A (p.D235N); c.449G>A (p.R150Q)	Sendai virus (PBMCs)	Lee et al. 2025
NCL	*CLN1 – CLN13*	1. Pd iPSC, UNK: *CLN5* (cHET), c.335G>A (p.R112H); c.619T>C (p.W207R)<br>2. Pd iPSC, UNK: *CLN5* HMZ), c.1072-1073delTT (p.L358Afs*4)	Sendai virus (Fibroblasts)	Ofrim et al. 2024
		1. Pd iPSC, Syndromic: *CLN3* (cHET), c.461-280_677+38del; c.1056+3A>C<br>2. Pd iPSC, Vision-only: *CLN3* (cHET), c.1001G>A; c.1213C>T<br>3. Pd iPSC, Vision-only: *CLN3* (cHET), c.1001G>A; c.1213C>T<br>4. Pd iPSC, Syndromic: *CLN3* (HMZ), c.461-280_677+382del<br>5. Pd iPSC, Syndromic: *CLN3* (cHET), c.1001G>A; g. g.(28211763_28486693)_ (28488725_28488776)del<br>6. Pd iPSC, Vision-only: *CLN3* (cHET), c. c.461-280_677+382del; c.461-3C>G	Fibroblasts (Sendai virus)	Dwojak et al. 2024
**Microgliopathies**				
PLOSL	*TREM2*	1. Pd, iPSC, UNK: *TREM2* (cHET), c.313del (p.A105fs); c.199del (p.H67fs)	Sendai virus (PBMCs)	Gottert et al. 2025
CSF1R-LD	*CSF1R*	1. Pd iPSC, late-onset CSF1R-LD: *CSF1R* (HET), c.2512G>C (p.V838L)	Episomal vectors (Fibroblasts)	Hayer et al. 2018
		1. Pd iPSC, UNK: *CSF1R* (HET), c.2381T>C (p.I794T)	Episomal vectors (PBMCs)	Wu et al. 2021
		1. GE iPSC: *CSF1R* (HET), KO<br>2. GE iPSC: *CSF1R* (HMZ), KO	Episomal plasmids (Fibroblasts)	Schmitz et al. 2023
**Leuko-vasculopathies**				
CADASIL	*NOTCH3*	1. Pd iPSC, UNK: *NOTCH3* (HET), c.3724C>T (p.R1242C)	Sendai virus (PBMCs)	Fernandez-Susavila et al. 2018
		1. Pd iPSC, UNK: *NOTCH3* (HET), c.994C>T (p.R332C)<br>2. Pd iPSC, UNK: *NOTCH3* (HET), c.505C>T (p.R169C)<br>3. Pd iPSC, UNK: *NOTCH3* (HET), c.697T>G (p.C233G)<br>4. Pd iPSC, UNK: *NOTCH3* (HET), c.665G>A (p.C222Y)<br>5. Pd iPSC, UNK: *NOTCH3* (HET), c.1364G>A (p.C455T)	Sendai virus (PBMCs)	Chen et al. 2020
		1. Pd iPSC, UNK: *NOTCH3* (HET), c.397C>T (p.R133C)	Sendai virus (Fibroblasts)	Aygar et al. 2025

Abbreviations: UNK: unknown; NR: not reported; NA: not applicable; HMZ, homozygous; cHET: compound heterozygous; HEMI: hemizygous; iPSC: induced pluripotent stem cells; ESC: embryonic stem cells; GE: genetically engineered; X-ALD: Adrenoleukodystrophy; MLD: Metachromatic

leukodystrophy; CTX: Cerebrotendinous xanthomatosis; PMD: Pelizaeus-Merzbacher disease; POLR3-HLD: POLR3-related leukodystrophy; AHDS: Allan-Herndon-Dudley syndrome; Bloc1s1-LD: Bloc1s1-related leukodystrophy; CLDN11-HLD: CLDN11-related leukodystrophy; FSASD: Free Sialic Acid Storage Disorders; PKU: Phenylketonuria; Cx32-CMT: Cx32-related (X-linked) Charcot-Marie-Tooth disease; FAHN: Fatty acid hydroxylase-associated neurodegeneration; AGS: Aicardi-Goutières syndrome; CLCN2-LD: CLCN2-related leukoencephalopathy; TUBB4A-LD: TUBB4A-related leukodystrophy; LBSL: Leukoencephalopathy with brainstem and spinal cord involvement and lactate elevation; ARSAL: Autosomal recessive spastic ataxia with leukoencephalopathy; GM1: GM1 gangliosidosis; GM2: GM2 gangliosidosis; MPS: Mucopolysaccharidoses; NCL: Neuronal ceroid lipofuscinoses; PLOSL: Polycystic lipomembranous osteodysplasia with sclerosing leukoencephalopathy; CSF1R-LD: CSF1R-related leukoencephalopathy; CADASIL: Cerebral autosomal dominant arteriopathy with subcortical infarcts and leukoencephalopathy.

Genetic information is written as was published in the original article.

[a]Letters indicate if the iPSCs utilized in different studies are derived from the same individual.

PRISMA-ScR Flow Diagram

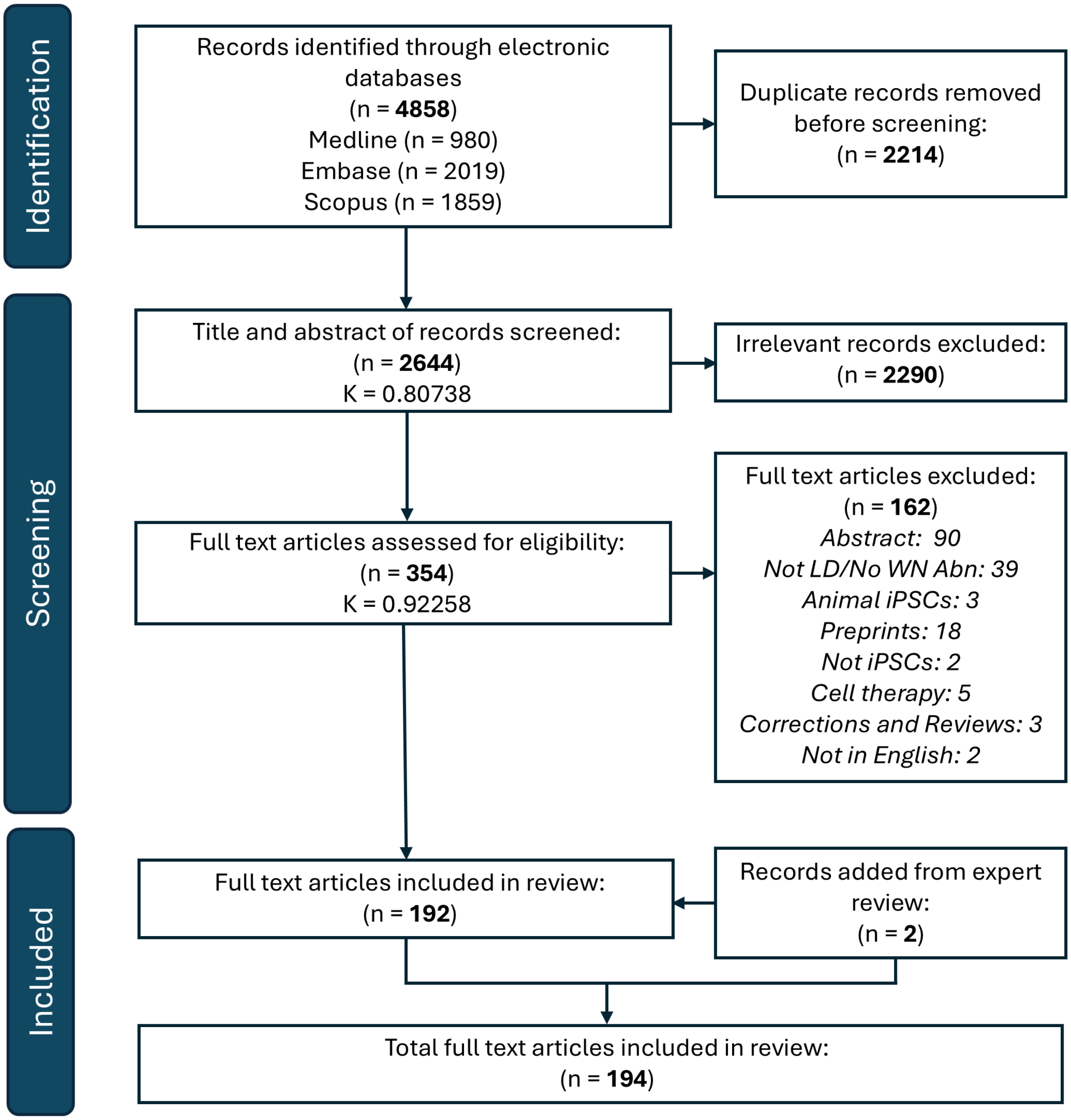

Supplementary Fig. 1: PRISMA-ScR flow diagram illustrating the systematic process of study selection for this review, using data extracted from Covidence. The diagram outlines the identification, screening, and inclusion stages, starting with 4858 records retrieved from electronic databases (Ovid Medline, Embase, and Scopus). After removing duplicates (n=2214) and irrelevant records (n=2290), 354 full-text articles were assessed for eligibility. Reasons for exclusion at this stage included disorders that are not leukodystrophies (n=39), iPSCs derived from mouse models (n=3), and other criteria as specified in the diagram. A total of 192 articles were included in the review, supplemented by 2 additional records obtained from expert recommendations, resulting in 194 full-text articles analyzed.

SUPPLEMENTARY REFERENCES

1 Son, D. *et al.* Generation of two induced pluripotent stem cell (iPSC) lines from X-linked adrenoleukodystrophy (X-ALD) patients with adrenomyeloneuropathy (AMN). *Stem Cell Res* **25**, 46-49 (2017). https://doi.org:10.1016/j.scr.2017.10.003

2 You, Y. R., Son, D., Kang, P. J., You, S. & Kim, D. S. Generation of induced pluripotent stem cell (iPSC) line from a 21-year-old X-linked adrenoleukodystrophy (X-ALD) patient. *Stem Cell Res* **25**, 136-138 (2017). https://doi.org:10.1016/j.scr.2017.10.016

3 Yeon, G. B. *et al.* Generation of an induced pluripotent stem cell (iPSC) line from a 42-year-old adult cerebral type X-linked adrenoleukodystrophy (X-ALD) patient. *Stem Cell Res* **36**, 101425 (2019). https://doi.org:10.1016/j.scr.2019.101425

4 Wang, L., Gao, B., Mo, X., Guo, X. & Huang, J. Generation of an urine-derived induced pluripotent stem cell line from a 6-year old X-linked adrenoleukodystrophy (X-ALD) patient. *Stem Cell Res* **51**, 102170 (2021). https://doi.org:10.1016/j.scr.2021.102170

5 Kuramochi, Y. *et al.* Generation of two human induced pluripotent stem cell lines derived from two X-linked adrenoleukodystrophy patients with ABCD1 mutations. *Stem Cell Res* **53**, 102337 (2021). https://doi.org:10.1016/j.scr.2021.102337

6 Sik Jung, E. *et al.* Generation of mutation-corrected induced pluripotent stem cell lines derived from adrenoleukodystrophy patient by using homology directed repair. *Stem Cell Res* **59**, 102664 (2022). https://doi.org:10.1016/j.scr.2022.102664

7 Wang, Q. H. *et al.* Generation and characterization of induced pluripotent stem cell lines derived from skin fibroblasts of patients with adrenoleukodystrophy. *Stem Cell Res* **73**, 103243 (2023). https://doi.org:10.1016/j.scr.2023.103243

8 Gornostal, E., Alsalloum, A., Mityaeva, O. & Volchkov, P. Generation of induced pluripotent stem line (MIPTi001-A) derived from patient with X-linked adrenoleukodystrophy (X-ALD). *Stem Cell Res* **74**, 103298 (2024). https://doi.org:10.1016/j.scr.2023.103298

9 Wang, W., Lv, Y. F., Zhang, Y. J., Dong, W. J. & Zhang, Y. Generation of a human induced pluripotent stem cell line PUMCi001-A from a patient with Krabbe disease. *Stem Cell Res* **48**, 101937 (2020). https://doi.org:10.1016/j.scr.2020.101937

10 Hoflinger, P. *et al.* Induced pluripotent stem cells (iPSCs) derived from cerebrotendinous xanthomatosis (CTX) patient's fibroblasts carrying a R395S mutation. *Stem Cell Res* **17**, 433-436 (2016). https://doi.org:10.1016/j.scr.2016.09.010

11 Kim, K. P. *et al.* Generation of a human iPSC line (MPIi006-A) from a patient with Pelizaeus-Merzbacher disease. *Stem Cell Res* **46**, 101839 (2020). https://doi.org:10.1016/j.scr.2020.101839

12 Liu, B. *et al.* Generation of the human induced pluripotent stem cell line (ZJUi005-A) from a patient with Pelizaeus-Merzbacher disease (PMD) carrying a novel hemizygous mutation in PLP1 gene. *Stem Cell Res* **45**, 101791 (2020). https://doi.org:10.1016/j.scr.2020.101791

13 Schreiber, M. K. & Zafeiriou, M. P. Generation of Pelizaeus-Merzbacher disease (PMD) mutant (PLP1-C33Y) in induced pluripotent stem cell (iPSC) by CRISPR/Cas9 genome editing. *Stem Cell Res* **74**, 103276 (2024). https://doi.org:10.1016/j.scr.2023.103276

14 Casamassa, A. *et al.* Production of an induced pluripotent stem cell line CSSi018-A (14192) from a patient with hypomyelinating leukodystrophy 7 (HLD7) carrying biallelic variants of POLR3A (c.1802 T > A; c.4072G > A). *Stem Cell Res* **78**, 103468 (2024). https://doi.org:10.1016/j.scr.2024.103468

15 Manibarathi, K. *et al.* An iPSC model for POLR3A-associated spastic ataxia: Generation of three unrelated patient cell lines. *Stem Cell Res* **76**, 103363 (2024). https://doi.org:10.1016/j.scr.2024.103363
16 Martins, S. *et al.* Generation of an induced pluripotent stem cell line (IUFi001) from a Cockayne syndrome patient carrying a mutation in the ERCC6 gene. *Stem Cell Res* **55**, 102456 (2021). https://doi.org:10.1016/j.scr.2021.102456
17 Wang, A. *et al.* Generation of an induced pluripotent stem cell line (SHCDNi003-A) from a one-year-old Chinese Han infant with Allan-Herndon-Dudley syndrome. *Stem Cell Res* **46**, 101872 (2020). https://doi.org:10.1016/j.scr.2020.101872
18 Ludwik, K. A. *et al.* Generation of iPSC lines with SLC16A2:G401R or SLC16A2 knock out. *Stem Cell Res* **73**, 103256 (2023). https://doi.org:10.1016/j.scr.2023.103256
19 Wu, K. *et al.* Generation of human induced pluripotential stem cells from individuals with complex heterozygous, isogenic corrected, and homozygous Bloc1s1 mutations. *Stem Cell Res* **64**, 102905 (2022). https://doi.org:10.1016/j.scr.2022.102905
20 Ozgoren, O. K. *et al.* Generation of a human induced pluripotent stem cell line from a patient with hypomyelinating leukodystrophy 22 (HLD22). *Stem Cell Res* **71**, 103174 (2023). https://doi.org:10.1016/j.scr.2023.103174
21 Sabir, M. S. *et al.* Generation and characterization of two iPSC lines derived from subjects with Free Sialic Acid Storage Disorder (FSASD). *Stem Cell Res* **81**, 103600 (2024). https://doi.org:10.1016/j.scr.2024.103600
22 Liu, N. *et al.* Human induced pluripotent stem cell line (SDQLCHi064-A) derived from a patient with Canavan disease carrying c.556_559dup GTTC and c.919delA mutations in the ASPA gene. *Stem Cell Res* **76**, 103325 (2024). https://doi.org:10.1016/j.scr.2024.103325
23 Xu, T. *et al.* Generation of integration-free induced pluripotent stem cell line (NJMUi001-A) from a phenylketonuria patient. *Stem Cell Res* **25**, 179-182 (2017). https://doi.org:10.1016/j.scr.2017.11.008
24 Qi, Z. *et al.* Generation of urine-derived induced pluripotent stem cells from a patient with phenylketonuria. *Intractable Rare Dis Res* **7**, 87-93 (2018). https://doi.org:10.5582/irdr.2018.01032
25 Veleva, D. *et al.* Generation of fibroblast-derived induced pluripotent stem cell (iPSC) lines from two paediatric patients with phenylketonuria. *Stem Cell Res* **77**, 103405 (2024). https://doi.org:10.1016/j.scr.2024.103405
26 Veleva, D. *et al.* Generation of two lymphoblastoid-derived induced pluripotent stem cell (iPSC) lines from patients with phenylketonuria. *Stem Cell Res* **77**, 103407 (2024). https://doi.org:10.1016/j.scr.2024.103407
27 Son, D., Kang, P. J., Yun, W. & You, S. Generation of induced pluripotent stem cell (iPSC) line from a 36-year-old Charcot-Marie-Tooth disease patient with GJB1 mutation (CMTX). *Stem Cell Res* **21**, 9-12 (2017). https://doi.org:10.1016/j.scr.2017.03.006
28 Efendic, F. *et al.* Generation of the human iPSC line AKOSi010-A from fibroblasts of a female FAHN patient, carrying the compound heterozygous mutation p.Gly45Arg/p.His319Arg. *Stem Cell Res* **63**, 102863 (2022). https://doi.org:10.1016/j.scr.2022.102863
29 Efendic, F. *et al.* Generation of the human iPSC lines AKOSi011-A carrying the mutation p.Pro65Ser/p.Asp35T and AKOSi012-A, carrying the mutation p.Tyr231His, derived from FAHN patient fibroblasts. *Stem Cell Res* **71**, 103178 (2023). https://doi.org:10.1016/j.scr.2023.103178
30 Ferraro, R. M. *et al.* Establishment of three iPSC lines from fibroblasts of a patient with Aicardi Goutieres syndrome mutated in RNaseH2B. *Stem Cell Res* **41**, 101620 (2019). https://doi.org:10.1016/j.scr.2019.101620

31 Ferraro, R. M. *et al.* Generation of three iPSC lines from fibroblasts of a patient with Aicardi Goutieres Syndrome mutated in TREX1. *Stem Cell Res* **41**, 101580 (2019). https://doi.org:10.1016/j.scr.2019.101580
32 Masneri, S. *et al.* Generation of three isogenic induced Pluripotent Stem Cell lines (iPSCs) from fibroblasts of a patient with Aicardi Goutieres Syndrome carrying a c.2471G>A dominant mutation in IFIH1 gene. *Stem Cell Res* **41**, 101623 (2019). https://doi.org:10.1016/j.scr.2019.101623
33 Fuchs, N. V. *et al.* Generation of three induced pluripotent cell lines (iPSCs) from an Aicardi-Goutieres syndrome (AGS) patient harboring a deletion in the genomic locus of the sterile alpha motif and HD domain containing protein 1 (SAMHD1). *Stem Cell Res* **43**, 101697 (2020). https://doi.org:10.1016/j.scr.2019.101697
34 Hanchen, V. *et al.* Generation of induced pluripotent stem cell lines from two patients with Aicardi-Goutieres syndrome type 1 due to biallelic TREX1 mutations. *Stem Cell Res* **64**, 102895 (2022). https://doi.org:10.1016/j.scr.2022.102895
35 Hanchen, V. *et al.* Generation of induced pluripotent stem cell lines from three patients with Aicardi-Goutieres syndrome type 5 due to biallelic SAMDH1 mutations. *Stem Cell Res* **64**, 102912 (2022). https://doi.org:10.1016/j.scr.2022.102912
36 Garcia, L. *et al.* Generation of three induced pluripotent stem cell lines from individuals with Aicardi-Goutieres syndrome caused by a c.3019G>A (p.G1007R) autosomal dominant pathogenic variant in ADAR1. *Stem Cell Res* **74**, 103299 (2024). https://doi.org:10.1016/j.scr.2023.103299
37 Chen, Z. *et al.* Generation of an iPSC line (SKLOi001-A) from a patient with CLCN2-related leukoencephalopathy. *Stem Cell Res* **45**, 101769 (2020). https://doi.org:10.1016/j.scr.2020.101769
38 Almad, A. A. *et al.* Generation of three induced Pluripotent Stem Cell lines from individuals with Hypomyelination with Atrophy of Basal Ganglia and Cerebellum caused by a c.745G>A (p.D249N) autosomal dominant mutation in TUBB4A. *Stem Cell Res* **69**, 103083 (2023). https://doi.org:10.1016/j.scr.2023.103083
39 Zhang, A., Lu, J. & Xiao, F. An induced pluripotent stem cell line (FHUSTCi002-A) derived from a patient with leucoencephalopathy with brain stem and spinal cord involvement and lactate elevation. *Stem Cell Res* **63**, 102872 (2022). https://doi.org:10.1016/j.scr.2022.102872
40 Salemi, S. E. *et al.* Generation of induced pluripotent stem cell line ISMMSi060-A from a patient with combined oxidative phosphorylation deficiency 25. *Stem Cell Res* **83**, 103662 (2025). https://doi.org:10.1016/j.scr.2025.103662
41 Guan, J. *et al.* Establishment of iPS cell line (SDQLCHi080-A) from a patient with GM1 gangliosidosis due to GLB1 mutation. *Stem Cell Res* **81**, 103545 (2024). https://doi.org:10.1016/j.scr.2024.103545
42 Rha, A. K. *et al.* Generation of an infantile GM1 gangliosidosis induced pluripotent stem cell line (CHOCi005-A) for disease modeling and therapeutic testing. *Stem Cell Res* **81**, 103552 (2024). https://doi.org:10.1016/j.scr.2024.103552
43 Liu, Z. & Zhao, R. Generation of HEXA-deficient hiPSCs from fibroblasts of a Tay-Sachs disease patient. *Stem Cell Res* **17**, 289-291 (2016). https://doi.org:10.1016/j.scr.2016.08.010
44 Varga, E. *et al.* Generation of human induced pluripotent stem cell (iPSC) line from an unaffected female carrier of Mucopolysaccharidosis type II (MPS II) disorder. *Stem Cell Res* **17**, 514-516 (2016). https://doi.org:10.1016/j.scr.2016.09.035

45 Varga, E. *et al.* Generation of Mucopolysaccharidosis type II (MPS II) human induced pluripotent stem cell (iPSC) line from a 1-year-old male with pathogenic IDS mutation. *Stem Cell Res* **17**, 482-484 (2016). https://doi.org:10.1016/j.scr.2016.09.033
46 Varga, E. *et al.* Generation of Mucopolysaccharidosis type II (MPS II) human induced pluripotent stem cell (iPSC) line from a 3-year-old male with pathogenic IDS mutation. *Stem Cell Res* **17**, 479-481 (2016). https://doi.org:10.1016/j.scr.2016.09.032
47 Varga, E. *et al.* Generation of Mucopolysaccharidosis type II (MPS II) human induced pluripotent stem cell (iPSC) line from a 7-year-old male with pathogenic IDS mutation. *Stem Cell Res* **17**, 463-465 (2016). https://doi.org:10.1016/j.scr.2016.09.034
48 Vallejo-Diez, S., Fleischer, A., Martin-Fernandez, J. M., Sanchez-Gilabert, A. & Bachiller, D. Generation of two induced pluripotent stem cells lines from a Mucopolysaccharydosis IIIB (MPSIIIB) patient. *Stem Cell Res* **33**, 180-184 (2018). https://doi.org:10.1016/j.scr.2018.10.019
49 Vallejo, S. *et al.* Generation of two induced pluripotent stem cells lines from Mucopolysaccharydosis IIIA patient: IMEDEAi004-A and IMEDEAi004-B. *Stem Cell Res* **32**, 110-114 (2018). https://doi.org:10.1016/j.scr.2018.09.009
50 Beneto, N. *et al.* Generation of two compound heterozygous HGSNAT-mutated lines from healthy induced pluripotent stem cells using CRISPR/Cas9 to model Sanfilippo C syndrome. *Stem Cell Res* **41**, 101616 (2019). https://doi.org:10.1016/j.scr.2019.101616
51 Hong, J. *et al.* Generation of an induced pluripotent stem cell line (TRNDi008-A) from a Hunter syndrome patient carrying a hemizygous 208insC mutation in the IDS gene. *Stem Cell Res* **37**, 101451 (2019). https://doi.org:10.1016/j.scr.2019.101451
52 Huang, W. *et al.* An induced pluripotent stem cell line (TRNDi006-A) from a MPS IIIB patient carrying homozygous mutation of p.Glu153Lys in the NAGLU gene. *Stem Cell Res* **37**, 101427 (2019). https://doi.org:10.1016/j.scr.2019.101427
53 Lito, S. *et al.* Generation of human induced pluripotent stem cell line UNIGEi001-A from a 2-years old patient with Mucopolysaccharidosis type IH disease. *Stem Cell Res* **41**, 101604 (2019). https://doi.org:10.1016/j.scr.2019.101604
54 Suga, M. *et al.* Generation of a human induced pluripotent stem cell line, BRCi001-A, derived from a patient with mucopolysaccharidosis type I. *Stem Cell Res* **36**, 101406 (2019). https://doi.org:10.1016/j.scr.2019.101406
55 Beneto, N. *et al.* Generation of two NAGLU-mutated homozygous cell lines from healthy induced pluripotent stem cells using CRISPR/Cas9 to model Sanfilippo B syndrome. *Stem Cell Res* **42**, 101668 (2020). https://doi.org:10.1016/j.scr.2019.101668
56 Guan, J. *et al.* Induced pluripotent stem cell line (SDQLCHi041-A) from a male patient with mucopolysaccharidosis type IIIB. *Stem Cell Res* **52**, 102212 (2021). https://doi.org:10.1016/j.scr.2021.102212
57 Rodriguez-Lopez, A. *et al.* Generation of an induced pluripotent stem cell line (TRNDi042-A) from a Mucopolysaccharidosis type IIIB patient with homozygous p. R626X (c. 1876C > T) mutation in the NAGLU gene. *Stem Cell Res* **81**, 103612 (2024). https://doi.org:10.1016/j.scr.2024.103612
58 Lee, N., Noh, H. & Cheon, C. K. Human induced pluripotent stem cell line (PNUSCRi005-A) generated from severe type of Hunter syndrome patient carrying exonic deletion (exon 4-7 del) in in human iduronate 2-sulfatase gene. *Stem Cell Res* **83**, 103639 (2025). https://doi.org:10.1016/j.scr.2024.103639
59 Lee, N., Noh, H. & Cheon, C. K. Human induced pluripotent stem cell line (PNUSCRi006-A) derived from a patient with Sanfilippo syndrome type A exhibiting a mutation in SGSH gene. *Stem Cell Res* **84**, 103690 (2025). https://doi.org:10.1016/j.scr.2025.103690

60 Ofrim, M. *et al.* Characterization of two human induced pluripotent stem cell lines derived from Batten disease patient fibroblasts harbouring CLN5 mutations. *Stem Cell Res* **74**, 103291 (2024). https://doi.org:10.1016/j.scr.2023.103291
61 Dwojak, E. *et al.* Six induced pluripotent stem cell lines from fibroblasts of individuals with CLN3-related conditions. *Stem Cell Res* **81**, 103563 (2024). https://doi.org:10.1016/j.scr.2024.103563
62 Gottert, R. *et al.* Generation of a human induced pluripotent stem cell line (BIHi292-A) from PBMCs of a female patient diagnosed with Nasu-Hakola disease (NHD)/polycystic lipomembranous osteodysplasia with sclerosing leukoencephalopathy (PLOSL) carrying a novel heterozygous mutation in the TREM2 gene. *Stem Cell Res* **83**, 103660 (2025). https://doi.org:10.1016/j.scr.2025.103660
63 Hayer, S. N., Schelling, Y., Hoeflinger, P., Hauser, S. & Schols, L. Generation of an induced pluripotent stem cell line from a patient with adult-onset leukoencephalopathy with axonal spheroids and pigmented glia (ALSP): HIHCNi003-A. *Stem Cell Res* **30**, 206-209 (2018). https://doi.org:10.1016/j.scr.2018.06.011
64 Wu, J., Tian, W., Zhan, F., Luan, X. & Cao, L. Generation of an human induced pluripotent stem cell JTUi007-A from a patient with CSF1R-related leukoencephalopathy carrying heterozygous p.Ile794Thr mutation in CSF1R gene. *Stem Cell Res* **57**, 102593 (2021). https://doi.org:10.1016/j.scr.2021.102593
65 Schmitz, A. S. *et al.* Generation of a heterozygous and a homozygous CSF1R knockout line from iPSC using CRISPR/Cas9. *Stem Cell Res* **69**, 103066 (2023). https://doi.org:10.1016/j.scr.2023.103066
66 Fernandez-Susavila, H. *et al.* Generation and characterization of the human iPSC line IDISi001-A isolated from blood cells of a CADASIL patient carrying a NOTCH3 mutation. *Stem Cell Res* **28**, 16-20 (2018). https://doi.org:10.1016/j.scr.2018.01.023
67 Chen, G. *et al.* Generation of human induced pluripotent stem cells (NIHTVBi004-A, NIHTVBi005-A, NIHTVBi006-A, NIHTVBi007-A, NIHTVBi008-A) from 5 CADASIL patients with NOTCH3 mutation. *Stem Cell Res* **45**, 101821 (2020). https://doi.org:10.1016/j.scr.2020.101821
68 Aygar, S. & Daheron, L. Generation of a human iPSC line with Notch3 R133C mutation by CRISPR/Cas9: A tool for investigating CADASIL and therapeutic targets. *Stem Cell Res* **84**, 103678 (2025). https://doi.org:10.1016/j.scr.2025.103678
